# Effects of water-soluble components of atmospheric particulates from rare earth mining areas in China on lung cancer cell cycle

**DOI:** 10.1186/s12989-021-00416-z

**Published:** 2021-08-02

**Authors:** Yuan Xia, Xulong Zhang, Dejun Sun, Yumin Gao, Xiaoe Zhang, Li Wang, Qingjun Cai, Qihao Wang, Juan Sun

**Affiliations:** 1 School of Public Health, Inner Mongolia Autonomous Region, Jinshan Economic and Technological Development Zone, Inner Mongolia Medical University, Inner Mongolia Autonomous Region 010010 Hohhot, China; 2 Department of Immunology, School of Basic Medical Sciences, Capital Medical University, Beijing, China; 3 Inner Mongolia People’s Hospital, Inner Mongolia Autonomous Region Hohhot, China

**Keywords:** Atmospheric particulates from rare earth mine, Lung cancer cell, Cell cycle, Quantitative proteomics, Ribosomal proteins

## Abstract

**Background:**

This study aims to investigate the effects of water soluble particulate matter (WSPM) on the viability and protein expression profile of human lung adenocarcinoma cell A549 in the Bayou Obo rare earth mining area, and explore the influence of WSPM on the A549 cell cycle.

**Results:**

It was found that WSPM can inhibit the viability of A549 cells and induce cell arrest in the G2/M phase. Compared with controls, exposure to WSPM10 and WSPM2.5 induced 134 and 116 proteins to be differentially expressed in A549 cells, respectively. In addition, 33 and 31 differentially expressed proteins were further confirmed, and was consistent with the proteomic analysis. The most prominent enrichment in ribosome-associated proteins were presented. When *RPL6*, *RPL13*, or *RPL18A* gene expression was inhibited, A549 cells were arrested in the G1 phase, affecting the expression of *Cyclin D1*, *p21*, *RB1*, *Cyclin A2*, *Cyclin B1*, *CDC25A*, *CDK2*, *CHEK2* and *E*_*2*_*F*_*1*_. Furthermore, the La^3+^, Ce^3+^, Nd^3+^ and F^-^ in WSPM also inhibited the viability of A549 cells. After 24 h of exposure to 2 mM of NaF, A549 cells were also arrested in the G2/M phase, while the other three compounds did not have this effect. These four compounds affected the cell cycle regulatory factors in A549 cells, mainly focusing on effecting the expression of *CDK2*, *CDK4*, *RB1*, *ATM*, *TP53* and *MDM2* genes. These results are consistent with the those from WSPM exposure.

**Conclusions:**

These results revealed that WSPM from rare earth mines decreased the viability of A549 cells, and induced cell cycle G2/M phase arrest, and even apoptosis, which may be independent of the NF-κB/MYD88 pathway, and be perceived by the TLR4 receptor. The dysfunction of the cell cycle is correlated to the down-expression of ribosomal proteins (RPs). However, it is not the direct reason for the A549 cell arrest in the G2/M phase. La^3+^, Ce^3+^, and F^-^ are probably the main toxic substances in WSPM, and may be regulate the A549 cell cycle by affecting the expression of genes, such as *MDM2, RB1, ATM, TP53, E*_*2*_*F*_*1*_, *CDK2* and *CDK4*. These results indicate the importance for further research into the relationship between APM and lung cancer.

**Supplementary Information:**

The online version contains supplementary material available at 10.1186/s12989-021-00416-z.

## Background

Mining and smelting activities inevitably result in atmospheric particulate matter (APM) pollution and health hazard problems. Particulate matter contains a large amount of chemical elements, which include various toxic substances, such as heavy metals, rare earth elements (REE), radioactive elements, and polycyclic aromatic hydrocarbons (PAHs) [1]. Regardless of the in-depth epidemiological studies, the potential mechanisms of APM-related adverse health effects are not fully understood [2, 3]. The chemical composition of APM strongly depends on the geographic location and anthropogenic activities. Furthermore, many of these chemical components which are toxic and carcinogenic make numerous deleterious effects to human health [4]. In order to better understand the biological mechanisms involved in APM-associated cytotoxicity, special focus was given on the role of REE mine areas. The particulate matter samples were collected in Bayan Obo Mining District which is a mining town in west Inner Mongolia, China. This region has the world’s largest deposits of rare-earth metals [5]. Large-scale mining, smelting and processing, air drying, less rain and strong wind have led to serious air particulate matter pollution in Bayan Obo, and a high REE background value in the atmospheric environment [6]. Tong et al. have found that the PM_10_ of Bayan Obo inhibited A549 cell viability, induced reactive oxygen species (ROS) production, and caused significant DNA damage [5]. In addition to the above mechanisms of cytotoxicity, it remains unclear whether there are other toxicity mechanisms.

In the present study, the potential toxicity of the water soluble particulate matter (WSPM) in A549 cells was investigated using approaches for molecular biology and cell biology. The toxicity effects of WSPM10 have been discerned on A549 cells by proteomics technology [7, 8]. This has the potential to identify previously unknown biomarkers, in order to gain insights into the mechanisms of toxicity. To our knowledge, the present study is the first time to provide details on the cytotoxicity of the WSPM in the Bayan Obo mining area.

## Results

### Chemical compositions and sources of WSPM in the Baotou Bayan Obo mining area

**Fig. 1:**
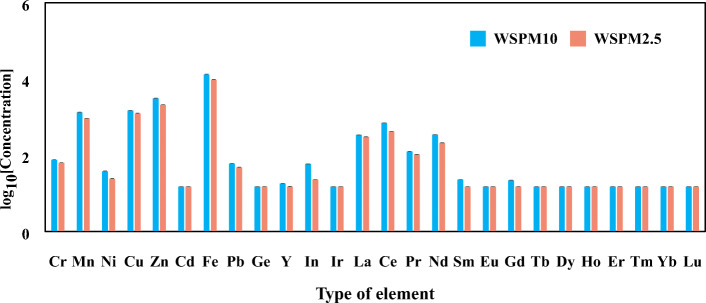
Content of metallic elements in WSPM10 and WSPM2.5. The abscissa represents the element species, while the ordinate represents the mass concentrations (μg/g) of the element (in log scale) in WSPM10 or WSPM2.5. The metallic elements of the samples were determined using inductively coupled plasma-atomic emission spectrometry. The power was 1.4 KW, the carrier gas flow rate was 1.0 L/min, the coolant flow was 15 L/min, the auxiliary flow was 1.0 L/min, and the pump speed was 1.5 L/min

**Fig. 2:**
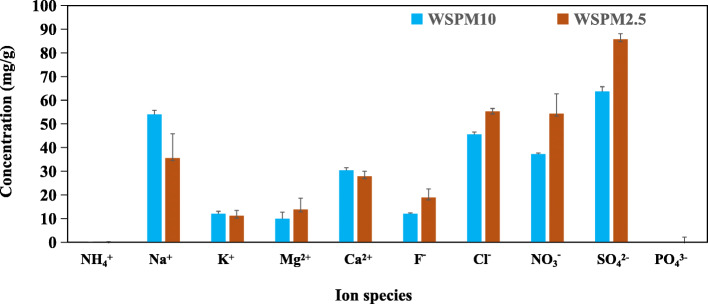
Content of water soluble ions in WSPM10 and WSPM2.5. The abscissa represents the ion species, while the ordinate represents the ion mass concentration (mg/g) in WSPM10 or WSPM2.5. The determination of water soluble ion by suppressed ion chromatography was described in the samples

In WSPM10 and WSPM2.5, eight rare earth elements, seven heavy metal elements and nine water-soluble ions were detected. As shown in Fig. 1, the order of average concentration of REE in WSPM10 was as follows: Ce > Nd > La > Pr > In > Sm > Gd > Y. The order of average concentrations of heavy metal elements was as follows: Fe > Zn > Cu > Mn > Cr > Pb > Ni. As shown in Fig. 2, the five kinds of cationic average concentrations were as follows: Na^+^ > Ca^2+^ > K^+^ > Mg^2+^ > NH_4_^+^. The average concentrations of the four anions presented in the following order: SO_4_^2−^ > Cl^−^ > NO_3_^−^ > F^−^. In WSPM2.5, the average mass concentration and distribution rule of REE and heavy metal elements were similar to that of WSPM10, but were lower than that of WSPM10 as a whole. Furthermore, its average concentration of anion and cation was higher than WSPM10. Among the detected ions SO_4_^2−^ is the most abundant chemical component of both WSPM10 and WSPM2.5.

### WSPM inhibited A549 cell viability

**Fig. 3:**
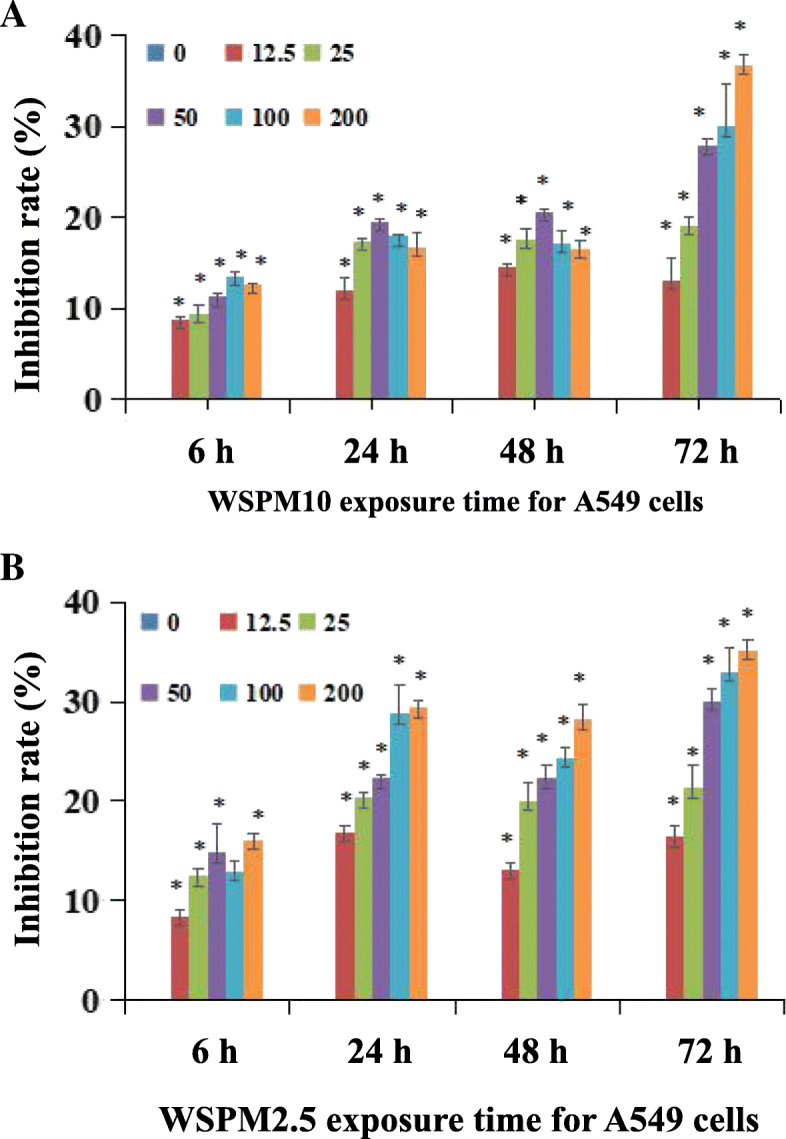
The effect of WSPM on A549 cell viability. The abscissa represents the exposure time of WSPM to A549 cells, while the ordinate represents the cell viability inhibition rate (%). A549 cells were treated by different concentrations (0, 12.5, 25, 100 and 200 µg/ml) of WSPM10 (**A**) and WSPM2.5 (**B**) for 6, 24, 48 and 72 h, and cell viability was determined by MTT. The optical density value (OD) of samples were measured at 550 nm. The cell viability inhibition rate = (1 - [OD of infected cells - background value] / [OD of control - background value]) × 100 %. The values shown on top of the bars represent the average of three individual experiments, and the standard errors are shown * WSPM with different concentrations was compared with the control group (0 µg/ml) at the same exposure time, *P* < 0.01

After the incubation of A549 cells with WSPM for 6, 24, 48 and 72 h, the viability of A549 cells was significantly reduced compared with controls (*P* < 0.01). As shown in Fig. 3 A, after treatment with WSPM10 on A549 cells for 6 h, with increasing dose from 0 to 100 µg/ml the inhibition rate of cell viability also increased the dose of WSPM10 increased from 0 to 100 µg/ml, and the inhibition rate of cell viability also increased. The inhibition rate reached nearly 15 % when the dose reached 100 µg/ml (*P* < 0.01). However, when the dose increased to 200 µg/ml, the cell viability inhibition rate decreased to a certain extent (*P* < 0.01). When the incubation time of WSPM10 was 24 or 48 h, the dose of WSPM10 increased from 0 to 50 µg/ml, and the cell viability inhibition rate reached approximately 20 % (*P* < 0.01). When exposure time was 72 h, the inhibition rate of cell viability continued to increase with the increase in WSPM10 dose, and it reached nearly 40 %. When the WSPM10 dose was 200 µg/ml, the inhibition rate of cell viability depended on the time of exposure.

As shown in Fig. 3B, after 6 h of incubation, the inhibition rate increased with the increase in WSPM2.5 dose, and the cell viability recovered to a certain extent when the concentration was 100 µg/ml, but the difference was not statistically significant (*P* > 0.05). When the incubation time was 24, 48 and 72 h, the inhibition rate of A549 cell viability significantly increased with the increase in WSPM2.5 concentration, showing a dose-response relationship (*P* < 0.01). When the incubation time was 24 or 48 h, compared with WSPM10 at the same dose, the WSPM2.5 group exhibited a stronger inhibition effect on cell viability as a whole.

### A549 cell cycle arrest in the G2/M phase was induced by WSPM

**Fig. 4:**
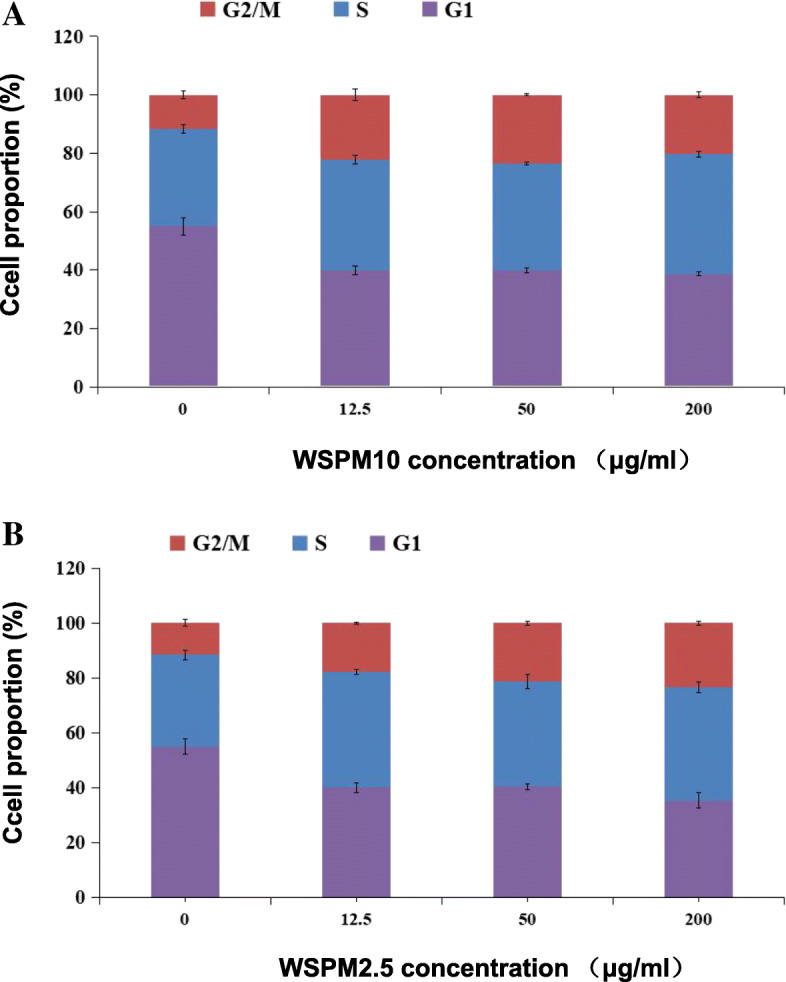
Influence of the A549 cell cycle distribution treatment with different doses WSPM**.** (**A**) When A549 cells were treated with different concentrations of WSPM10, the cell proportion decreased in the G1 phase, while this increased in the G2/M phase. Cell cycle were arrested at the G2/M phase; (**B**) B shows the same effect as A. The values shown on top of the bars represent the average of three individual experiments, and the standard errors are shown

To study the effects on cell cycle, A549 cells were treated with WSPM for 24 h. With the increasing doses of WSPM, the proportion of G2/M cells rised, while G1 cells decreased. This indicates that WSPM induced the cell cycle arrest of A549 cells at the G2/M phase (Fig. 4, S1 and S2). G2/M arrest may be one of the reasons why WSPM inhibits the viability of A549 cells. Figures S1C, S1D and S2D present the obvious sub-G1 peak. With the increasing doses of WSPM, the signal of this peak became more obvious, indicating that some cells had apoptosis or necrosis during the exposure process. In general, A549 cells were exposed to different doses of WSPM for 24 h, and each dose group exhibited a decrease in the proportion of G1 phase cells, and an increase in the proportion of G2/M phase cells (*P* < 0.05).

### Cluster analysis of differentially expressed proteins(DEPs)

**Fig. 5:**
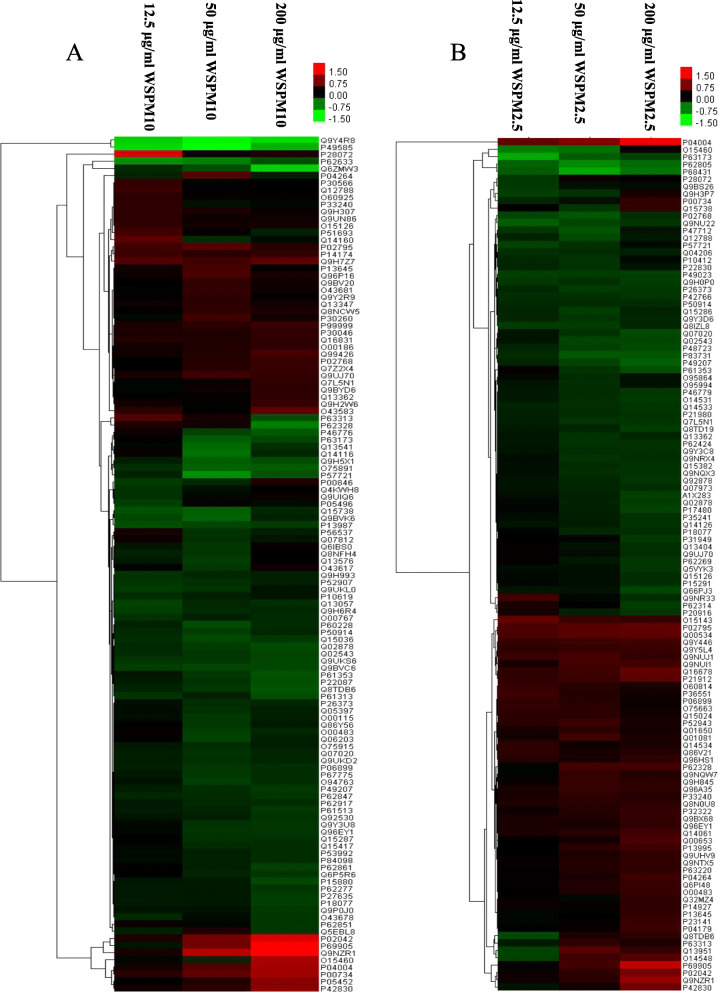
Heatmap analysis of differential expression proteins. Heatmap analysis was used to analyze the DEPs with similar expression trends under exposure to WSPM10 (**A**) and WSPM2.5 (**B**) at concentrations of 12.5, 50 and 200 µg/ml, respectively. The small panel with color in the figure represents the changes in protein abundance from downregulated (green) to upregulated (red)

In order to explore the cytotoxic effect on A549 cells during WSPM exposure, DEPs were screened by high-throughput isobaric Tags for Relative and Absolute Quantitation (iTRAQ)-based quantitative proteomic technology. A total of 2,453 proteins were identified and quantified with > 95 % confidence interval (CI) and false discovery rate (FDR) of < 1.0 %, and proteins with no less than one unique peptide were considered as positive identification. A 1.20-fold cutoff was established to detect DEPs (Figures S3 and S4). Afterwards, cluster analysis was performed to obtain the heatmap, which contains the dysregulated proteins (Fig. 5). Figure 5 A and 5B present the clustering heatmaps of A549 cells exposed to WSPM10 and WSPM2.5 at different doses for 24 h, respectively.

It could be concluded that A549 cells exposed to different concentrations of WSPM10 correspond to different number DEPs. 27, 56, and 63 DEPs were observed in the 12.5, 50.0, and 200.0 µg/ml WSPM10 group, respectively. The cellular component (CC) analysis shows that the identified proteins mainly distributed in the cytosol, extracellular membrane-bounded organelles, cytosolic ribosomes, and so on. In the molecular function (MF) classification, the identified proteins that work as protein binding, poly(A) RNA binding and structural constituent of ribosomes which ranked at the top of the category. In the biological process (BP) category, proteins participated in the synthesis of nucleotides and purines, and performed functions, such as nucleic acid binding transcription factors and specific DNA binding transcription factors for the low dose group of WSPM10 (12.5 µg/ml). When the dose of WSPM10 was 50 µg/ml, the majority of the identified proteins were classified into cell translation, mRNA synthesis, intracellular protein localization, and the disintegration of certain macromolecular substances. MF mainly focus on ribosome structural factors, structural molecular activities, iron ion binding proteins and heme binding proteins. When the dose of WSPM10 was further increased to 200 µg/ml, the biological enrichment pathway was similar to that of the 50 µg/ml group, and the MF mainly focused on ribosome structural composition factors, structural molecular activity, and RNA binding proteins (Figure S5; Tables S9, S10 and S11).

When the A549 cells were exposed to WSPM2.5 at a low dose (12.5 µg/ml), the DEPs were mainly distributed in the endoplasmic reticulum, nucleosome chromosomes and ribosomes, and most of the proteins participated in the response to external toxic substances, metal particles, nutrients, inorganic substances, nucleosome assembly, and chromosome assembly. The main molecular function was binding proteins. When the concentration of WSPM2.5 was 50 µg/ml, the DEPs were mainly concentrated in the cytoplasm, especially in large molecular complexes, such as ribosomes, and most of which were involved in the synthesis, assembly, localization and disassembly of intracellular proteins, and in the synthesis of mRNA. Molecular functions are mainly focused on the binding of transition metals, and the assembly of ribosomes and RNA binding proteins. When the concentration of WSPM2.5 was 200 µg/ml, the enrichment pathway was similar to that of the 50 µg/ml group, and the MF mainly focused on ribosome structural factors and metal-binding proteins (Figure S6; Tables S12, S13 and S14).

By comparing the different dose groups of the same particle, the WSPM dose significantly influenced the number and types of DEPs. At low doses (12.5 µg/ml), DEPs were mainly enriched in the response to nutrients and the function of nucleotide anabolism. However, with the increase in WSPM dose (50 µg/ml), the RNA catabolism mechanism and enrichment of cell alienation function *in vivo* manifested. When the dose was 200 µg/ml, the external stimulation exceeded the tolerance limit of the cell, and the stress response was inadequate for the pressure of the external environment, which damages the structure of large molecules, such as DNA and protein, and the ion channel dysfunction, resulting in the change in cell membrane potential. In addition, rare earth or heavy metal elements in particulate matter bind with intracellular proteins, and inactivate these. All these changes in biological functions based on the GO enrichment analysis, but the specific process needs to be further verified.

### Significant enrichment pathway analysis of DEPs

DEPs were annotated into the KEGG database to obtain the involved signaling pathways or biological metabolic pathways. Among these, a total of 23 pathways were involved in the 12.5 µg/ml WSPM10 group, and the enrichment pathways were arranged according to their significance from high to low (*P* < 0.05), as follows: the renin-angiotensin system, the fanconi anemia pathway, and the steroid biosynlife pathway (Table S15). The 50 µg/ml WSPM10 group involved 75 pathways. According to the significance (*P* < 0.05), the enrichment of pathways, in descending order, was as follows: the ribosome, malaria, african trypanosomiasis, tuberculosis, staphylococcus aureus infection, virus causing cancer, the p53 signaling pathway, muscle atrophy, amyotrophic lateral sclerosis, colorectal cancer, apoptosis, RNA transshipment, and ErbB signaling pathways (Table S1^6^). A total of 57 pathways were involved in the 200 µg/ml WSPM10 group, and the enrichment pathways were arranged from high to low, as follows: ribosome, complement and coagulation cascade (Table S17).

A total of 91 pathways were involved in the 12.5 µg/ml WSPM2.5 group, and the enrichment pathways were arranged from high to low, as follows: systemic lupus erythematosus, alcohol poisoning, virus causing cancer, cholesterol synthesis, cancer disorders, the vascular endothelial growth factor (VEGF) signaling pathway, linoleic acid metabolism, chemokine signaling pathways, alpha linoleic acid metabolism, Ras signaling pathways, steroid biosynthesis, inflammatory bowel disease, the silk crack original activated protein kinase (MAPK) signaling pathway, human T lymphocyte virus type-1 infection, cocaine addiction, and steroid hormone biosynthesis (Table S18). The 50 µg/ml WSPM2.5 group involved 57 pathways. The significant enrichment of channel was as follows (from high to low): the ribosome, alpha linoleic acid metabolism, the metabolism of cholesterol synthesis, linoleic acid, the phosphatidyl inositol 3 kinase/protein kinase B (PI3K/Akt) signaling pathway, and VEGF signaling pathways (Table S19). The 200 µg/ml WSPM2.5 group involved 77 pathways. The enrichment of channel, in descending order, was as follows: the ribosome, steroid biosynthesis, systemic lupus erythematosus (SLE), peroxisome, staphylococcal infections, glycosaminoglycan biosynthesis, and glycosphingolipid biosynthesis (Table S20).

According to the significant enrichment pathway analysis above, the most significant pathway was the ribosome pathway when the dose was 50 µg/ml and 200 µg/ml WSPM2.5 or WSPM10. Therefore, the DEPs involved in this pathway were selected as the objects of the follow-up studies.

### Verification of DEPs

In the iTRAQ and multiple reaction monitoring (MRM) results, DEPs with the same expression trend were considered to meet the verification requirements, and the change value of the expression level was greater than 1.2 and *P* < 0.05. Tables S3 and S4 illustrates the proteins detected using the MRM method in groups with different doses of WSPM10 and WSPM2.5, respectively, which met the verification requirements. Table S5 and Figure S11 presents the DEPs verified by western blot in the WSPM10 and WSPM2.5 groups.

### The downregulated expression of intracellular RPs affected the A549 cell cycle, but was an indirect factor of the G2/M arrest

**Fig. 6:**
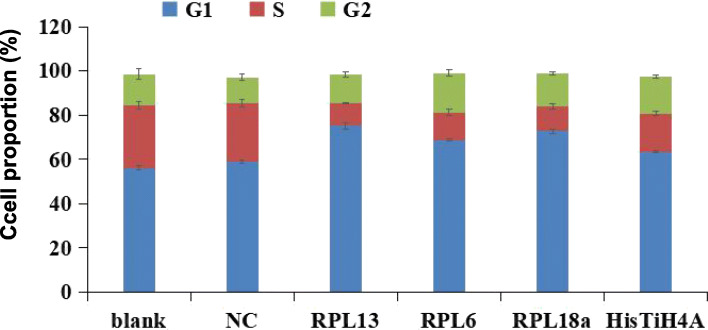
Influence of gene interference on A549 cell cycle distribution. For all the interference groups, the cell proportion increased at the G1 phase, while this decreased at the S-phase. After the target gene was interfered, the cell cycle was arrested at the G1 phase. In the siRNA RPL13, siRNA RPL6 and siRNA RPL18A group, the proportion of A549 cells in the G1 phase was 75.08 ± 2.21 %, 68.72 ± 0.63 % and 72.75 ± 1.62 %, respectively, which was higher than that in the blank group (56.19 ± 1.74 %) and NC group (59.73 ± 0.66 %) (*P* < 0.01). The number of cells in the G1 phase in the siRNA HIS1H4A group was 63.41 ± 0.94 %, but the difference was not statistically significant, when compared to the NC group (*P* > 0.05). Meanwhile, in the siRNA RPL13, siRNA RPL6, siRNA RPL18A and siRNA HIS1H4A groups, the proportion of A549 cells in the S phase was 10.12 ± 0.40 %, 12.62 ± 2.60 %, 11.27 ± 1.75 %, and 17.31 ± 1.84 %, respectively, which was lower than those in the blank group (28.22 ± 3.01 %) and NC group (26.20 ± 2.98 %) (*P* < 0.05)

According to the iTRAQ and MRM validation results, *RPL6*, *RPL18A* and *RPL13* were all downregulated under WSPM10 and WSPM2.5 exposure conditions. HIS1H4A was also downregulated, but was only detected in the WSPM2.5 exposed group. In order to investigate their effects on the A549 cell cycle, siRNA interference with the expression of these genes were used to observe the relationship between their downregulated expression and the A549 cell cycle. As presented in Figure S8, compared with the negative control (NC) group, the transfection of siRNA *RPL6*-1, siRNA *RPL18a*-1 and siRNA *RPL13*-1 significantly reduced the levels of *RPL6*, *RPL18A* and *RPL13* mRNA, and the inhibition rate reached over 90 % (*P* < 0.05). After transfection with siRNA *HIS1H4A*-3, *HIS1H4A* mRNA level decreased by > 80 % (*P* < 0.05). Therefore, siRNA *RPL6*-1, siRNA *RPL18a*-1, siRNA *RPL13*-1 and siRNA *HIS1H4A*-3 were selected for functional studies in subsequent cell transfection experiments. Compared with the NC group, the distribution of the A549 cell cycle before and after the interference of the four genes, there was no statistically significant difference in the proportion of cells in the G2/M phase (*P* > 0.05, Fig. 6). As presented in Figure S8 and Table S6, and compared with the NC group, when the *RPL13*, *RPL6* and *RPL18A* gene expression was disturbed, the A549 cell cycle was arrested at the G1 phase (*P* < 0.01). Furthermore, the proportion of cells decreased in the S phase (*P* < 0.05). This suggests that when *RPL13*, *RPL6* and *RPL18A* genes are disturbed, G1 phase arrest is induced in A549 cells. The proportion of cells in the S phase decreased in all gene interference groups (*P* < 0.05).

**Fig. 7:**
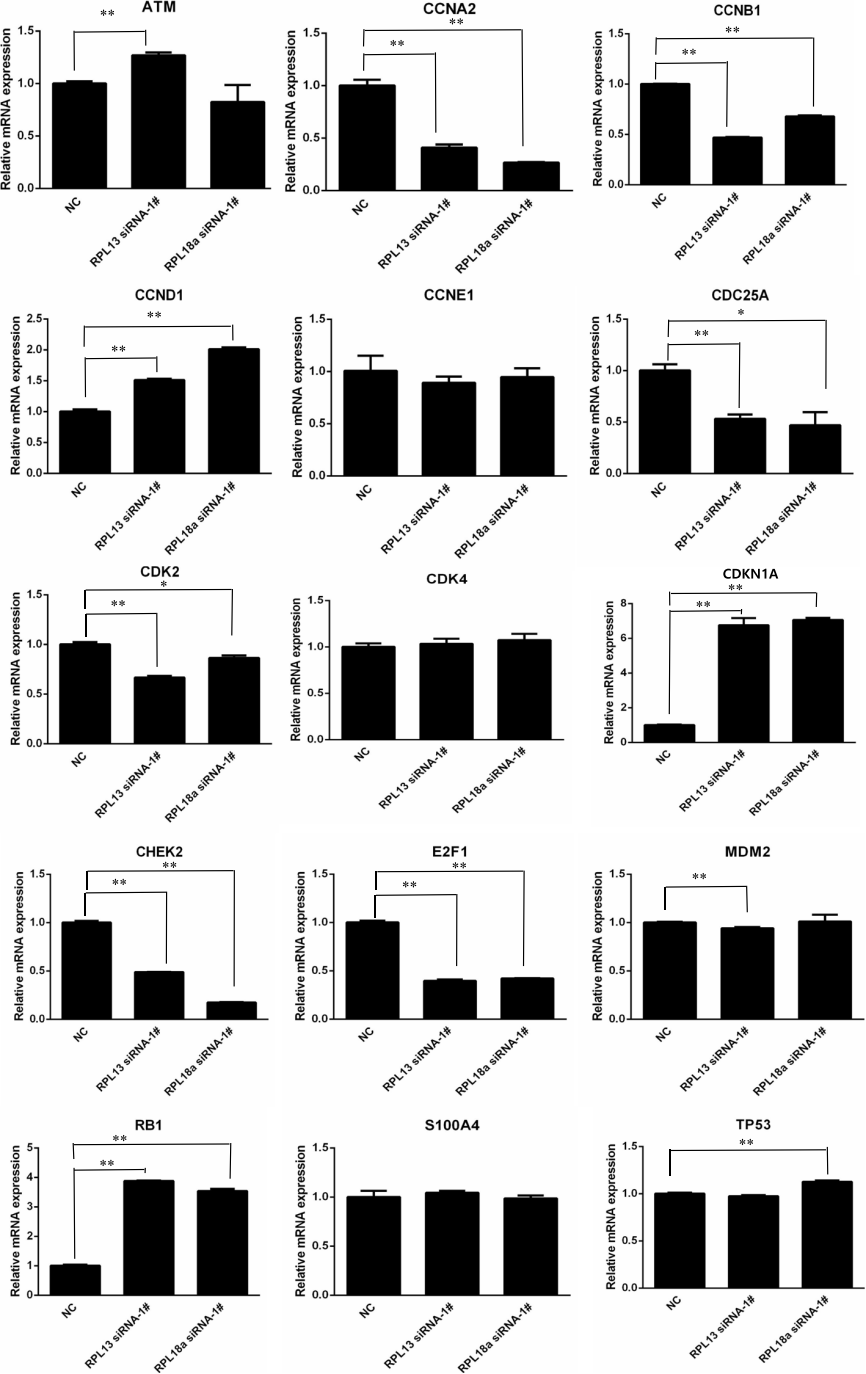
Effects of ***RPL13*** and ***RPL18A*** gene interference on the A549 cell cycle regulator expression. The abscissa represents the NC, siRNA RPL13 and siRNA RPL18A group, respectively, while the ordinate represents the relative expression quantity of the corresponding genes. The expression levels of 15 cell cycle regulators were measured by qRT-PCR at 24 h after siRNA transfection. *Compared with the NC group, *P* < 0.05; **Compared with the NC group, *P* < 0.01

In previous validation experiments, it was confirmed that RPL13, RPL18A and RPL6 proteins were downregulated in A549 cells under the WSPM exposure process. Therefore, the downregulation of *RPL13* and *RPL18A* genes effected the cell cycle regulation gene expression. As shown in Fig. 7, the expression of *CCND1*, *CDKN1A* and *RB1* were upregulated. Compared with the NC group, the expression levels of *CCND1*, *CDKN1A* and *RB1* in the siRNA RPL13 group increased by 1.5, 6.5, and 4.0 times, respectively (*P* < 0.01). In the siRNA RPL18A group, the expression of *CCND1*, *CDKN1A* and *RB1* increased by 2.0, 7.0, and 3.5 times, respectively (*P* < 0.01). In addition, *CCNA2*, *CCNB1*, *CDC25A*, *CDK2*, *CHEK2* and *E*_*2*_*F*_*1*_ were downregulated in both groups. However, the expression of the *TP53* gene increased by 12.5 % in the siRNA RPL18A group (*P* < 0.01), and was not significantly different from the other group (*P* > 0.05). Compared with the NC group, there were no significant differences in *CCNE1*, *CDK4* and *S100A4* (*P* > 0.05). When the *RPL13* and *RPL18A* gene expression was disturbed, and compared with the NC group, the *CCND1*, *CDKN1A* and *RB1* expression increased, while the *CCNA2*, *CCNB1*, *CDC25A*, *CDK2*, *CHEK2* and *E*_*2*_*F*_*1*_ expression decreased. In the siRNA RPL13 group, compared with the NC group, ATM expression increased, while MDM2 expression decreased, and the differences were statistically significant (*P* < 0.01). In the siRNA RPL18A group, compared with the NC group, the expression of TP53 increased (*P* < 0.01).

### F^−^, La^3+^, and Ce^3+^ may be the main components of WSPM that causes cell cycle disturbance

**Fig. 8:**
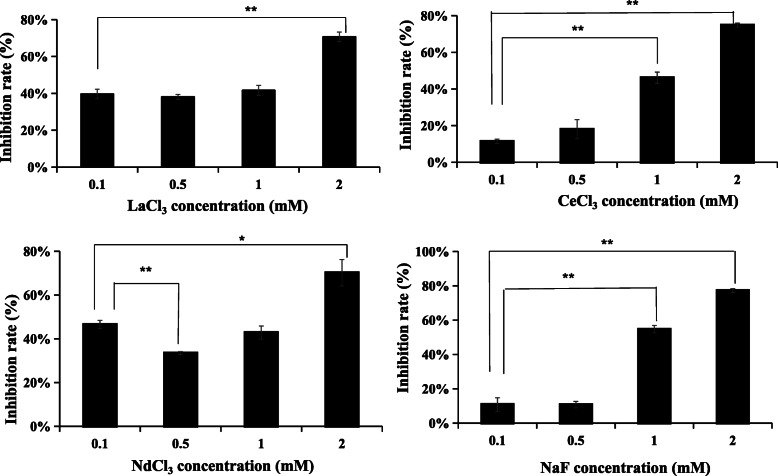
Effects of LaCl_3_, CeCl_3_, NdCl_3_ and NaF on the viability of A549 cells. The abscissa represents the exposure concentration, while the ordinate represents the cell viability inhibition rate (%). A549 cells were treated with LaCl_3_, CeCl_3_, NdCl_3_ and NaF at the same concentration gradient (0, 0.1, 0.5, 1, and 2 mM) for 24 h, respectively. *Compared with the dose group at 0.1 mM, *P* < 0.05; **Compared with the dose group at 0.1 mM, *P* < 0.01

In order to screen for major chemical risk factors in WSPM that cause A549 cell cycle disturbance, LaCl_3_, CeCl_3_, NdCl_3_ and NaF were selected as research subjects to observe their effects on A549 cell viability. Figure 8 shows that after the incubation of these four inorganic compounds for 24 h, the viability of A549 cells significantly decreased, compared with the controls (*P* < 0.01, Table S7).

**Fig. 9:**
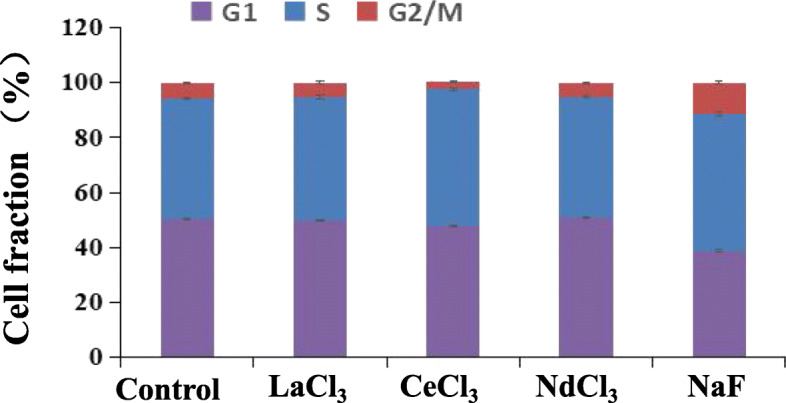
Influence of LaCl_3_, CeCl_3_, NdCl_3_ and NaF on the A549 cell cycle distribution. When A549 cells were treated with 2 mM of NaF for 24 h, the cell cycle arrest was induced at the G2/M phase

After A549 cells were treated with LaCl_3_, CeCl_3_, NdCl_3_ and NaF for 24 h, compared with the control group, the LaCl_3_ and NdCl_3_ exposed groups exhibited a slight change in cell proportion at the G1, S and G2/M phases, but the difference was not statistically significant (*P* > 0.05). For the CeCl_3_ exposed group, the proportion of G1 phase cells exhibited a small decrease (*P* < 0.05), while the proportion of S phase cells increased (*P* < 0.01). Although the proportion of G2/M phase cells exhbited a decrease, the difference was not statistically significant (*P* > 0.05). For the NaF exposed group, compared with the controls, the proportion of G1 phase cells decreased by 11.62 %, while the proportion of cells at the S and G2/M phase increased by 6.31 and 5.30 %, respectively (*P* < 0.01; Fig. 9, Table S8).

**Fig. 10:**
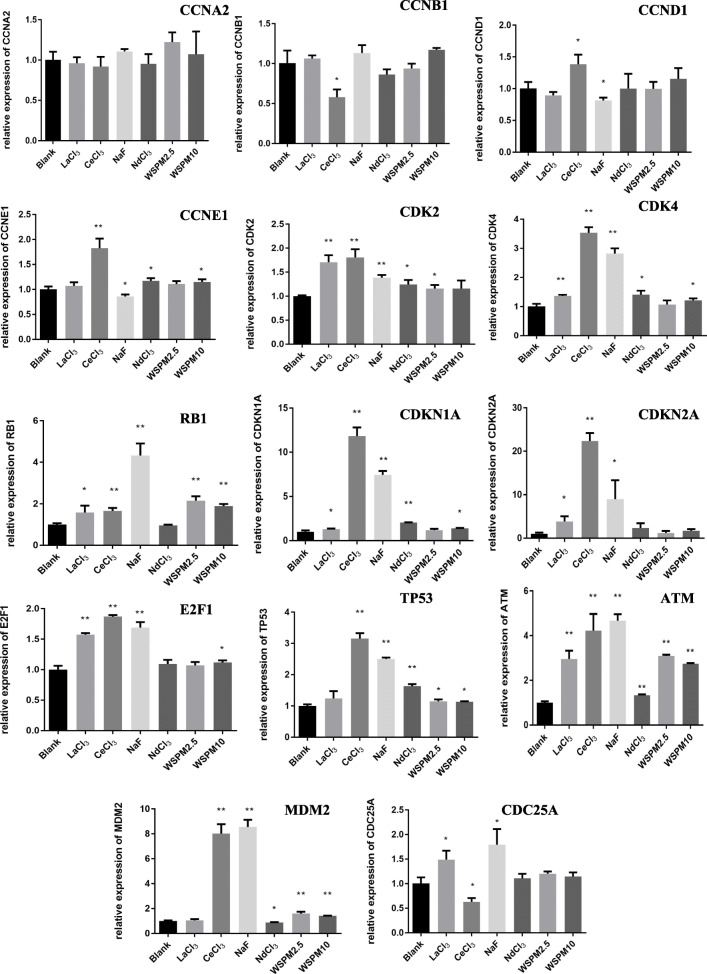
Effects of WSPM, LaCl_3_, CeCl_3_, NdCl_3_ and NaF on the A549 cell cycle regulator mRNA expression. The abscissa represents the blank control, 2 mM of LaCl_3_, 2 mM of CeCl_3_, 2 mM of NdCl_3_, 2 mM of NaF, 200 µg/ml of WSPM2.5, and 200 µg/ml of WSPM10, from left to right, respectively, and the ordinate represents the relative expression quantity of the corresponding genes. A549 cells were treated with the above substances for 24 h, and the expression of 14 kinds of cell cycle regulators were measured by qRT-PCR. *Compared with the blank group, *P* < 0.05; **Compared with the blank group, *P* < 0.01

**Fig. 11:**
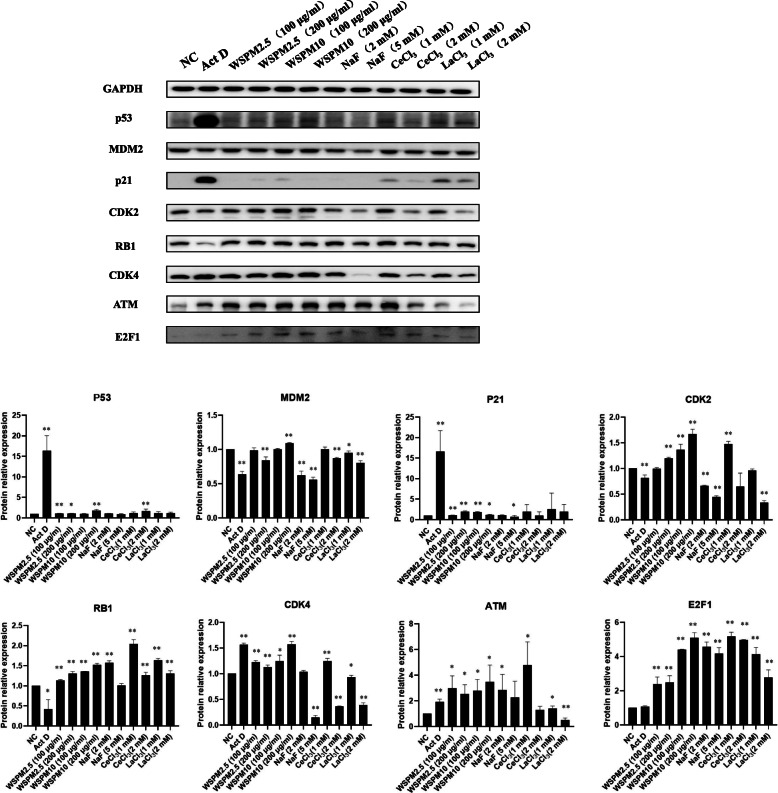
Effects of WSPM, LaCl_3_, CeCl_3_, NdCl_3_ and NaF on the A549 cell cycle regulator protein expression. A549 cells were treated with WSPM2.5 (100 µg/ml or 200 µg/ml) or WSPM10 (100 µg/ml or 200 µg/ml) or NaF (2 mM or 5 mM) or CeCl_3_ (1 mM or 2 mM) or LaCl_3_ (1 mM or 2 mM) for 24 h. Cell lysates were immunoblotted with the indicated antibodies. A549 cells treated with either DMSO or 5 nM actinomycin D were used as negative control (NC) and positive control, respectively. Each experiment was repeated three times independently. *Compared wih the NC group, *P* < 0.05; **Compared with the NC group, *P* < 0.01

In order to assess if the exposure to WSPM are associated with the expression of cell cycle regulators in A549 cells, the regulators which directly involved in the regulation of the cell cycle, were measured during WSPM exposure. As shown in Fig. 10, the *ATM* gene expression was upregulated in all exposure groups. The *ATM* mRNA levels of the LaCl_3_, CeCl_3_, NaF, NdCl_3_, WSPM2.5 and WSPM10 exposed groups were increased by 2.96, 4.22, 4.67, 1.33, 3.09, and 2.74 times compared with the control, respectively (*P* < 0.01). In addition, it was found that mRNA levels of *RB1*, *TP53* and *MDM2* were upregulated in the WSPM2.5 and WSPM10 groups (*P* < 0.05). The expression trend of cell cycle regulators in the NaF exposure group, was consistent with the WSPM2.5 and WSPM10 groups, which was mainly in *MDM2*, *RB1*, *ATM*, and *TP53* genes. The mRNA levels of these genes were all upregulated (*P* < 0.05), among which the NaF group had a significantly higher change amplitude, compared with WSPM groups. On the other hand, the LaCl_3_, CeCl_3_ and NdCl_3_ groups also exhibited similarities in the mRNA expression trends of cell cycle regulators, which were mainly in the *CDK2*, *CDK4*, *CDKN1A* and *ATM* genes. Among these, the CeCl_3_ exposed group included 13 genes (11 upregulated and 2 downregulated genes), and 8 and 7 genes in the LaCl_3_ and NdCl_3_ exposed groups, respectively, which exhibited an upregulated expression (*P* < 0.05). In the LaCl_3_ and CeCl_3_ exposed groups, 7 genes exhibited the same expression trend, namely, *CDK2*, *CDK4*, *RB1*, *CDKN1A*, *CDKN2A*, *E*_*2*_*F*_*1*_ and *ATM*. Meanwhile, the mRNA expression trend of *CDC25A* was the opposite. However, the mRNA expression trends of 7 genes in the NdCl_3_ exposed group were completely the same as those in the CeCl_3_ group, namely, *CDK2*, *CDK4*, *CCNE1*, *CDKN1A*, *TP53*, *MDM2* and *ATM*. As presented from the above results, LaCl_3_, CeCl_3_, NaF, NdCl_3_, WSPM2.5 and WSPM10 have many similarities in their effects on the mRNA expression of cell cycle regulators, and have some characteristics of their own. As shown in Fig. 11 and Tabel S22, the protein level of ATM, CDK4, CDK2, E_2_F_1_, p21, p53 and RB1 were upregulated in WSPM10 and WSPM2.5 exposure groups. This result is consistent in the transcription level. Conversely, MDM2 was decreased in WSPM2.5 exposure groups, but was slightly increased in WSPM10 exposure groups. On the other hand, ribosomal stress is induced by the impairment of ribosome biogenesis. The exposure of actinomycin D (Act D) induced ribosomal stress, including the dysfunction of ribosome-free RPs and there RPs is involved in a multiple cellular processes such as MDM2-p53 signaling pathway and so on [9]. To investigate the changes in there cell cycle regulators levels induced by ribosomal stress which is caused by WSPM exposure, we treated cells with 5 nM Act D for ribosomal stress positive control group. The protein levels of ATM, CDK4, p21, and p53 were upregulated sharply in Act D exposure groups, the protein levels of MDM2 and RB1 were decreased. At the same time, the protein level of E_2_F_1_, p21, p53, and RB1 were upregulated in CeCl_3_, and LaCl_3_ exposure groups. The expression of ATM, E_2_F_1_, MDM2, p21, and RB1 proteins was upregulated in 2 mM NaF exposure groups. It was consistent with their mRNA expression trends in 2mM NaF exposure groups. But the increasing of there proteins level did not show a dose-dependent manner.

**Fig. 12:**
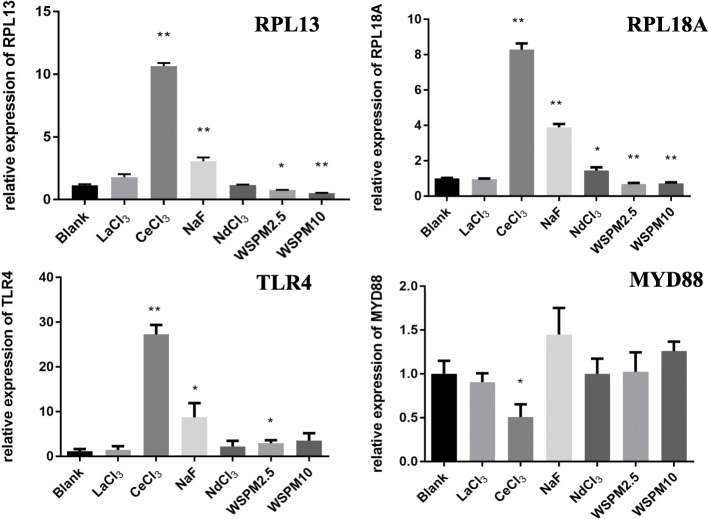
Effects of WSPM, LaCl_3_, CeCl_3_, NdCl_3_ and NaF on the expression of RPL13, RPL18A, TLR4 and MYD88. The abscissa represents the blank control, 2 mM of LaCl_3_, 2 mM of CeCl_3_, 2 mM of NdCl_3_, 2 mM of NaF, 200 µg/ml of WSPM2.5, and 200 µg/ml of WSPM10, from left to right, respectively, and the ordinate represents the relative expression quantity of the corresponding genes. A549 cells were treated with the above substances for 24 h, and the expression of RPL13, RPL18A, TLR4 and MYD88 were measured by qRT-PCR. *Compared wih the blank group, *P* < 0.05; **Compared with the blank group, *P* < 0.01

In addition, the effects of WSPM, LaCl_3_, CeCl_3_, NdCl_3_ and NaF on the expression of *RPL13*, *RPL18A*, *TLR4* and *MYD88* genes were investigated (Fig. 12). It was found that the CeCl_3_ and NaF exposure groups resulted in the upregulation of *RPL13* gene expression, which increased by 10.66 and 3.07 times, respectively (*P* < 0.01). But in the WSPM10 and WSPM2.5 groups, it was reduced by 22.6 % (*P* < 0.05) and 49.0 % (*P* < 0.01), respectively. The expression of *RPL13* gene was no different between the LaCl_3_ and NdCl_3_ exposure groups. For *RPL18A*, an upregulated gene was found in the CeCl_3_, NdCl_3_ and NaF exposure groups, which was increased by 8.29, 3.88 and 1.45 times, respectively. However, in the WSPM10 and WSPM2.5 exposure groups, *RPL18A* mRNA level decreased by 37 % (*P* < 0.01) and 27 % (*P* < 0.01), respectively. On the other hand, the expression changes of *TLR4*, *TLR2*, *SA* and *MYD88* genes were investigated to determine which membrane receptor responds to WSPM stimulation in A549 cells. TLR4 mRNA level was found to be upregulated in the CeCl_3_, NaF and WSPM2.5 exposure groups with an increase of 27.28 times (*P* < 0.01), 8.73 times (*P* < 0.01) and 2.96 times (*P* < 0.05), respectively. Changes in *CMYD88* mRNA level were merely observed in the CeCl_3_ exposure group, with a decrease of 49.0 % (*P* < 0.05).TLR2 and SA were not detected due to low expression levels (results were not listed).

### Effects of WSPM exposure on the expression of caspase 3, 6, 8, and 9, and NF-κB in A549 cells

**Fig. 13:**
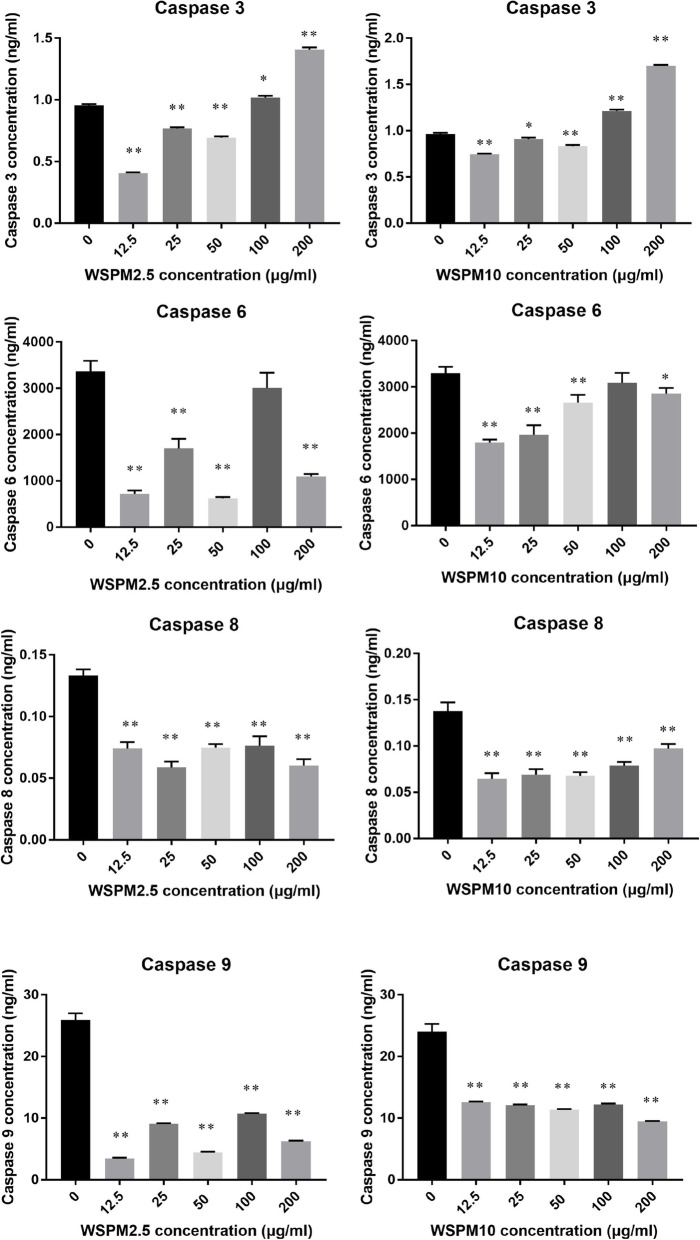
Effects of WSPM on the protein expression of caspase 3, 6, 8 and 9. The abscissa represents the exposure concentration of WSPM2.5 or WSPM10 (0, 12.5, 25, 50 and 200 µg/ml), while the ordinate represents the expression quantity of caspase 3, 6, 8 and 9. A549 cells were treated with WSPM2.5 or WSPM10 for 24 h, and the expression levels of caspase 3, 6, 8 and 9 were measured by ELISA (from top to bottom). *Compared with the solvent control group (0 µg/ml), *P* < 0.05; **Compared with the solvent control group (0 µg/ml), *P* < 0.01

In the cell cycle analysis from the above results, it was found that after the high-dose WSPM10 and WSPM2.5 treatment for A549 cells for 24 h, there was a sub-G1 peak, indicating that these cells may be apoptotic or necrotic. Hence, there is a need to understand the relationship of WSPM exposure and cell apoptosis. The intracellular expression quantity of caspase 3, 6, 8 and 9 were tested. It was found (Fig. 13) that the intracellular expression of caspase 3 was inhibited when the doses of WSPM10 or WSPM2.5 was < 100 µg/ml, and this significantly increased when the dose reached 100 µg/ml or higher (200 µg/ml) (*P* < 0.05). Caspase 8 and 9 were downregulated at all doses (*P* < 0.01). Caspase 6 expression was not statistically significant when the dose was 100 µg/ml, when compared to the control group (*P* > 0.05). When the dose was 200 µg/ml, the expression was downregulated. In addition, when the expression of NF-κB was analyzed, and it was found that WSPM exposure had no effect on the intracellular content of NF-κB (Figure S10).

## Discussion

The Baiyun Obo mining region is the most important light rare earth major producing areas in north China. The exploitation of resources has been going on for nearly a century, resulting in serious ecological pollution, rapid degradation of vegetation and worsening desertification [10]. The climate in this region is characterised by drought and low rainfall, and the dominant wind direction is northwest wind [10]. The chemical analysis results shown the distribution of rare earth elements in APM from this region is almost the same as that in soil [11, 12]. In addition, when the weather is windy, the ground particles in the mining area would enter the atmosphere in the form of sand dust. In Baotou city, the concentration of PM10 and PM2.5 were 0. 432 ± 0. 722 and 0. 206 ± 0. 254 mg /m^3^ in 2005, respectively, and has little changed from 2005 to 2006. In comparison with the non-sand-dust weather, the levels of PM10 and PM2.5 during sand-dust weather increased to some extent [12].

Some water-soluble ions, such as SO_4_^2−^, NO_3_^−^ and NH_4_^+^, are the common components of secondary particulate matter, and their proportion in PM_2.5_ is regional, such as Nanjing (45.1 % in living areas, 42.9 % in chemical areas, and 40.3 % in traffic areas), Xiamen (24.4 %), Tianjin (33.4 %) and Zhengzhou (66.1 %) [13]. In our results, water-soluble ions in the Bayan Obo mining area account for 26.48 and 37.18 % of PM_10_ and PM_2.5_, respectively, which is similar to the results of other studies. Ma et al. have shown that water soluble fraction constituted more than 39 % of the PM2.5 which were collected from 10 urban cities in China [14]. The water-soluble inorganic ions in PM10 have also a greater proportion. but its proportion is affected by dust weather [15]. The mass of water-soluble fraction showed a clear spatial variation in different areas. It accounted for 15.7–46.9 % of particle mass in the PM10 and 18.0-45.1 % of particle mass in the PM2.5, respectively [16].

WSPM2.5 has an obvious inhibitory effect on the viability of A549 cells. However, the inhibitory effect of WSPM10 was slightly different from WSPM2.5, and the cell viability inhibition is first increases and subsequently decreases with the increasing doses. This is somewhat different from the cytotoxicity of APM in other regions [17–21], which may be caused by differences in chemical composition of APM sample.

Compared with the controls, A549 cells were treated with WSPM10 or WSPM2.5 for 24 h. With the increasing doses, the percentage of cell in G2/M phase increased, and G1 phase cell fraction decreased. This resluts indicates that WSPM could block the A549 cell cycle to G2/M phase. G2/M arrest may be one of the reasons for the toxic mechanism of WSPM. Similar to the results in the present study, the G2/M arrest caused by APM exposure was also observed in other cells, including normal cells [22–25]. Longhin et al. found that Milan winter PM_2.5_ induced G2/M arrest in the BEAS-2B cell and augmented ROS formation. This effect is related to PM_2.5_ organic fraction, which cause damages to DNA [23]. However, WSPM in the Bayou Obo rare earth mining area which mainly contains inorganic component, can also induce A549 cell G2/M arrest and inhibit cell proliferation. In another study it was found that PM_2.5_ induced A549 cell cycle arrest in G2/M phase by upregulation of *p53* and *p21* and downregulation of *CDK1* mRNA level [26]. To explore the potential mechanisms of this effect, the proteome techniques [27] were used to evaluate the cytotoxic effects of WSPM in this study. According to DEPs molecular function, the proven DEPs were classified into 6 categories (Table S21), namely, metabolism-related enzymes, signal-transduction-related proteins, proteins associated with detoxification and transcription translation, ribosome related proteins, calcium binding proteins, and cellular structure-related proteins. The DEPs of WSPM10 and WSPM2.5 exposed groups involved both common components and their own characteristics. In general, ribosomal related proteins, had the highest degree of overlap in the two exposed groups, which included seven down-regulating RPs. In addition to protein synthesis, some RPs have non-protein synthesis functions (also known as extraribosomal functions [28]), including DNA transcription and repair [29], cell proliferation, cell cycle arrest, and apoptosis [30–32]. Furthermore, the expression of some RPs is inhibited without affecting cell protein synthesis [28, 33]. By interfering with the *RPL13*, *RPL6*, and *RPL18A* gene expression, cells exhibited a G1 phase arrest. Although this cell phenotype was different from WSPM10 and WSPM2.5 exposure groups. However, it also indicated that the expression of *RPL13*, *RPL6* and *RPL18A* genes are associated with cell cycle of A549 cells. The mRNA levels of *RPL13* and *RPL18A* in A549 cells were downregulated during WSPM exposure, which was consistent with previous identification results by mass spectrometry. This indicated that when the expression of *RPL13* and *RPL18A* is disrupted, the A549 cell cycle is affected. Although there have been no reported the regulatory mechanisms of *RPL13* and *RPL18A* on the cell cycle. Some RPs have been found to increase intracellular stability of p53 by direct interacting with MDM2, thereby affecting cell proliferation, cell cycle, and apoptosis, including RPL11 [34], RPL5 [35], RPL23 [36], RPS7 [37], RPS2 [38], RPL13 [39] and RPS25 [29]. This mechanism is also known as “nucleolar stress-MdM2-p53 signaling pathway “[40]. The exposure of Act D induced ribosomal stress. The p53 level sharply increased, and p21, a downstream gene of p53, shown the same increasing pattern as p53. We treated A549 cell with WSPM10 or WSPM2.5 for 24 h, the expression of ATM and p53 both increased in transcription and translation level. The mRNA level of MDM2 increased, and its protein level hardly changed as Act D exposure group. The protein level of p21 increased a small amount in the WSPM10 (100 µg/ml) or WSPM2.5 (200 µg/ml) exposure group, but its increase was not dose-dependent. The results show that WSPM blocked A549 cells in the G2/M phase by activating the ATM-p53-p21 signaling pathways, and this effect may be related to RPs. Liu et al. found that NaF induced cell cycle arrest by activating the ATM-p53-p21 on hepatocellular cell cycle progression in mice [41].

On the other hand, for the classical RB cell cycle regulation pathway, the activation of the cyclinD-CDK4/CDK6 kinase complex phosphorylates RB and promotes the release of E_2_F_1_ [42]. The protein level of CDK4, RB1, E_2_F_1_, and CDK 2 increased in the WSPM10 or WSPM2.5 exposure group. Among them, only the mRNA level of *RB1* significantly upregulated in both case of exposure, and the others upregulated in only one case of exposure. Compared with the NC group, when *RPL13* or *RPL18A* gene expression was inhibited, the mRNA level of *Rb1* significantly increased, while *E*_*2*_*F*_*1*_ mRNA expression significantly decreased. It may be due to Rb pathway activation, a large amount of E_2_F_1_ was released *in vivo*, and there was excessive intracellular E_2_F_1_, leading to the activation of a related negative feedback mechanism, inhibiting the expression of the *E*_*2*_*F*_*1*_ gene [43]. The mRNA level of *TP53* was no significant change in *RPL13*-knockdown cells and this increased by nearly 20 % in *RPL18A*-knockdown cells. There results indicate that G1 phase arrest may be induced through Rb/E_2_F_1_ pathway in *RPL13*-knockdown cells, rather than a p53-dependent manner. This arrest effect of G1 phase may be associated with both the p53 and Rb/E_2_F_1_ pathways in *RPL18A*-knockdown cells. There results shown that WSPM also induced A549 cell cycle arrest by activating Rb/E_2_F_1_ signaling pathways, and this effect also may be related to RPs.

WSPM chemical analysis revealed that La^3+^, Ce^3+^, Nd^3+^ and F^−^ were present in both WSPM2.5 and WSPM10. Furthermore, 2 mM NaF or CeCl_3_ induced A549 cells S phase arrest, but not significantly change (*P* > 0.05) in the LaCl_3_ or NdCl_3_ exposure group at the same doses. In addition, G2/M phase arrest was induced in the NaF exposed group, and this cell cycle phenotype was similar to that in the WSPM10 or WSPM2.5 exposure groups. Furthermore, this also suggests that LaCl_3_, NdCl_3_, NaF and CeCl_3_ may affect cell growth through different pathways. Fluorides have been shown to inhibit proliferation and induced apoptosis in epithelial lung cells from human and rats by activation of MAP kinase p38 and possibly JNK. In *in vitro* experiments on other cells, inhibition of protein synthesis and cell-cycle progression, alterations in cellular metabolism [3], and induction of inflammatory cytokine release [44] have been observed. Our results also showed that fluoride inhibited cell proliferation and cell cycle progression in A549 cells. In addition, fluoride is beneficial to cell proliferation by activation of ERK pathway in bone cells [44]. The activation of ERK by fluoride in the A549 cells is associated with the cell proliferation. This also suggests that fluoride may be both stimulatory proliferation signal and growth inhibitory signal in the lung cell [1]. CeCl_3_ inhibited human lung cancer cells PG cells (PG cells) proliferation and induced cell cycle arrest at the G1 phase. But it had no effect on human gastric carcinoma cells BGC823 and human diploid fibroblasts 2BS at the same concentration [45]. Lanthanum citrate has the same effect as CeCl_3_ for PG cells [46]. In addition, exposure to La (III) induced oxidative stress,activation of Ca^2+^-ATPase activity and inhibition of catalase, superoxide dismutase and glutathione peroxidase activity in the rats’ hippocampal cells [47]. The cytotoxicity of REE is influenced by the nature and concentration of REE, exposure time and cell types. It was found that mRNA expression changes of cell cycle regulators in the NaF and CeCl_3_ were highly consistent with those in the WSPM2.5 or WSPM10 exposure groups, including *MDM2, RB1, ATM, TP53, CDK2*, and *CDK4* genes. In the NaF exposure groups, the levels of transcription and translation of p53, MDM2, and p21 were inconsistent, but ATM, RB1 and E_2_F_1_ both were upregulated. It indicated that NaF may affect A549 cell proliferation and cell cycle by RB1-E_2_F_1_ pathway. Furthermore, the expression of p21, p53, E_2_F_1_, RB1 shown consistent translation level with transcription level in the LaCl_3_ and CeCl_3_ exposure groups. Besides RB1-E_2_F_1_ pathway, LaCl_3_ or CeCl_3_ may be induce A549 cells arrest by p53-MDM2-p21 pathway. Thus, it can be speculated that WSPM induced A549 cell cycle disorder through La^3+^, Ce^3+^, and F^−^. Different chemical components may be some differences in the mechanisms of action for A549 cell cycle [42]. Because of the difference in composition, WSPM2.5 and WSPM10 also differed with regard to the effects on the expression of cell cycle regulator.

In addition, The expression of *TLR4* upregulated in the CeCl_3_, NaF and WSPM2.5 exposure groups, and increased by 27.28 times (*P* < 0.01), 8.73 times (*P* < 0.01), and 2.96 times (*P* < 0.05), respectively, suggesting that TLR4 receptors may be play a role in the cell perception for these external stimuli [48].

The protein levels of caspase 3, 6, 8 and 9 were inhibited by low levels of WSPM10 or WSPM2.5 (<100 µg/ml), and exposed cells try to repair the mechanism to response with the external stimulation caused by the injury. Then, when the dose was more than 100 µg/ml, intracellular caspase 3 increased, which is the quantity of the cell debris flow diagram, indicating that cell apoptosis may have started the program. In addition, it was found that in the process of WSPM exposure, the expression of NF-κB has no significant change (*P* > 0.05), and the *MYD88* gene expression also did not exhibit obvious changes. Therefore, it can be concluded that the high dose of 200 µg/ml of WSPM, and not the NF-κB/MYD88 pathway, induced the apoptosis of A549 cells in the process. Furthermore, the expression of caspase 3 may be related to the increase in caspase 8 and 9, but there is no direct correlation.

Our study is the first to show a correlation between WSPM of Bayan Obo rare earth mining and A549 cell cycle, but some limitations should be considered. First, A549 cells is a widely used cell line in the field of cytotoxicity of atmospheric particulates, is not equivalent in sensitivity and response to human primary cell lines. However, A549 cells contained wild-type (*wt*) p53. Therefore, A549 cells has some value in the study of p53-mediated cell cycle regulation. Second, doses of WSPM are extremely high doses in our study, but in fact, WSPM is generally a small fraction of the total PM. It is not known whether there is a difference in the cell cycle effects between long-term low-dose and short-term high-dose exposure. Finally, the effect of cytotoxicity of F^−^, Ce^3+^ and La^3+^ on the A549 cells were studied in our study. A single component in WSPM can not completely represent toxic effect of WSPM. Some synergetic or antagonistic effects may exist between two or more components. Despite these limitations, the identification of the effects and potential mechanism between WSPM and cell cycle is a new step toward a better understanding of the correlation between atmospheric particulates and human health risks in Bayan Obo rare earth mining.

## Conclusions

In the process of investigating the toxicity mechanism of APM, many difficulties were encountered, and this was mainly because APM is a complex mixture of particles with complex physical and chemical properties, and its composition has certain regional and seasonal characteristics, making its toxicity mechanism often have some particularity. A toxicity analysis was conducted on the water-soluble fraction of APM in the Bayan Obo mining area. The toxic effects of WSPM on A549 cells is mainly manifested in inhibition of cell proliferation and ribosomal proteins biosynthesis, and DNA damage (Figure S11). In addition, La^3+^, Ce^3+^, and F^−^ were likely to be the main toxic substances in WSPM. Together with rare earth elements, such as Ce^3+^ and La^3+^, it affects the expression of *MDM2*, *RB1*, *ATM*, *TP53*, *p21* and *E*_*2*_*F*_*1*_ genes, thereby affecting the A549 cell cycle (Figure S12). However, many details still needs to be added through follow-up work. For example, determining whether there is any synergistic effect between different components in WSPM during this process, and determining how WSPM affects the reduction of ribosomal protein expression. The investigators consider that the answers to these questions can provide a scientific basis for the local response to atmospheric particulate pollution, and the protection of public health.

## Methods

### Collection and characterization of atmospheric particulates

The quartz fiber filters (90 mm Ø, 2 μm; Whatman, GE life science, UK) were employed for sampling by a middle volume sampler (Model TH-150, TianHong, Wuhan, China) at a flow rate of 100 L·min^− 1^. Different particle size cutters were used to collect atmospheric PM2.5 and PM10 samples respectively. The collection of every filter lasted for 24 h (from 8:00 a.m. to 8:00 a.m. the following day). Airborne PM10 and PM2.5 were collected from the residential area of the Bayan Obo mining district (41°47′N, 109°58′E), which is located at one kilometer south of the mine region in Baotou, an industrial city in northern China. 2 sampling points were set up in the mining area, 1000 m below the wind direction of the mining area. Each sampling point was equipped with 3 air samplers, one for PM_10_ collection, one for PM_2.5_ and the other for standby. When a failure happens on one sampler, then this standby sampler was able to continue sampling. For wind conditions, the prevailing wind direction was northwest, and the average wind speed was 3 m/s. The samplers were set on the rooftop, which was approximately 5.0 m above the ground from January to April in 2015.

After the sampling, these filters were carefully removed from the sampler, placed in a plastic container, and preserved in darkness at -4℃ until particle extraction or chemical characterization was performed.

### Particle extraction

After gravimetric analysis, The quartz fiber filter were cut into square pieces of 1.0 cm × 1.0 cm and placed in ultrapure water (Millipore, resistivity not less than 18.2 MΩ cm^− 1^ at 25 °C), which was fully mixed. Ultrasonic treatment was performed at room temperature (Shumei Instrument, KQ-700 V, Kunshan, China) for 30 min. Multilayer sterile gauze was used for filtration, and the insoluble substance was rinsed with ultrapure water for 3 times. The filtrate was mixed and prepared into powder by vacuum freeze-drying apparatus (Sihuan Scientific Instrument, LGJ-100 F, Beijing, China). The weights of the water-soluble fractions were obtained and its proportion in PM2.5 or PM10 were calculated. The detached particles were resuspended in sterilized normal saline to obtain aliquots at a final concentration of 2 mg/ml, and these were stored at -80℃ for use.

The chemical constituents of the samples were determined using inductively coupled plasma-atomic emission spectrometry (ICP-AES; SPECTRO ARCOSII GmbH Boschstr, Kleve, Germany) and ion chromatography (Dionex IC900; Thermo Fisher Scientific, USA) .

### Cell culture and WSPM treatment

Human lung epithelial cells A549 (Fuheng Biology Inc., Shanghai, China) were routinely maintained in RPMI-1640 medium at pH 7.2, supplemented with 10 % fetal bovine serum (FBS; Thermo Fisher Scientific, Gibco, USA) and 1 % penicillin/streptomycin (Thermo Fisher Scientific, Gibco, USA), and maintained in a humidified 5 % CO_2_ incubator at 37 °C. A549 cells were cultured for 24 h in 96-well plates at a density of 5 × 10^4^ cells per well. The working solutions of WSPM were prepared by diluting the stock solution in culture medium. A549 cells were treated with WSPM at the final concentration of 0, 12.5, 25, 50, 100 or 200 µg/ml for 6, 24, 48 or 72 h, respectively, or treated with actinomycin D (5 nM) for 24 h. Meanwhile, the normal culture medium was set as the control group.

### MTT assay

After treatment with WSPM, these cells were washed and treated with 20 µl of 5 mg/ml of 3-(4,5-dimethylthiazol-2-yl)-2,5-diphenyl tetrazolium bromide (MTT, Sigma-Aldrich, MO, USA) to determine the cell viability. After formazan formation by MTT, 150 µl of dimethyl sulfoxide (DMSO; Sigma-Aldrich, MO, USA) was added with oscillation for 10 min. Optical density (OD) was measured at 490 nm using a microplate reader (Thermo Scientific, PA, USA). Cell inhibitory rate = [1- (OD experiments – OD blank) / (OD control - OD blank)] × 100 %. The experiments for control and exposure groups were each performed in triplicate.

### Flow cytometric cell cycle

Cells were treated with WSPM10 after 10^6^ cells were placed in 6-well plates at 37 °C with 5 % CO_2_ for 24 h. Cells were detached by trypsinization, and collected and fixed in 5 ml of 75 % cold ethanol at 4 °C for two hours. Then, these cells were incubated with 0.5 ml of propidium iodide (PI; Sigma Aldrich, MO, USA) at 37 °C for 30 min. DNA content was detected using a flow cytometer (ACEA, NovoCyte, USA).

### Protein extraction and digestion

After treatment, the A549 cells were washed for three times with cold PBS, and scraped and centrifuged at 1,500 g for three minutes. Then, the resulting pellets were homogenized in 0.55 ml of lysis buffer (30 mM of HEPES, 8 M of urea, 1 mM of PMSF, 2 mM of EDTA and 10 mM of DTT), vortexed for one minute, sonicated for five minutes, and unlysed debris by centrifugation at 20,000 g for 30 min. Afterwards, the protein was reduced (10 mM of DTT, 56 °C, one hour), alkyated (55 mM of iodoacetamide, 37 °C, two hours) and precipitated with the addition of 4 volumes of cold acetone (-20 °C, three hours). Next, the protein was collected by centrifugation (20,000 g, 4 °C, 30 min), and the precipitates were redissolved in 0.30 ml of buffer (50 % TEAB, 0.1 % SDS), sonicated [49] for three minutes, centrifuged (20,000 g, 4 °C, 30 min), and frozen at -80 °C. The protein concentration of the supernatant was measured using the Bradford assay. For each sample, 100 µg of protein was resuspended in digestion buffer (50 % TEAB, 0.1 % w/v SDS). Then, equal aliquots of 100 µg from each lysate were digested with 3 µl of 1 µg/µl of trypsin for 24 h at 37 °C, and lyophilized. Afterwards, 30 µl of 50 % TEAB was added into each sample tube.

### Labeling with the iTRAQ 8-plex reagent

Labeling solutions were provided by the iTRAQ 8-plex reagent kits (Applied Biosystem, Carlsbad, CA, USA). According to manufacturer protocol, the fresh vial of iTRAQ reagent 8-plex was required to reach room temperature. Then, 70 µl of isopropanol were added to each reagent vial, and vortex-mixed for one minute. Afterwards, the peptides were labeled with the iTRAQ label reagent, and 8-plex labeling was performed for two hours at room temperature. All labeled peptides, in which the quantity was determined from each tag, were combined in one tube. Finally, the pooled peptides were dried under vacuum [50–52].

### Cation exchange chromatography

The combined peptide mixture was separated using a strong cation exchange column (Phenomenex, Luna ®SCX, 4.6 × 250 mm, 5 μm, 100 Å; Torrance, California, USA). The labeled peptides were solubilized with 1 ml of loading buffer (25 % v/v acetonitrile, 10 mM of KH_2_PO_4_, pH 3.0 with phosphoric acid) and centrifuged (15,000 g, 4 °C, 10 min). Then, the supernatant was loaded and washed isocratically for 40 min at 0.5 ml/min to remove the excess reagent. The samples were eluted with a gradient of 0-2 M KCl (25 % v/v acetonitrile, 10 mM of KH_2_PO_4_, pH 3.0 with phosphoric acid) over 36 min at 1.0 ml/min, with fractions collected at one minute intervals.

### Peptide desalination

Individual SCX fractions were desalted using solid-phase extraction cartridges (Phenomenex Strata-X cartridge, 3 ml and 60 mg; Torrance, California, USA). The Strata-X cartridge were washed with 1 ml of methanol and 1 ml of water prior to use, and were equilibrated with 5 % acetonitrile. Then, the sample was solubilized in 1 ml of ultrapure water, loaded onto the Strata-X cartridge, and desalted with 1 ml of 5 % acetonitrile. The peptides were eluted using 1 ml of acetonitrile from the column, and the solvent was removed by vacuum drier under 4 °C.

### LC-MS analysis

The desalted peptide mixture was redissolved with 0.1 % formic acid (FA). Peptide separation was performed on a nano-chromatography system (Ultimate™ 3000, Thermo Fisher Scientific, USA). The sample was injected and captured onto a C18 column (Acclaim™ PepMap™, 75 μm × 2 cm, 3 μm, 100 Ǻ; Thermo Fisher Scientific, USA) and eluted onto a C18 analytical column (75 μm × 10 cm, 5 μm, 300 Ǻ, Agela Technologies, China). The peptides were eluted using an automated gradient from 95 % (v/v) buffer A (0.1 % FA in water) to 80 % (v/v) buffer B (0.1 % FA in acetonitrile) over 48 min at a flow rate of 400 nl·min^− 1^.

The eluted peptides from the C18 column were directly entered to the Q-Exactive MS (Thermo Fisher Scientific, Waltham, MA, USA) through a capillary tip for electrospray, which was set in positive ion mode. The electrospray voltage was 1.8 kV, and the capillary temperature was 320 °C. For each run, 1 µl of sample was loaded, and each sample was analyzed in duplicate. Full MS scans were acquired in the Orbitrap mass analyzer within 350–2000 m/z, with a full scan resolution of 70,000 (m/z 200) and an MS/MS scan resolution of 17,500 (m/z 200). The MS/MS scan had a minimum signal threshold of 1 × 10^5^, and an isolation width of 2 Da. In order to evaluate the performance of the mass spectrometry on the iTRAQ labeled samples, higher collision energy dissociation (HCD) was employed. In order to optimize the MS/MS acquisition efficiency of HCD, normalized collision energy (NCE) was systemically examined at 28, stepped 20 %. The MS survey spectrum was measured.

### Peptide and protein identification

All .raw files were converted to MGF format using Proteome Discoverer 1.3/1.4 (Thermo Fisher Scientific, Waltham, MA). MASCOT 2.3.01 (Matrix Science, Boston, MA, USA) was used for the database search against the Uniprot human database (updated on 15/03/2016, 20,199 sequences). The corresponding reversed sequences were also appended to the database for estimating the false discovery rate (FDR) of the peptide identification. The database search parameters included up to one missed cleavages allowed for full tryptic digestion, a mass tolerance of 15 ppm for the precursor, and a mass tolerance of 20 mmu for fragment ions. For all experiments, the carbamidomethylation of cysteine residues was set as a fixed modification, while methionine oxidation, and N-terminal acetylation of protein and iTRAQ modification of peptide N termini were set as variable modifications.

The result from each run indicated less than 1 % FDR for peptide identification. For identifications where multiple peptides met these criteria, the results indicated a less than 0.1 % FDR. On the protein level, there was a minimum number of peptide 1 for each protein, in which only rank 1 peptides were counted, and peptides only counted in top scored proteins were applied for all data filtration. In addition, protein grouping was enabled, and a strict maximum parsimony principle was applied. Therefore, if multiple proteins were identified from same peptides, these proteins were grouped into one protein group, and each protein group has at least one unique peptide. The protein groups and peptides identified by UHPLC ESI-MS/MS analysis from A549 cell lysate digest with the MASCOT database search are listed in the supporting material.

The protein quantification settings were as follows: protein ratio type of ‘median’, normalization method of the ‘median’, outlier removal of ‘automatic’, minimum peptide threshold of ‘1’, a 95 % confidence interval (*P* < 0.05) to assess the accuracy of the protein ratio, and a minimum of three spectra for quantifying a protein. The iTRAQ ratios were normalized to the control group.

### Bioinformatics analysis of proteomics data

The identified proteins were classified according to the annotations obtained from the UniProt knowledgebase. OmicsBean (http://www.omicsbean.cn) was utilized to analyze the GO distribution of the obtained differential proteins. The distributions of each protein in the biological process (BP), cellular components (CCs) and molecular functions (MF) were based on the Gene Ontology (GO) categories. A Kyoto Encyclopedia of Genes and Genomes (KEGG) pathway analysis (http://www.genome.ad.jpkegg/pathway.html) was performed to enrich the high-level functions in the defined biological systems.

### Verification of DEPs

#### Multiple reaction monitoring (MRM) analysis

For each differentially expressed protein in the iTRAQ data, one unique tryptic peptide of length of 6–20 amino acids were chosen for the MRM. Peptides were excluded when they had a missed cleavage, had potential ragged ends, or contained amino acids that were susceptible to variable modifications, including cysteine and methionine. Precursor ions were monitored for confident identification of the peptides. Candidates for control proteins were identified from iTRAQ, according to the following criteria: if they had a fold change of 1 ± 0.2, the coefficient of variation (CV) was < 10 % in the iTRAQ data. Next, the samples were trypsin digested. For the supernatant samples, strong cation exchange was performed using SCX tips (Thermo Fisher Scientific) after digestion. The MRM assays were carried out using a QTRAP 6500 mass spectrometer (AB SCIEX, CA, USA) coupled to an ekspert NanoLC 425 system (Ekspert Technologies, USA). The mobile phases consisted of solvent A, 2 % acetonitrile with 0.1 % FA and solvent B, and 98 % acetonitrile with 0.1 % FA. Each sample was loaded onto a NanoLC trap column (ChromXP 18CL, 350 μm × 0.5 mm, 3 μm, 120 Å; Ekspert Technologies, USA). The peptides were separated using a NanoLC column (ChromXP C18, 75 μm × 15 cm, 5 μm, 120 Å, Ekspert Technologies, USA) at 0.3 µl·min^− 1^, with a linear gradient of 5–45 % solvent B for 48 min and 80 % solvent B for five minutes. The MS parameters were set, as follows: positive ionization, high sensitivity mode, ion source gas 1 (GS1) at 15.00, ion source gas 2 (GS2) at 0.00, curtain gas (CUR) at 35.00, ionspray voltage floating (ISVF) at 2,400.00 V, interface heater temperature (IHT) at 150.00 °C, Q1 and Q3 at unit resolution, scan speed at 10 Da/s, collision gas (CAD) at high, entrance potential (EP) at 10, and collision cell exit potential (CXP) at 10.

#### Western blot analysis

Cells were collected in RIPA lysis buffer, and the proteins were quantified using a BCA Protein Concentration Assay Kit (MDL Biotechnology, China). The protein was isolated on a 10 % SDS-PAGE gel (MDL Biotechnology, China), and transferred onto PVDF membranes. Then, the membranes were blocked with 5 % (w/v) nonfat dry milk in PBS and 0.05 % Tween. The primary antibodies used as follows: anti-FAK antibody (1:1,000; MDL Biotechnology, China), anti-CDC27 antibody (1:1,000; MDL Biotechnology, China), RPRD1A-antibody (1:1,000; MDL Biotechnology, China), RPL34-antibody (1:1,000; MDL Biotechnology, China), RPL24-antibody (1:1,000; MDL Biotechnology, China), EIF6-antibody (1:1,000; MDL Biotechnology, China), p53-antibody (1:1000; Affinity, USA), MDM2-antibody (1:1000; Abcam, USA), p21-antibody (1:1000; Epitmics, USA), CDK2-antibody (1:3000; Abcam, USA), CDK4-antibody (1:3000; Abcam, USA), RB-antibody (1:2000; Abcam, USA), ATM-antibody (1:3000; Abcam, USA), E_2_F_1_-antibody (1:1000; Abcam, USA). β-actin (1:1000; MDL Biotechnology, China) or GAPDH (1:5000; Abcam, USA) was used as the loading control. The secondary antibodies were incubated with goat IgG-HRP (1:5,000; MDL Biotechnology, China) at room temperature for one hour. The blots were detected using a ChemiDoc MP chemiluminescence imaging system (Bio-rad, USA).

#### Small interfering RNA

Based on the gene sequence in GenBank and siRNA design principles, the siRNA sequences were designed and chemically synthesized by Shanghai Shenggong Biotech. The constructs are listed in Table S1. The cell blank, negative control and interference groups were set-up. Cells in the exponential growth period were inoculated into the cell culture plate at a certain concentration for 24 h, and the transfection cell density reached 50–70 %. The siRNA oligonucleotides were transfected into A549 cells using the lipofectamine 3000 (Thermo Fisher, USA), according to manufacturer’s instructions. The transfection efficiency was detected by fluorescent labeled Cy3-siRNA.

#### RNA isolation and quantitative real-time PCR assay

A549 cells were seeded in 6-well plates at a density of approximately 1 × 10^6^ cells per well. Then, these cells were exposed to WSPM, LaCl_3_, CeCl_3_, NdCl_3_ and NaF, respectively, for 24 h. Subsequently, these cells were trypsinized and collected. Afterwards, the adherent cells were collected. The total RNA was extracted using the TRIzol reagent (Invitrogen, USA), according to manufacturer’s instructions.

The total RNA of A549 cells was extracted using a TRIzol™ RNA reagent (InvitroGen, USA), according to manufacturer’s protocol. The mRNA levels for modulated genes were determined by the reverse transcription of total RNA, followed by qRT-PCR, on a LightCycler 480 real-time PCR (Roche, Germany) using PrimeScript reverse transcription reagent kits (Takara, Japan), according to manufacturer’s protocol. The primers were designed for the modulated genes. These are presented in Supplemental Table S2. All experiments were performed in triplicate.

#### Enzyme-linked immunosorbent assay (ELISA)

The levels of related cytokines in A549 cells were measured using the Human Caspase 3, 6, 8 and 9 ELISA Kit (MDL Sciences, China), according to manufacturer’s instructions. Each sample was assayed in triplicate.

### Statistical analysis

The data (except for the fold change of protein expression) were all expressed as the mean ± standard deviation (SD) of triplicate experiments. Significant differences among multiple groups were determined using one-way analysis of variance (ANOVA), followed by the least significant difference post hoc test. Probabilities of *P* < 0.05 were considered statistically significant.

## Supplementary Information

**Additional file 1:**

## Data Availability

The datasets used and/or analysed during the current study are available from the corresponding author on reasonable request.

## Abbreviations

WSPM10: Water soluble particulate matter 10
WSPM2.5: Water soluble particulate matter 2.5
WSPM: The general term for WSPM10 and WSPM2.5
APM: Atmospheric particulate matter
REE: Rare earth elements
iTRAQ: Isobaric tags for relative and absolute quantification
IC: Ion Chromatography
ICP-AES: Inductively Coupled Plasma-Atomic Emission Spectroscopy
qRT-PCR: Quantitative real time polymerase chain reaction
MRM: Multiple reaction monitoring
MTT: 3-(4,5-dimethyl-2-thiazolyl)-2,5-diphenyl-2-H-tetrazolium bromide
DMSO: Dimethyl sulfoxide
ELISA: Enzyme linked immunosorbent assay
RPL: 60S ribosomal protein
RPS: 40S ribosomal protein
TLRs: Toll-like receptors
GO: Gene Ontology
KEGG: Kyoto Encyclopedia of Genes and Genomes
NC: Negative control
LPS: Lipopolysaccharides
DEP: Diesel Exhaust Particles
LDH: Lactate dehydrogenase
OD: Optical density
PI: Propidium iodide
FC: Fold Change
FDR: False discovery rate
LC-MS/MS: Liquid chromatography-tandem mass spectrometry
siRNA: Small interfering RNA
WB: Western blot
SDS-PAGE: Sodium dodecyl sulfate-polyacrylamide gel electrophoresis
