# Supplementary material for: Effects of water-soluble components of atmospheric particulates from rare earth mining areas in China on lung cancer cell cycle
Source: Part Fibre Toxicol. 2021 Aug 2;18:27. doi: 10.1186/s12989-021-00416-z (PMC8330054; doi:10.1186/s12989-021-00416-z)
Supplement: Supplementary file 1 — Additional file 1: [file 12989_2021_416_MOESM1_ESM.docx]

Supplementary Material

Table of Contents

**Table S1.** Primer list of siRNA.

**Table S2.** Primer list of real-time quantitative PCR.

**Table S3.** The differentially expressed proteins were verified in WSPM10 exposed group by MRM method.

**Table S4.** The differentially expressed proteins were verified in WSPM2.5 exposed group by MRM method.

**Table S5.** The differentially expressed proteins were verified by Western blot.

**Figure S1.** The effect of WSPM10 on cell cycle for A549 cell. A549 cells were treated with concentrations of 0 μg/ml (A), 12.5 μg/ml (B), 50 μg/ml (C) and 200 μg/ml (D) WSPM10 for 24 h. After incubation with PI for 30min, cell cycle was analyzed by flow cytometry.

**Figure S2.** The effect of WSPM2.5 on cell cycle for A549 cell. A549 cells were treated with 0 μg/ml (A), 12.5 μg/ml (B), 50 μg/ml (C) and 200 μg/ml (D) WSPM2.5 for 24 h. After incubation with PI for 30min, cell cycle was analyzed by flow cytometry.

**Figure S3.** Volcano plot of differential expression proteins of WSPM10 group. The distribution of differentially expressed proteins in A549 cells which were treated with different concentrations of WSPM10 for 24 h. The red dots indicated upregulated of protein expression and the green dots indicated downregulated of protein expression. R-code was used to screen the differentially expressed proteins from the mass spectrometry results, and the ggplot2 package was used to draw the volcano map.The abscissais the logarithm of FC, and the ordinate is the inverse of the logarithm of p value.

**Figure S4.** Volcano plot of differential expression proteins of WSPM2.5 group. The distribution of differentially expressed proteins in A549 cells which were treated with different concentrations of WSPM2.5 for 24 h. The red dots indicated upregulated of protein expression and the green dots indicated downregulated of protein expression. R-code was used to screen the differentially expressed proteins from the mass spectrometry results, and the ggplot2 package was used to draw the volcano map.The abscissais the logarithm of FC, and the ordinate is the inverse of the logarithm of *p* value.

**Figure S5.** GO enrichment analysis of group treated with 12.5 μg/ml (a), 50 μg/ml (b), 200 μg/ml (c) of WSPM10. The cutoff of *p*-value was set to 0.05. Terms in the same category were ordered based on the *p*-values.

**Figure S6.** GO enrichment analysis of group treated with 12.5 μg/ml (a), 50 μg/ml (b), 200 μg/ml (c) of WSPM2.5. The cutoff of *p*-value was set to 0.05. Terms in the same category were ordered based on the *p*-values.

**Figure S7** The evaluation of RNA interference effect. The expression levels of *RPL6*、*RPL18A*、*RPL13* and *HisTIH4A* genes were measured by qRT-PCR at 24 h after siRNA transfection, respectively.

**Figure S8.** The effect of *RPL13, RPL6, RPL18A or HIS1H4A* gene interference on cell cycle. The A549 cells were incubated with PI for 30 min, then cell cycle was detected by flow cytometry 48 h after siRNA transfection. (A) blank control group; (B) negative control group;(C) *RPL13* interference group; (D) *RPL6* interference group; (E) *RPL18A* interference group; (F) *HIS1H4A* interference group.

**Table S6.** The effect of gene interference on cell cycle.

**Table S7.** Effects of LaCl_3_, CeCl_3_, NdCl_3_ and NaF on the proliferation inhibition rate of A549 cells

**Figure S9.** Effects of LaCl_3_, CeCl_3_, NdCl_3_ and NaF on A549 cell cycle after exposure for 24 h. The solvent blank was used as control group（A）.Under the same concentration（2 mM）, A549 cells were treated with LaCl_3_ (B), CeCl_3_ (C), NdCl_3_（D）and NaF（E）for 24 h，respectively. After incubation with PI for 30 min, cell cycle was analyzed by flow cytometry.

**Table S8.** Effects of LaCl_3_, CeCl_3_, NdCl_3_ and NaF on A549 cell cycle.

**Figure S10.** Effects of WSPM on the protein expression of NF-κB. The abscissa is the exposure concentration of WSPM10 or WSPM2.5, and the ordinate is the expression of NF-κB. (A) and (B) were the NF-κB expression of A549 cells treated with different concentration (0, 12.5, 25, 50 and 200 μg/ml) of WSPM10 and WSPM2.5 for 24 h.* compared with the solvent control group (0 μg/ml), *p* < 0.05; ** compared with the solvent control group (0 μg/ml), *p* < 0.01.

**Figure S11.** A549 cells were treated with WSPM10 (50 μg/ml) and WSPM2.5 (12.5, 50 μg/ml and 200 μg/ml) for 24 hours. Cell lysates were immunoblotted with the indicated antibodies. A549 cells treated with either DMSO was used as negative control (NC).

**Figure S12.** Diagram of potential toxicity mechanism of WSPM for A549 cells. ‘↑’ is the upregulated expression of protein; ‘↓’ is the downregulated expression of protein. Each relationship is shown with a solid blue arrow. The broken lines with arrows represent may be a potential connections.

**Figure S13.** The influence of WSPM on A549 cell cycle and its potential mechanism. ‘↑’ is the upregulated expression of protein; ‘↓’ is the downregulated expression of protein. Each relationship is shown with a solid blue arrow. The broken lines with arrows represent may be a potential connections.

**Table S9.** Significance enrichment analysis of differentially expressed proteins involved in biological processes in 12.5 μg/ml WSPM10 group.

**Table S10.** Significance enrichment analysis of differentially expressed proteins involved in biological processes in 50 μg/ml WSPM10 group.

**Table S11.** Significance enrichment analysis of differentially expressed proteins involved in biological processes in 200 μg/ml WSPM10 group.

**Table S12.** Significance enrichment analysis of differentially expressed proteins involved in biological processes in 12.5 μg/ml WSPM2.5 group.

**Table S13**. Significance enrichment analysis of differentially expressed proteins involved in biological processes in 50 μg/ml WSPM2.5 group.

**Table S14.** Significance enrichment analysis of differentially expressed proteins involved in biological processes in 200 μg/ml WSPM2.5 group.

**Table S15.** Significant pathway enrichment of differentially expressed proteins in the 12.5 μg/ml WSPM10 group.

**Table S16.** Significant pathway enrichment of differentially expressed proteins in the 50 μg/ml WSPM10 group.

**Table S17.** Significant pathway enrichment of differentially expressed proteins in the 200 μg/ml WSPM10 group.

**Table S18.** Significant pathway enrichment of differentially expressed proteins in the 12.5 μg/ml WSPM2.5 group.

**Table S19.** Significant pathway enrichment of differentially expressed proteins in the 50 μg/ml WSPM2.5 group.

**Table S20.** Significant pathway enrichment of differentially expressed proteins in the 200 μg/ml WSPM2.5 group.

**Table S21.** Functional classification of verified differentially expressed proteins.

**Table S22.** Expression of Several Cell cycle regulators in A549 Cells Exposed to the WSPM, NaF, CeCl_3_ or LaCl*_3_.*

| **Table S1. Primer list of siRNA.** | | |
| --- | --- | --- |
| siRNA name | Sequences（5’→3’） | |
| Hs-HIST1H4A-si-1 | Sense | GCAAGGUGUUGCGUGACAAdTdT |
|  | Antisense | UUGUCACGCAACACCUUGCdTdT |
| Hs-HIST1H4A-si-2 | Sense | GGAUCUCUGGUCUGAUCUAdTdT |
|  | Antisense | UAGAUCAGACCAGAGAUCCdTdT |
| Hs-HIST1H4A-si-3 | Sense | GGUCUGAUCUACGAGGAGAdTdT |
|  | Antisense | UCUCCUCGUAGAUCAGACCdTdT |
| Hs-RPL6-si-1 | Sense | GGGUAACCUCAAAGCUAAAdTdT |
|  | Antisense | UUUAGCUUUGAGGUUACCCdTdT |
| Hs-RPL6-si-2 | Sense | GCGCAAGAUUGAUCAGAAAdTdT |
|  | Antisense | UUUCUGAUCAAUCUUGCGCdTdT |
| Hs-RPL6-si-3 | Sense | CAGAUACUAAAGAGAAGAAdTdT |
|  | Antisense | UUCUUCUCUUUAGUAUCUGdTdT |
| Hs-RPL13-si-1 | Sense | GGAAGAGAAGAAUUUCAAAdTdT |
|  | Antisense | UUUGAAAUUCUUCUCUUCCdTdT |
| Hs-RPL13-si-2 | Sense | CGCAGAACAGGAUGUUGAAdTdT |
|  | Antisense | UUCAACAUCCUGUUCUGCGdTdT |
| Hs-RPL13-si-3 | Sense | CGUAAGAUCCGCAGACGUAdTdT |
|  | Antisense | UACGUCUGCGGAUCUUACGdTdT |
| Hs-RPL18A-si-1 | Sense | GCUACGAGAGUACAAGGUAdTdT |
|  | Antisense | UACCUUGUACUCUCGUAGCdTdT |
| Hs-RPL18A-si-2 | Sense | GUACUUUGUAUCUCAGUUAdTdT |
|  | Antisense | UAACUGAGAUACAAAGUACdTdT |
| Hs-RPL18A-si-3 | Sense | GCGCUGUCACCCAGUGCUAdTdT |
|  | Antisense | UAGCACUGGGUGACAGCGCdTdT |

| **Table S2. Primer list of real-time quantitative PCR.** | | |
| --- | --- | --- |
| siRNA name | Sequences（5’→3’） | |
| hHIST1H4A | Sense | AAGGGTTTGGGTAAGGGGG |
|  | Antisense | TAGATCAGACCAGAGATCCGC |
| hRPL6 | Sense | CGGGTGGTTAAACTTCGCAAA |
|  | Antisense | CCAGTCACAAGTAATAAGCCACT |
| hRPL13 | Sense | TCAAAGCCTTCGCTAGTCTCC |
|  | Antisense | GGCTCTTTTTGCCCGTATGC |
| hRPL18A | Sense | GGCACGCTACGAGAGTACAAG |
|  | Antisense | ATTAGGCGCAAAGATTCGCAT |
| ATM | Sense | ATCTGCTGCCGTCAACTAGAA |
|  | Antisense | GATCTCGAATCAGGCGCTTAAA |
| CCND1 | Sense | GCTGCGAAGTGGAAACCATC |
|  | Antisense | CCTCCTTCTGCACACATTTGAA |
| CCNA2 | Sense | CGCTGGCGGTACTGAAGTC |
|  | Antisense | GAGGAACGGTGACATGCTCAT |
| CCNB1 | Sense | AATAAGGCGAAGATCAACATGGC |
|  | Antisense | TTTGTTACCAATGTCCCCAAGAG |
| CCNE1 | Sense | AAGGAGCGGGACACCATGA |
|  | Antisense | ACGGTCACGTTTGCCTTCC |
| CDC25A | Sense | GTGAAGGCGCTATTTGGCG |
|  | Antisense | TGGTTGCTCATAATCACTGCC |
| CDK2 | Sense | CCAGGAGTTACTTCTATGCCTGA |
|  | Antisense | TTCATCCAGGGGAGGTACAAC |
| CDK4 | Sense | ATGGCTACCTCTCGATATGAGC |
|  | Antisense | CATTGGGGACTCTCACACTCT |
| CDKN1A | Sense | TGTCCGTCAGAACCCATGC |
|  | Antisense | AAAGTCGAAGTTCCATCGCTC |
| CDKN2A | Sense | ATGGAGCCTTCGGCTGACT |
|  | Antisense | GTAACTATTCGGTGCGTTGGG |
| CHEK2 | Sense | TCTCGGGAGTCGGATGTTGAG |
|  | Antisense | CCTGAGTGGACACTGTCTCTAA |
| E2F1 | Sense | ACGTGACGTGTCAGGACCT |
|  | Antisense | GATCGGGCCTTGTTTGCTCTT |
| MDM2 | Sense | GAATCATCGGACTCAGGTACATC |
|  | Antisense | TCTGTCTCACTAATTGCTCTCCT |
| RB1 | Sense | CTCTCGTCAGGCTTGAGTTTG |
|  | Antisense | GACATCTCATCTAGGTCAACTGC |
| S100A4 | Sense | GATGAGCAACTTGGACAGCAA |
|  | Antisense | CTGGGCTGCTTATCTGGGAAG |
| TP53 | Sense | ACAGCTTTGAGGTGCGTGTTT |
|  | Antisense | CCCTTTCTTGCGGAGATTCTCT |
| hACTB | Sense | TCGTGCGTGACATTAAGGAG |
|  | Antisense | GTCAGGCAGCTCGTAGCTCT |
| TLR4 | Sense | GGTCAGACGGTGATAGCGAG |
|  | Antisense | GGATTTCACACCTCCACGCA |
| RPL13 | Sense | TCTTCTTCGCAGACAGCGTT |
|  | Antisense | GCCTTACGTCTGCGGATCTT |
| IRAK4 | Sense | TCCTAGTTCGGCTGGTTCTTC |
|  | Antisense | AGTTTTGGGAACAGCATCTGCC |
| MyD88 | Sense | TCACTGAGGGAGCCTAACCA |
|  | Antisense | CTGGGGCACAGACACCTAAG |

| **Table S3. The differentially expressed proteins were verified in WSPM10 exposed group by MRM method.** | | | | |
| --- | --- | --- | --- | --- |
|  | Gene name | Protein name | iTRAQ vs. control | MRM vs.  control |
| 12.5 μg/ml  WSPM10 | NSDHL | Sterol-4-alpha-carboxylate 3-dehydrogenase, decarboxylating | 0.716 | 0.827 |
|  | SCD | Acyl-CoA desaturase | 0.815 | 0.779 |
|  | CSTF2 | Cleavage stimulation factor subunit 2 | 1.21 | 1.44 |
|  | MIF | Macrophage migration inhibitory factor | 1.28 | 2.35 |
|  | MT2A | Metallothionein-2 | 1.29 | 1.82 |
| 50 μg/ml  WSPM10 | RPL18A | 60S ribosomal protein L18a | 0.798 | 0.622 |
|  | RPL14 | 60S ribosomal protein L14 | 0.777 | 0.741 |
|  | EIF3I | Eukaryotic translation initiation factor 3 subunit I | 1.21 | 1.88 |
|  | HBA1 | Hemoglobin subunit alpha | 1.60 | 4.75 |
|  | MRPS7 | 28S ribosomal protein S7, mitochondrial | 1.22 | 1.74 |
|  | ASNA1 | ATPase ASNA1 | 1.24 | 2.82 |
|  | KRT10 | Keratin, type I cytoskeletal 10 | 1.34 | 1.63 |
|  | MT2A | Metallothionein-2 | 1.393 | 2.99 |
| 200 μg/ml  WSPM10 | RPL38 | 60S ribosomal protein L38 | 0.747 | 0.576 |
|  | RPL18A | 60S ribosomal protein L18a | 0.757 | 0.3183 |
|  | RPL27 | 60S ribosomal protein L27 | 0.712 | 0.359 |
|  | RPL6 | 60S ribosomal protein L6 | 0.737 | 0.413 |
|  | RPL27A | 60S ribosomal protein L27a | 0.696 | 0.396 |
|  | RPS13 | 40S ribosomal protein S13 | 0.807 | 0.602 |
|  | RPS2 | 40S ribosomal protein S2 | 0.745 | 0.457 |
|  | RPS25 | 40S ribosomal protein S25 | 0.797 | 0.507 |
|  | RPL8 | 60S ribosomal protein L8 | 0.822 | 0.639 |
|  | FBL | rRNA 2'-O-methyltransferase fibrillarin | 0.726 | 0.665 |
|  | RPL19 | 60S ribosomal protein L19 | 0.821 | 0.330 |
|  | PSMF1 | Proteasome inhibitor PI31 subunit | 0.816 | 0.505 |
|  | CNN3 | Calponin-3 | 0.827 | 0.536 |
|  | RPL36 | 60S ribosomal protein L36 | 0.808 | 0.577 |
|  | RPL22L1 | 60S ribosomal protein L22-like 1 | 0.802 | 0.544 |
|  | COPS6 | COP9 signalosome complex subunit 6 | 1.22 | 2.06 |
|  | CLEC3B | Tetranectin | 1.75 | 20.1 |
|  | HBA1 | Hemoglobin subunit alpha | 3.13 | 5.83 |

| **Table S4. The differentially expressed proteins were verified in WSPM2.5 exposed group by MRM method.** | | | | |
| --- | --- | --- | --- | --- |
|  | Gene name | Protein name | iTRAQ vs. control | MRM vs.  control |
| 12.5 μg/ml  WSPM2.5 | RPL38 | 60S ribosomal protein L38 | 0.500 | 0.834 |
|  | HIST1H4A | Histone H4 | 0.668 | 0.274 |
|  | ACBD3 | Golgi resident protein GCP60 | 0.770 | 0.379 |
|  | PXN | Paxillin | 0.771 | 0.730 |
|  | ERP44 | Endoplasmic reticulum resident protein 44 | 0.787 | 0.576 |
|  | PSMB6 | Proteasome subunit beta type-6 | 0.800 | 0.497 |
|  | MT2A | Metallothionein-2 | 1.37 | 1.22 |
| 50 μg/ml  WSPM2.5 | HIST1H4A | Histone H4 | 0.568 | 0.779 |
|  | RPL18A | 60S ribosomal protein L18a | 0.717 | 0.362 |
|  | RPL13 | 60S ribosomal protein L13 | 0.708 | 0.231 |
|  | CSTF2 | Cleavage stimulation factor subunit 2 | 1.21 | 1.46 |
|  | ABHD10 | Mycophenolic acid acyl-glucuronide esterase, mitochondrial | 1.34 | 1.94 |
|  | TIMM13 | Mitochondrial import inner membrane translocase subunit Tim13 | 1.25 | 3.61 |
|  | SLC7A5 | Large neutral amino acids transporter small subunit 1 | 1.21 | 2.61 |
|  | CYP1B1 | Cytochrome P450 1B1 | 1.30 | 7.08 |
|  | MT2A | Metallothionein-2 | 1.43 | 3.07 |
|  | TIPRL | TIP41-like protein | 1.20 | 2.00 |
|  | SDHB | Succinate dehydrogenase [ubiquinone] iron-sulfur subunit, mitochondrial | 1.27 | 2.13 |
| 200 μg/ml  WSPM2.5 | HIST1H4A | Histone H4 | 0.645 | 0.236 |
|  | RPL27 | 60S ribosomal protein L27 | 0.721 | 0.296 |
|  | RPL18 | 60S ribosomal protein L18 | 0.740 | 0.341 |
|  | RPL18A | 60S ribosomal protein L18a | 0.749 | 0.289 |
|  | ARL6IP4 | ADP-ribosylation factor-like protein 6-interacting protein 4 | 0.749 | 0.324 |
|  | RPL38 | 60S ribosomal protein L38 | 0.769 | 0.679 |
|  | PXN | Paxillin | 0.777 | 0.489 |
|  | RPL6 | 60S ribosomal protein L6 | 0.78 | 0.398 |
|  | SNRPD1 | Small nuclear ribonucleoprotein Sm D1 | 0.781 | 0.429 |
|  | S100A11 | Protein S100-A11 | 0.789 | 0.597 |
|  | RPL13 | 60S ribosomal protein L13 | 0.801 | 0.177 |
|  | ECPAS | Proteasome adapter and scaffold protein ECM29 | 0.801 | 0.404 |
|  | KRT81 | Keratin, type II cuticular Hb1 | 0.802 | 0.434 |
|  | RPS18 | 40S ribosomal protein S18 | 0.807 | 0.369 |
|  | COPS6 | COP9 signalosome complex subunit 6 | 0.811 | 0.797 |
|  | RDX | Radixin | 0.815 | 0.493 |
|  | TGM2 | Protein-glutamine gamma-glutamyltransferase 2 | 0.816 | 0.477 |
|  | RPL7A | 60S ribosomal protein L7a | 0.818 | 0.370 |
|  | RPL14 | 60S ribosomal protein L14 | 0.832 | 0.289 |
|  | CYP1B1 | Cytochrome P450 1B1 | 1.48 | 2.72 |
|  | MT2A | Metallothionein-2 | 1.49 | 1.46 |
|  | HBA1 | Hemoglobin subunit alpha | 2.22 | 3.35 |

| **Table S5. The differentially expressed proteins were verified by Western blot.** | | | | |
| --- | --- | --- | --- | --- |
|  | Gene name | Protein name | iTRAQ vs. control | WB vs.  control |
| 50 μg/ml  WSPM10 | FAK | Focal adhesion kinase 1 | 0.723 | 0.532 |
|  | CDC27 | Cell division cycle protein 27 homolog | 1.306 | 1.697 |
|  | RPRD1A | Regulation of nuclear pre-mRNA domain-containing protein 1A | 1.334 | 1.632 |
|  | EIF6 | Eukaryotic translation initiation factor 6 | 0.712 | 0.625 |
| 50 μg/ml  WSPM2.5 | RPL34 | 60S ribosomal protein L34 | 0.694 | 0.563 |
|  | RPL24 | 60S ribosomal protein L24 | 0.754 | 0.464 |


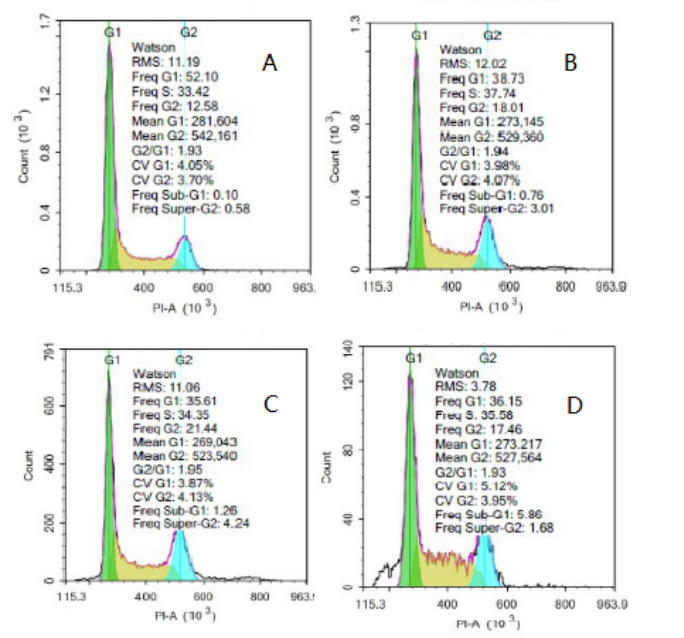


**Figure S1. The effect of WSPM10 on cell cycle for A549 cell. A549 cells were treated with concentrations of 0 μg/ml (A), 12.5 μg/ml (B), 50 μg/ml (C) and 200 μg/ml (D) WSPM10 for 24 h. After incubation with PI for 30min, cell cycle was analyzed by flow cytometry.**


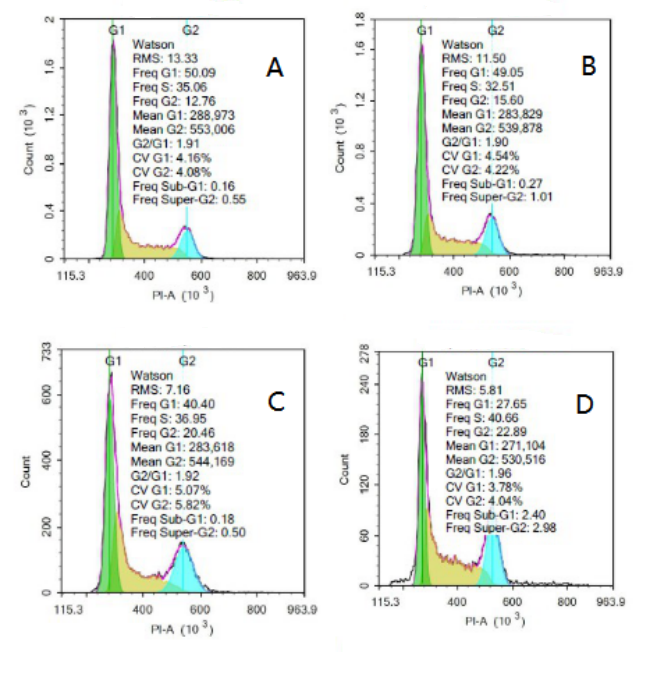


**Figure S2. The effect of WSPM2.5 on cell cycle for A549 cell. A549 cells were treated with 0 μg/ml (A), 12.5 μg/ml (B), 50 μg/ml (C) and 200 μg/ml (D) WSPM2.5 for 24 h. After incubation with PI for 30min, cell cycle was analyzed by flow cytometry.**


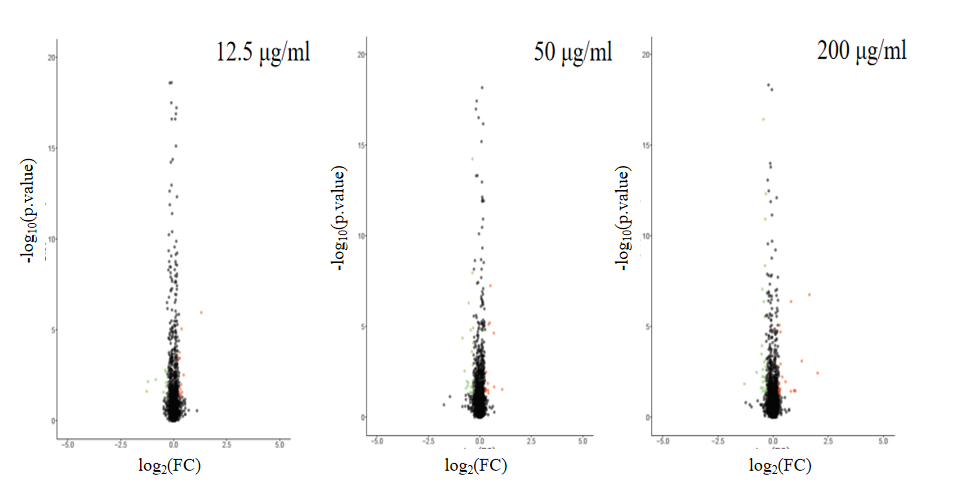


**Figure S3. Volcano plot of differential expression proteins of WSPM10 group. The distribution of differentially expressed proteins in A549 cells which were treated with different concentrations of WSPM10 for 24 h. The red dots indicated upregulated of protein expression and the green dots indicated downregulated of protein expression. R-code was used to screen the differentially expressed proteins from the mass spectrometry results, and the ggplot2 package was used to draw the volcano map.The abscissais the logarithm of FC, and the ordinate is the inverse of the logarithm of p value.**


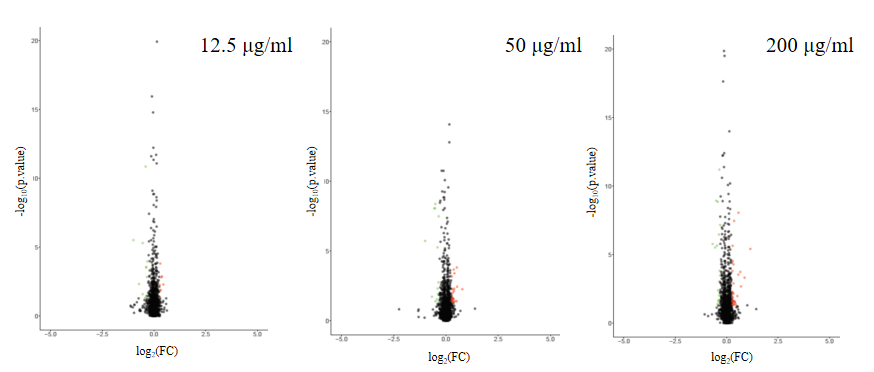


**Figure S4. Volcano plot of differential expression proteins of WSPM2.5 group. The distribution of differentially expressed proteins in A549 cells which were treated with different concentrations of WSPM2.5 for 24 h. The red dots indicated upregulated of protein expression and the green dots indicated downregulated of protein expression. R-code was used to screen the differentially expressed proteins from the mass spectrometry results, and the ggplot2 package was used to draw the volcano map.The abscissais the logarithm of FC, and the ordinate is the inverse of the logarithm of *p* value.**

Biological Process


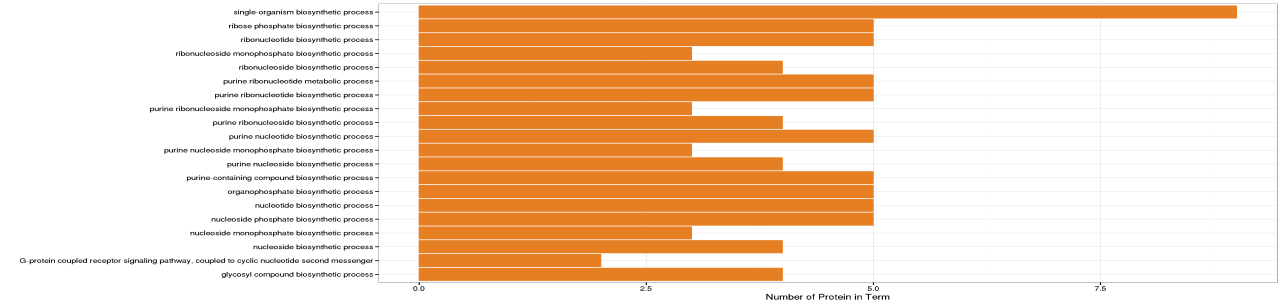

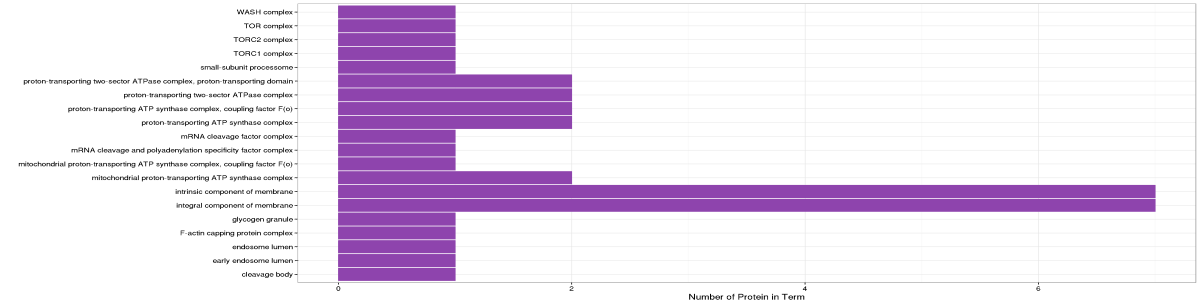

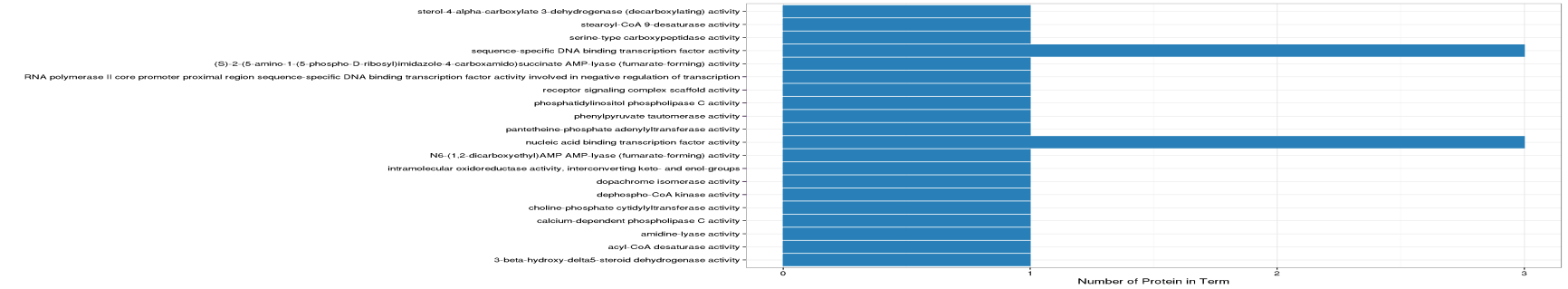


（a）WSPM10 (12.5 μg/ml)

Cellular component

Molecular Function

Biological Process


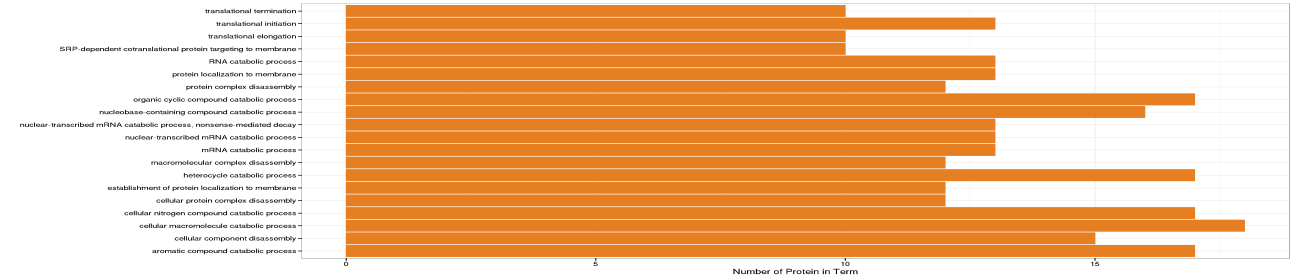

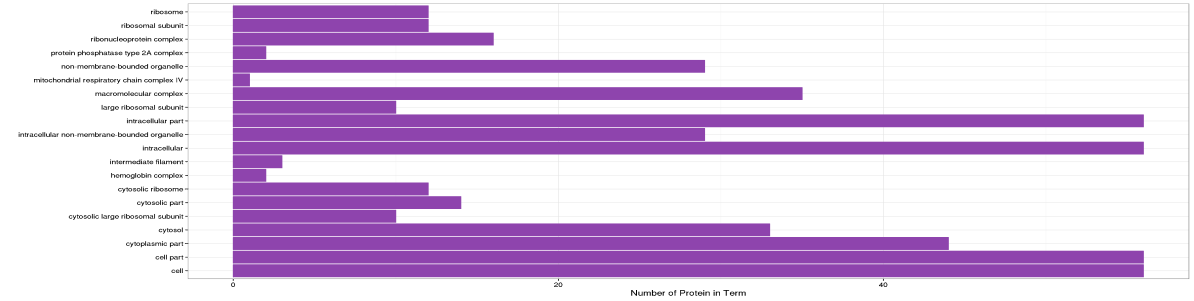

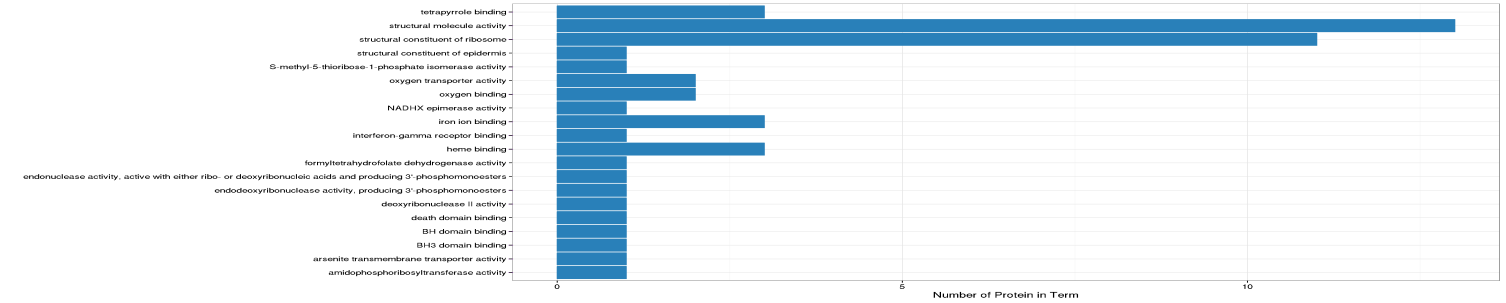


（b）WSPM10 (50 μg/ml)

Cellular component

Molecular Function


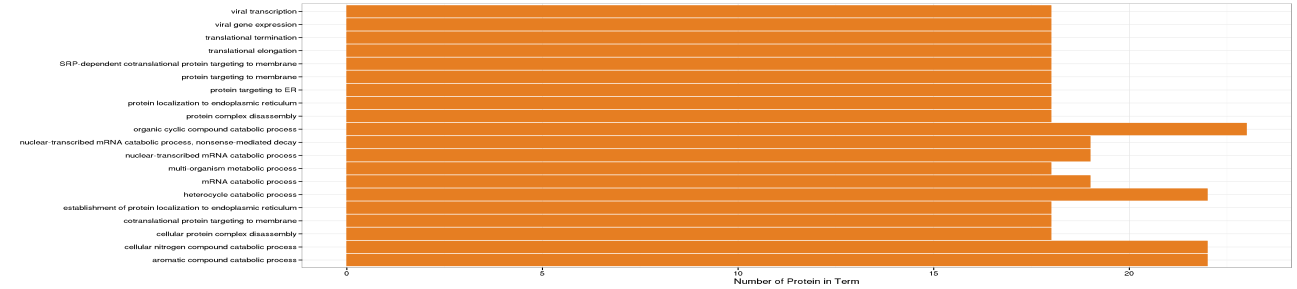

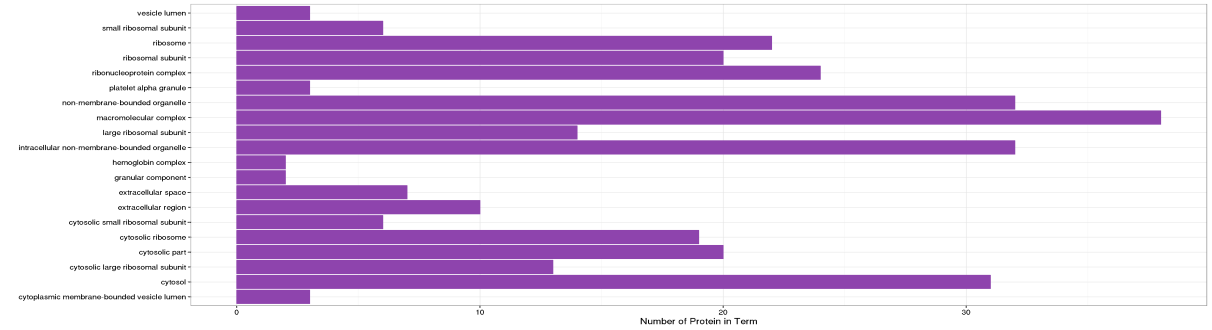

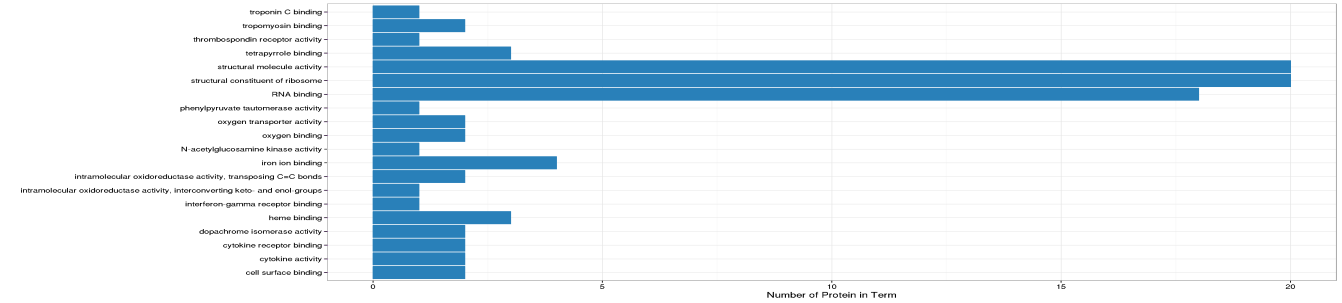


（c）WSPM10 (200 μg/ml)

Biological Process

Molecular Function

Cellular component

**Figure S5. GO enrichment analysis of group treated with 12.5 μg/ml (a), 50 μg/ml (b), 200 μg/ml (c) of WSPM10. The cutoff of *p*-value was set to 0.05. Terms in the same category were ordered based on the *p*-values.**


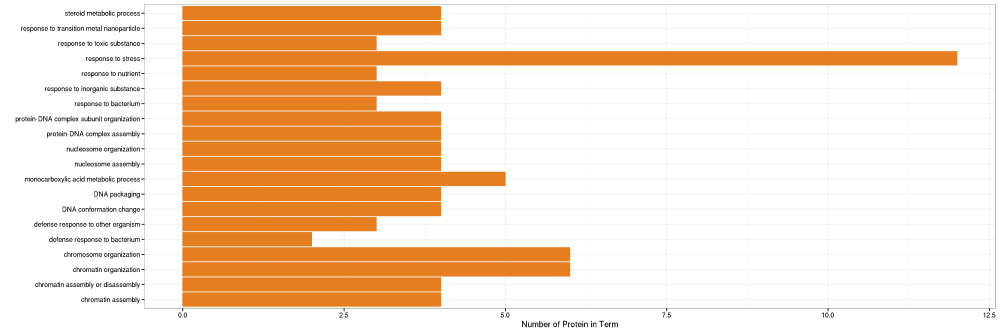

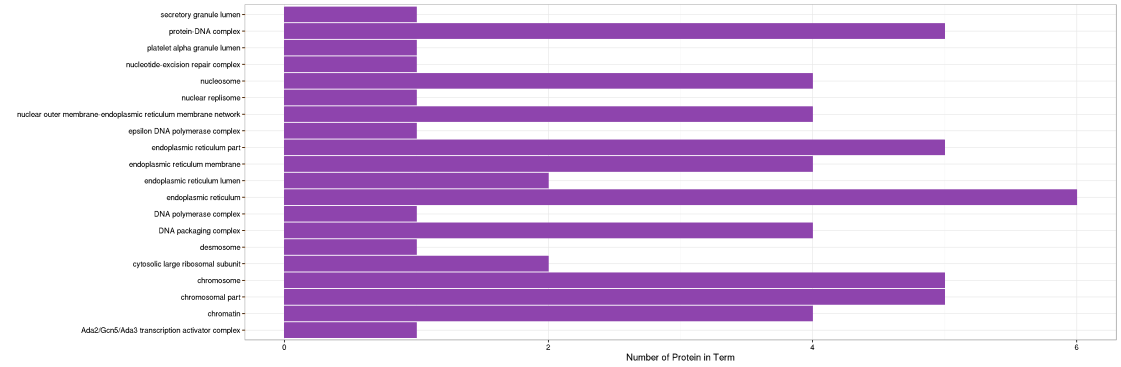

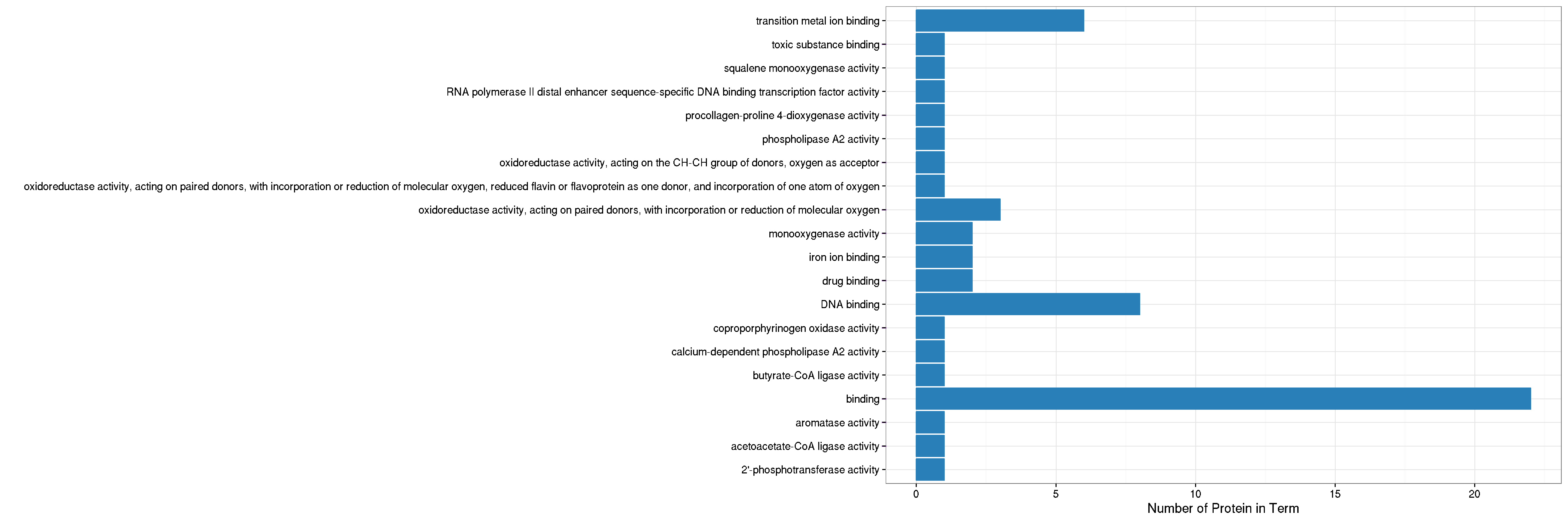


（a）WSPM2.5 (12.5 μg/ml)

Biological Process

Cellular component

Molecular Function


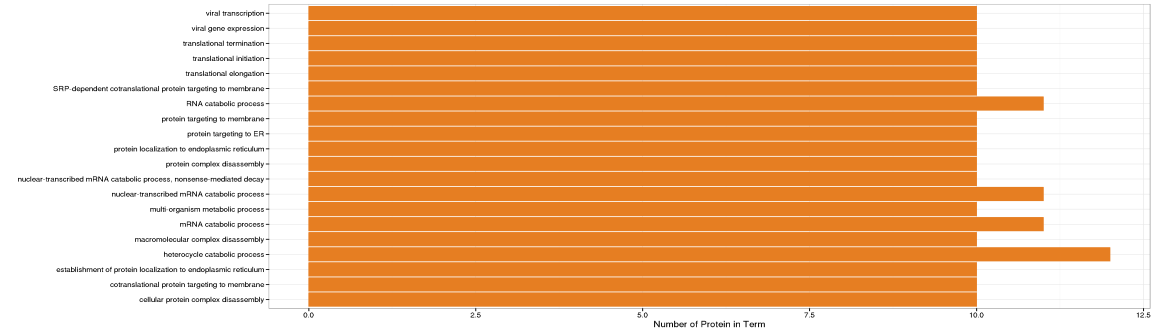

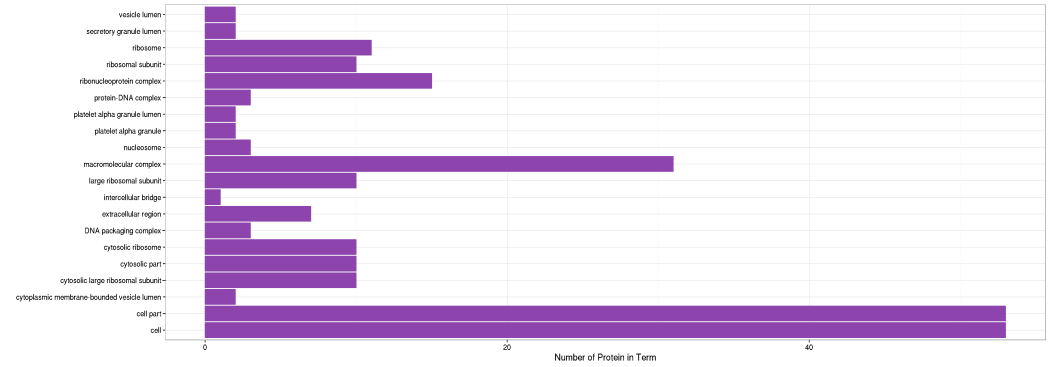

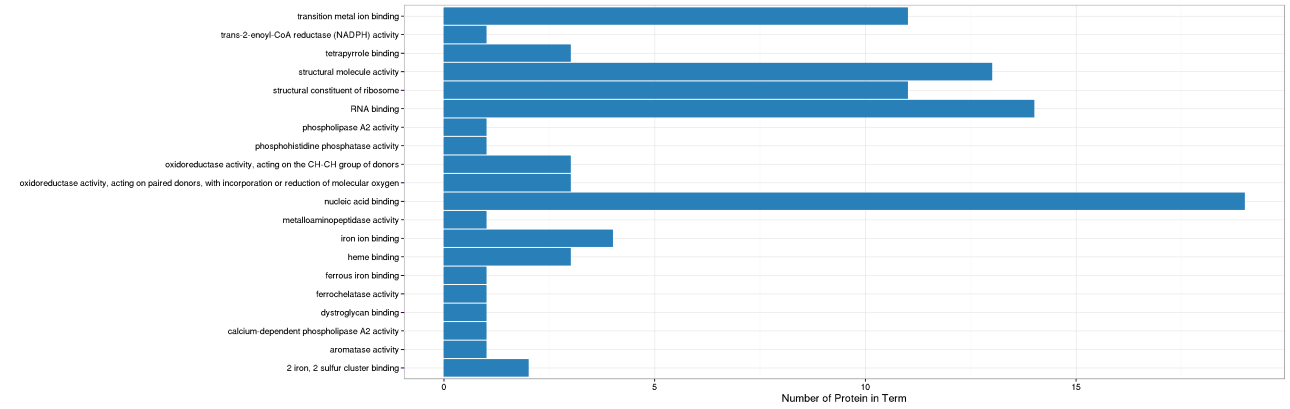


（b）WSPM2.5 (50 μg/ml)

Biological Process

Cellular component

Molecular Function

Biological Process


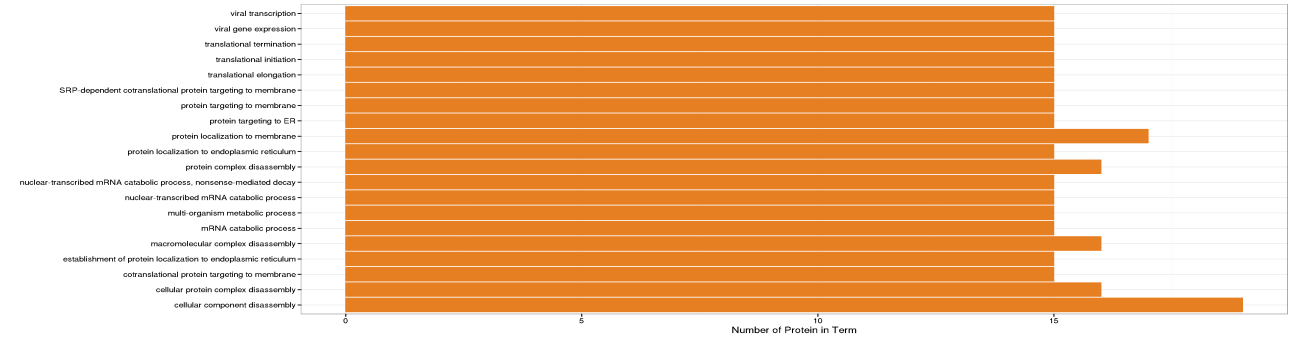

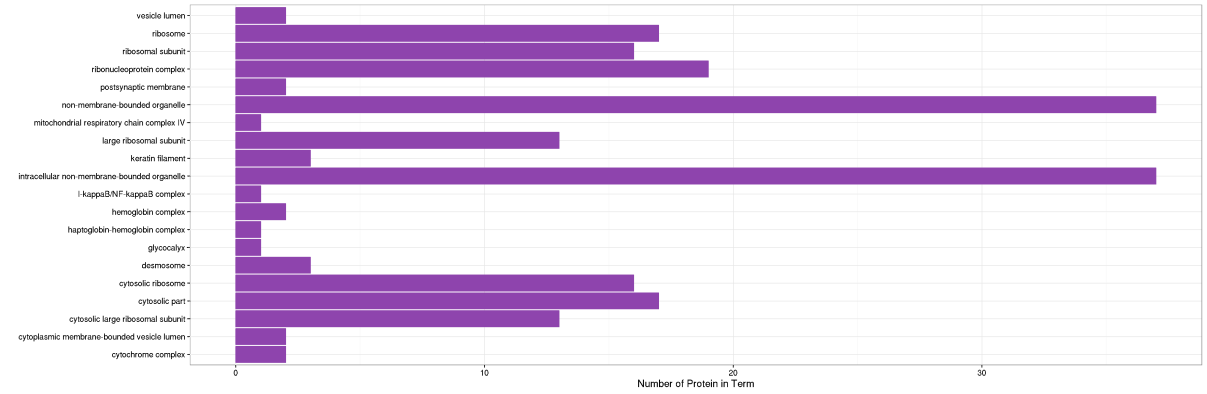

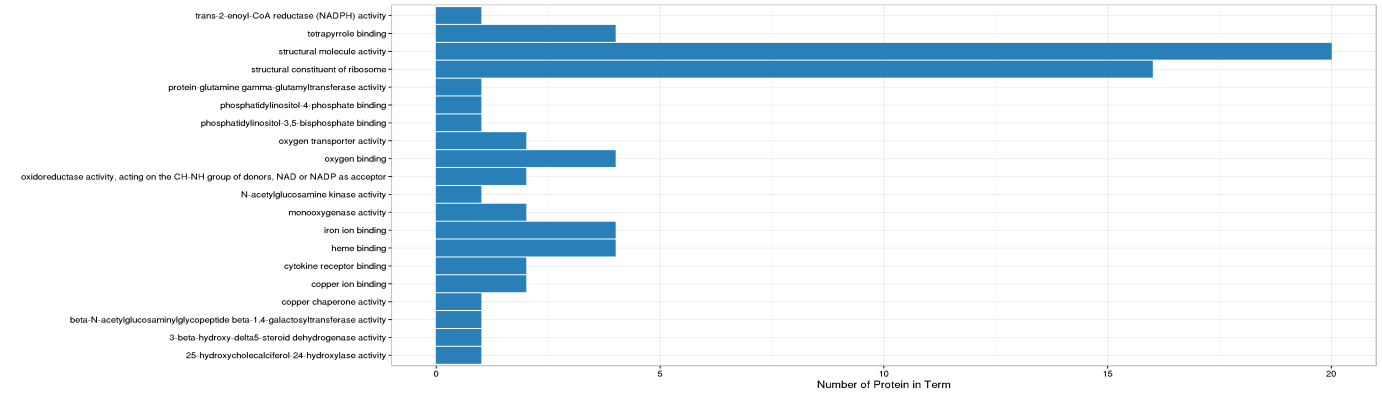


（c）WSPM2.5 (200 μg/ml)

Cellular component

Molecular Function

**Figure S6. GO enrichment analysis of group treated with 12.5 μg/ml (a), 50 μg/ml (b), 200 μg/ml (c) of WSPM2.5. The cutoff of *p*-value was set to 0.05. Terms in the same category were ordered based on the *p*-values.**


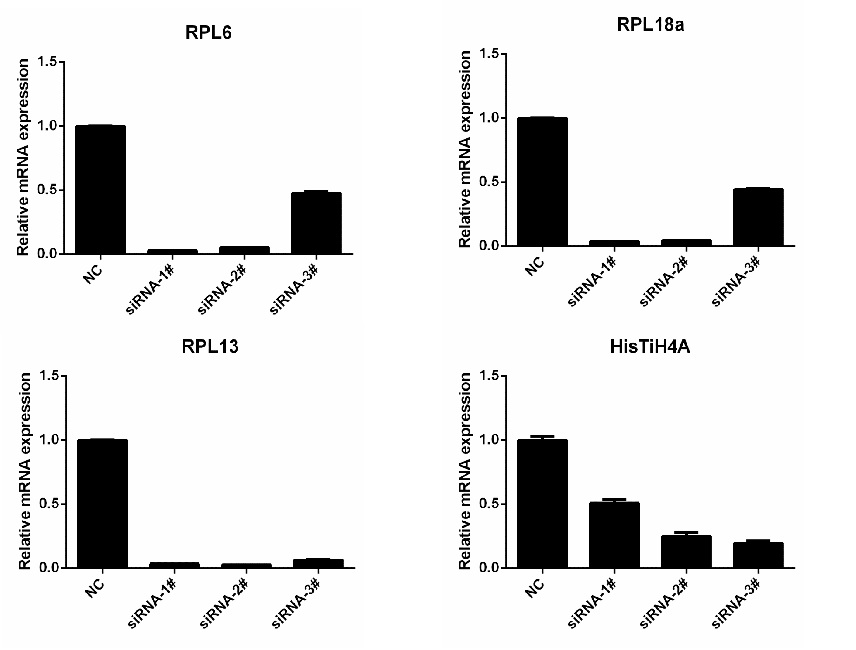


**Figure S7. The evaluation of RNA interference effect. The expression levels of *RPL6*、*RPL18A*、*RPL13* and *HisTIH4A* genes were measured by qRT-PCR at 24 h after siRNA transfection, respectively.**


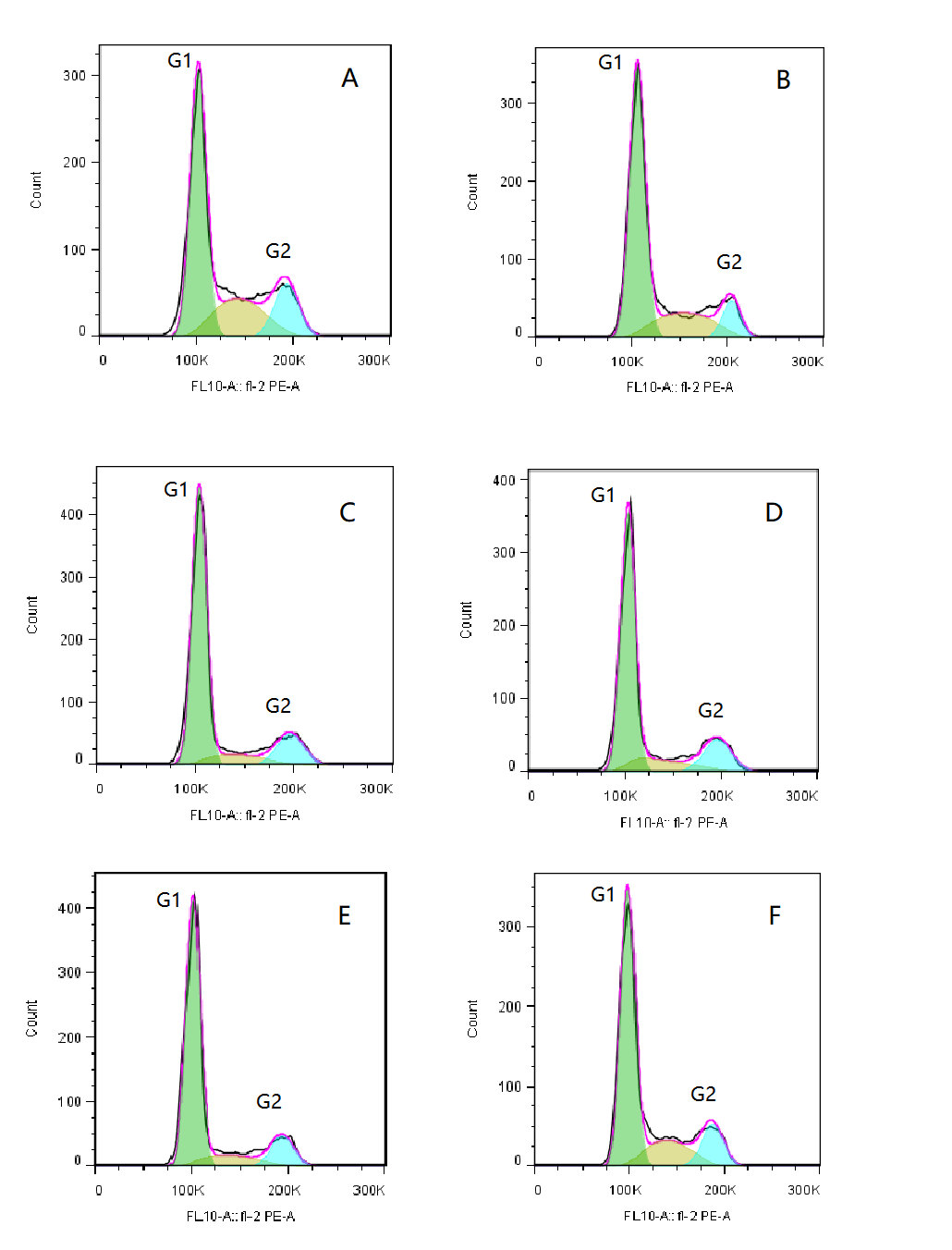


**Figure S8. The effect of *RPL13, RPL6, RPL18A or HIS1H4A* gene interference on cell cycle. The A549 cells were incubated with PI for 30 min, then cell cycle was detected by flow cytometry 48 h after siRNA transfection. (A) blank control group; (B) negative control group;(C) *RPL13* interference group; (D) *RPL6* interference group; (E) *RPL18A* interference group; (F) *HIS1H4A* interference group.**

| **Table S6. The effect of gene interference on cell cycle.** | | | |
| --- | --- | --- | --- |
| Group | G1（%） | S（%） | G2/M（%） |
| blank | 56.19±1.01 | 28.22±1.74 | 14.13±2.49 |
| NC | 59.02±0.72 | 26.20±1.72^＊^ | 13.28±1.48 |
| siRNA RPL13 | 75.08±1.27** | 10.12±0.23* | 13.10±1.30 |
| siRNA RPL6 | 68.72±0.36** | 12.62±1.50* | 17.72±1.32* |
| siRNA RPL18A | 72.75±0.94** | 11.27±1.01* | 14.94±0.71 |
| siRNA HIS1H4A | 63.41±0.54 | 17.31±1.06** | 16.69±0.75 |
| Note: ^＊^compared with blank group, *p* < 0.05;  * compared with NC group, *p* < 0.05； ** compared with NC group, *p* < 0.01.  the results were expressed as Mean±SD, n=3 | | | |

| **Table S7. Effects of LaCl_3_, CeCl_3_, NdCl_3_ and NaF on the proliferation inhibition rate of A549 cells.** | | | | |
| --- | --- | --- | --- | --- |
| [Concentration](C:/Users/xiayu/AppData/Local/youdao/dict/Application/8.5.1.0/resultui/html/index.html#/javascript:;) | 0.1 mM | 0.5 mM | 1 mM | 2 mM |
| LaCl_3_ | 39.63%±2.60% | 38.10%±1.33% | 41.60%±2.73% | 70.69%±2.56% |
| CeCl_3_ | 11.37%±1.21% | 17.98%±5.29% | 46.10%±3.11% | 74.85%±1.07% |
| NdCl_3_ | 46.53%±1.88% | 33.49%±0.67% | 42.84%±2.99% | 70.19%±6.04% |
| NaF | 10.85%±3.95% | 10.75%±1.87% | 54.69%±2.21% | 77.21%±1.12% |

Note: the results were expressed as Mean±SD, n=3


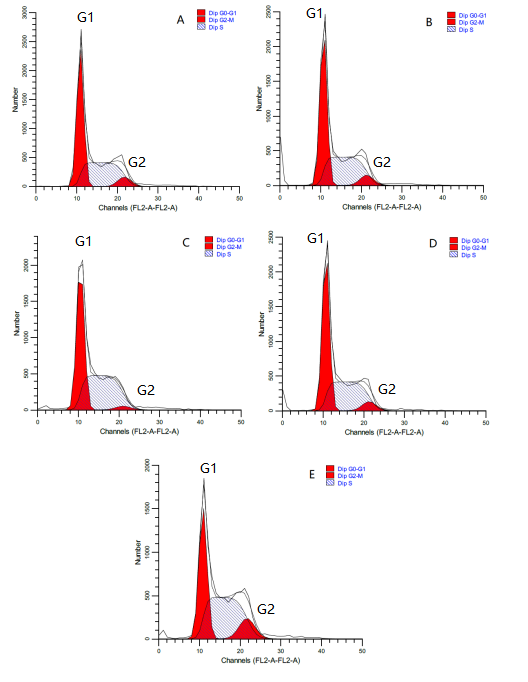


**Figure S9. Effects of** **LaCl_3_, CeCl_3_, NdCl_3_ and NaF on A549 cell cycle after exposure for 24 h. The solvent blank was used as control group（A）.Under the same concentration（2 mM）, A549 cells were treated with LaCl_3_ (B), CeCl_3_ (C), NdCl_3_（D）and NaF（E）for 24 h，respectively. After incubation with PI for 30 min, cell cycle was analyzed by flow cytometry.**

| **Table S8. Effects of LaCl_3_, CeCl_3_, NdCl_3_ and NaF on A549 cell cycle.** | | | |
| --- | --- | --- | --- |
| Group | G1（%） | S（%） | G2/M（%） |
| Control | 50.43±0.36 | 43.61±0.21 | 5.96±0.31 |
| LaCl_3_ | 49.65±0.29 | 45.11±0.55 | 5.25±0.44 |
| CeCl_3_ | 47.91±0.36* | 49.65±0.48** | 2.69±0.13 |
| NdCl_3_ | 50.89±0.41 | 44.02±0.35 | 5.08±0.21 |
| NaF | 38.81±0.70** | 49.93±0.67** | 11.26±0.40** |
| * compared with NC group, *p* < 0.05; ** compared with NC group, *p* < 0.01.  Note: the results were expressed as Mean±SD, n=3 | | | |


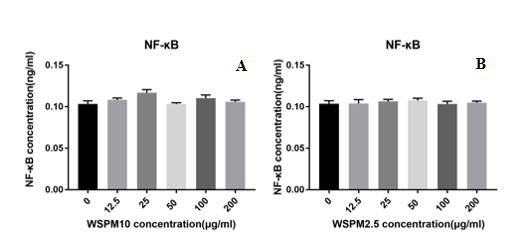


**Figure S10. Effects of WSPM on the protein expression of NF-κB. The abscissa is the exposure concentration of WSPM10 or WSPM2.5, and the ordinate is the expression of NF-κB. (A) and (B) were the NF-κB expression of A549 cells treated with different concentration (0, 12.5, 25, 50 and 200 μg/ml) of WSPM10 and WSPM2.5 for 24 h.* compared with the NC group, *p* < 0.05; ** compared with the NC group, *p* < 0.01.**


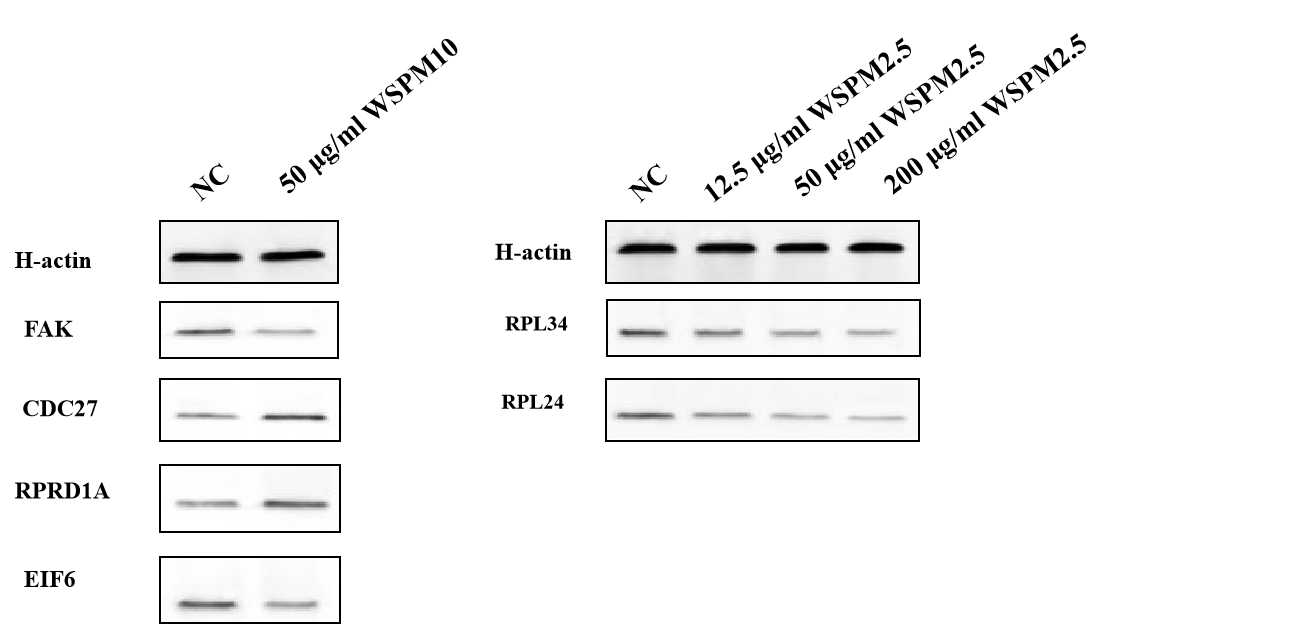

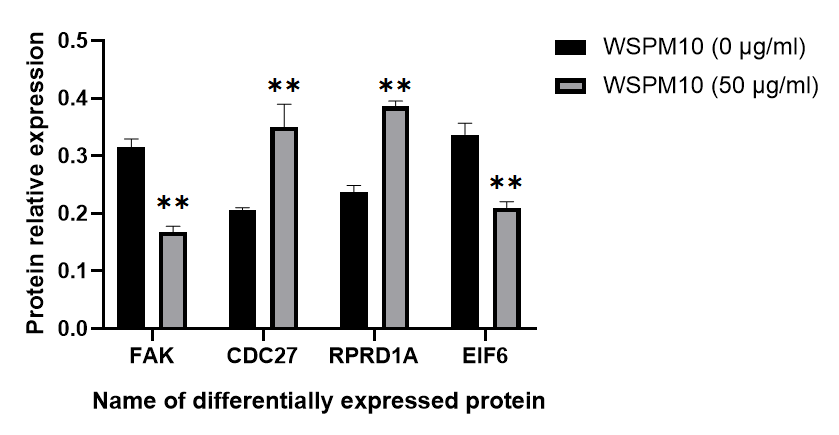


**（A）**


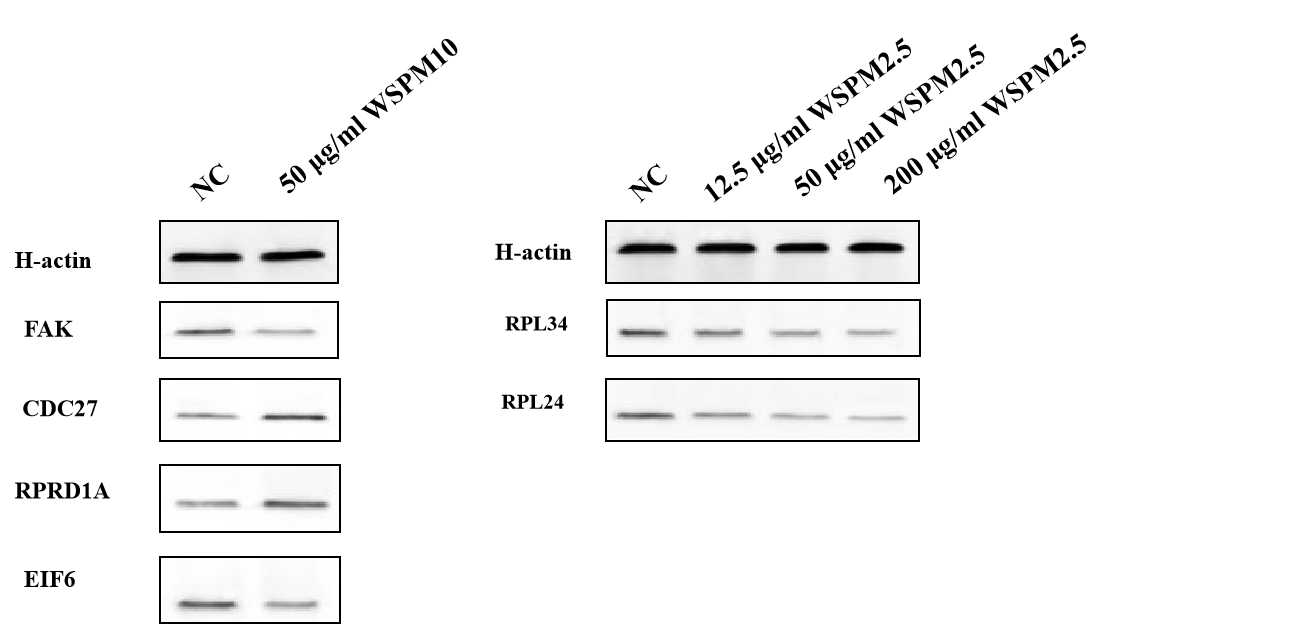

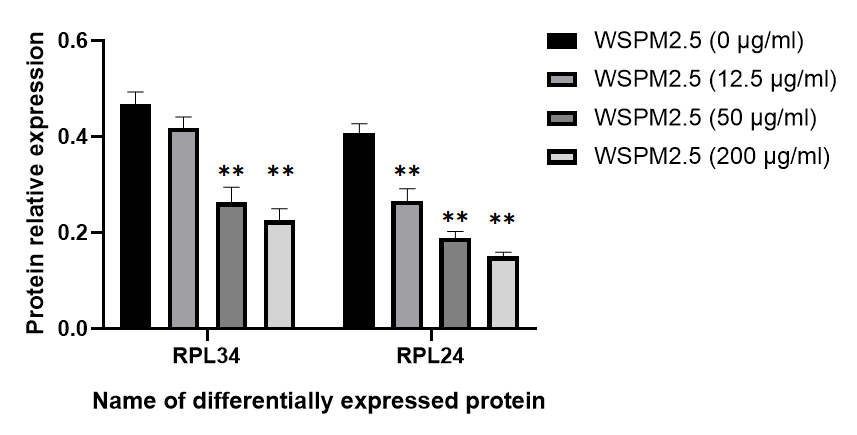


**（B）**

**Figure S11. A549 cells were treated with WSPM10 (50 μg/ml) (A) and WSPM2.5 (12.5, 50, and 200 μg/ml) (B) for 24 hours. Cell lysates were immunoblotted with the indicated antibodies. A549 cells treated with either DMSO was used as negative control (NC). Each experiment was repeated three times independently. * compared with the control group (0 μg/ml), *p* < 0.05; ** compared with the control group (0 μg/ml), *p* < 0.01.**


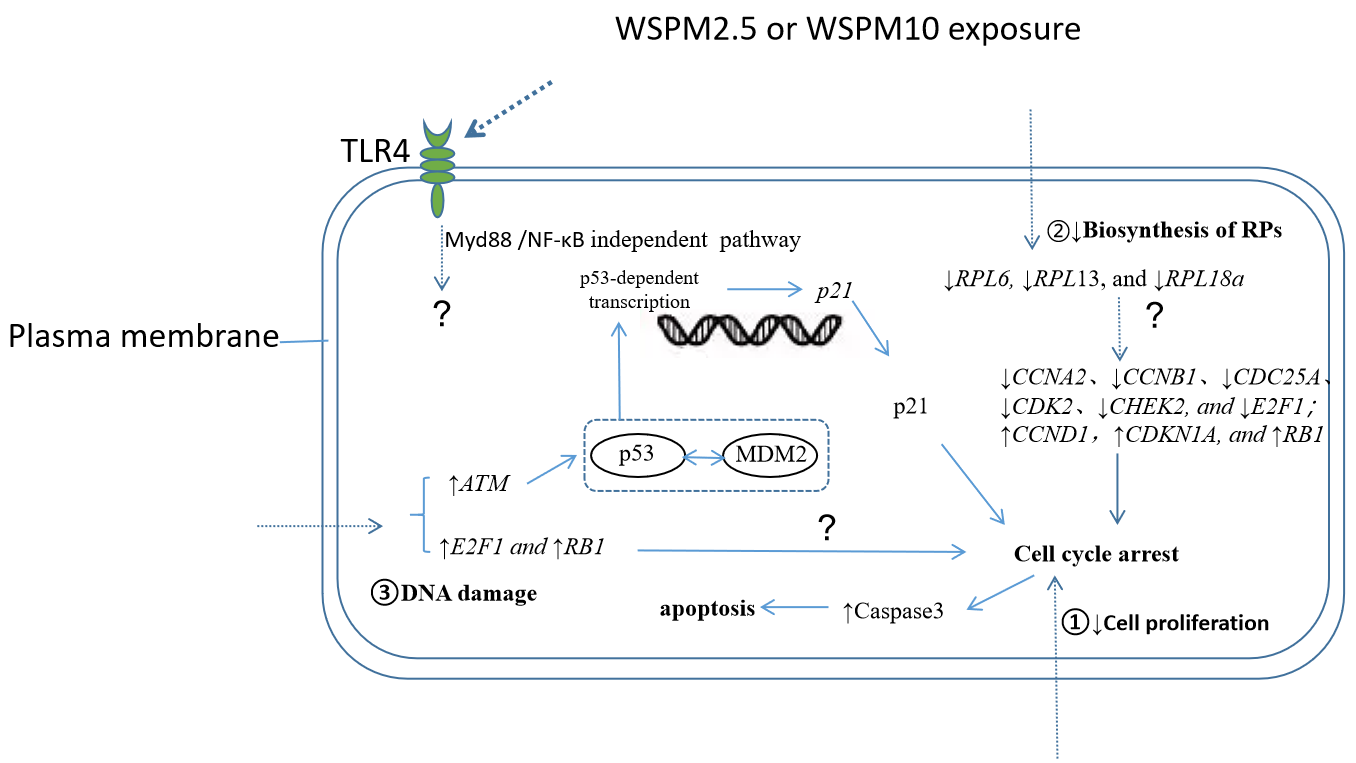


**Figure S12. Diagram of potential toxicity mechanism of WSPM for A549 cells. ‘↑’ is the upregulated expression of protein; ‘↓’ is the downregulated expression of protein. Each relationship is shown with a solid blue arrow. The broken lines with arrows represent may be a potential connections.**


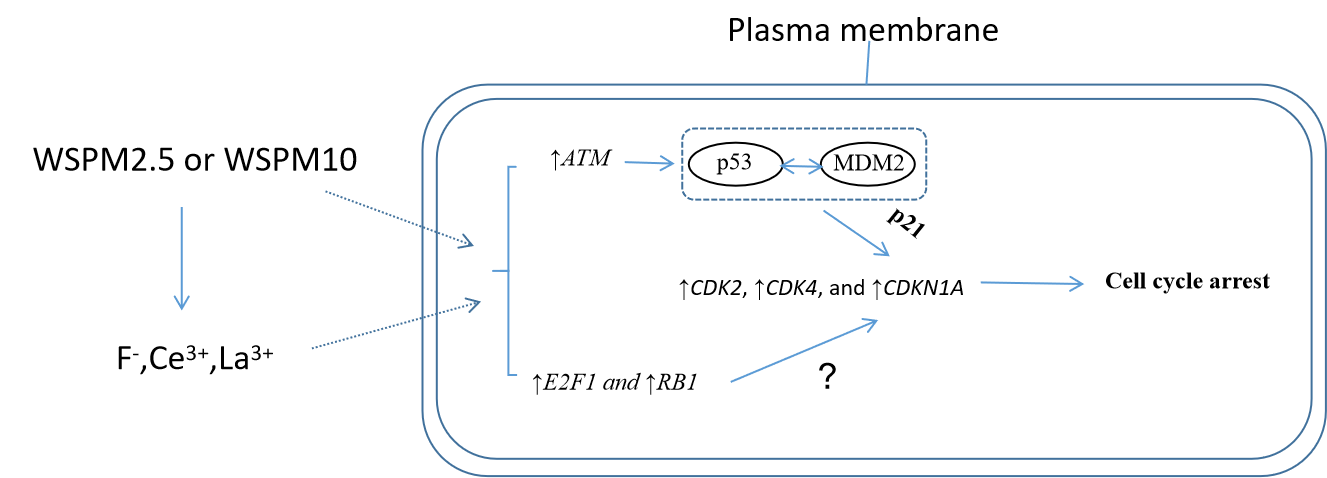


**Figure S13. The influence of WSPM on A549 cell cycle and its potential mechanism. ‘↑’ is the upregulated expression of protein; ‘↓’ is the downregulated expression of protein. Each relationship is shown with a solid blue arrow. The broken lines with arrows represent may be a potential connections.**

| **Table S9. Significance enrichment analysis of differentially expressed proteins involved in biological processes in 12.5 μg/ml WSPM10 group.** | | | |
| --- | --- | --- | --- |
| Term | Description | Cluster frequency | p-value |
| GO:0009163 | nucleoside biosynthetic process | 4 / 47, 8.51% | 0.001503819 |
| GO:0009260 | ribonucleotide biosynthetic process | 5 / 53, 9.43% | 0.000218857 |
| GO:0009259 | ribonucleotide metabolic process | 5 / 113, 4.42% | 0.006791722 |
| GO:0042776 | mitochondrial ATP synthesis coupled proton transport | 2 / 14, 14.29% | 0.00978838 |
| GO:0072522 | purine-containing compound biosynthetic process | 5 / 55, 9.09% | 0.000261326 |
| GO:0009168 | purine ribonucleoside monophosphate biosynthetic process | 3 / 31, 9.68% | 0.004355055 |
| GO:0006164 | purine nucleotide biosynthetic process | 5 / 49, 10.20% | 0.000149893 |
| GO:0007187 | G-protein coupled receptor signaling pathway, coupled to cyclic nucleotide second messenger | 2 / 8, 25.00% | 0.003137056 |
| GO:0043631 | RNA polyadenylation | 2 / 14, 14.29% | 0.00978838 |
| GO:0015985 | energy coupled proton transport, down electrochemical gradient | 2 / 14, 14.29% | 0.00978838 |
| GO:0006378 | mRNA polyadenylation | 2 / 14, 14.29% | 0.00978838 |
| GO:0044281 | small molecule metabolic process | 12 / 556, 2.16% | 0.009568038 |
| GO:0009152 | purine ribonucleotide biosynthetic process | 5 / 47, 10.64% | 0.000122414 |
| GO:0006695 | cholesterol biosynthetic process | 2 / 13, 15.38% | 0.008447125 |
| GO:1901293 | nucleoside phosphate biosynthetic process | 5 / 78, 6.41% | 0.001332399 |
| GO:0042451 | purine nucleoside biosynthetic process | 4 / 38, 10.53% | 0.000666066 |
| GO:0009165 | nucleotide biosynthetic process | 5 / 78, 6.41% | 0.001332399 |
| GO:0044711 | single-organism biosynthetic process | 9 / 277, 3.25% | 0.001946252 |
| GO:0016126 | sterol biosynthetic process | 2 / 13, 15.38% | 0.008447125 |
| GO:0006163 | purine nucleotide metabolic process | 5 / 115, 4.35% | 0.007314076 |
| GO:0019693 | ribose phosphate metabolic process | 5 / 121, 4.13% | 0.009051102 |
| GO:0090407 | organophosphate biosynthetic process | 5 / 108, 4.63% | 0.005602515 |
| GO:0072521 | purine-containing compound metabolic process | 5 / 122, 4.10% | 0.009366381 |
| GO:0015986 | ATP synthesis coupled proton transport | 2 / 14, 14.29% | 0.00978838 |
| GO:0009127 | purine nucleoside monophosphate biosynthetic process | 3 / 31, 9.68% | 0.004355055 |
| GO:0042455 | ribonucleoside biosynthetic process | 5 / 56, 8.89% | 0.001275293 |
| GO:0046390 | ribose phosphate biosynthetic process | 4 / 47, 8.93% | 0.000284784 |
| GO:1901659 | glycosyl compound biosynthetic process | 5 / 56, 8.51% | 0.001503819 |
| GO:0009150 | purine ribonucleotide metabolic process | 5 / 107, 4.67% | 0.005383899 |
| GO:0046129 | purine ribonucleoside biosynthetic process | 4 / 38, 10.53% | 0.000666066 |
| GO:1901137 | carbohydrate derivative biosynthetic process | 5 / 116, 4.31% | 0.007585639 |
| GO:0009156 | ribonucleoside monophosphate biosynthetic process | 3 / 35, 8.57% | 0.001503819 |
|  |  |  |  |

| **Table S10. Significance enrichment analysis of differentially expressed proteins involved in biological processes in 50 μg/ml WSPM10 group.** | | | |
| --- | --- | --- | --- |
| Term | Description | Cluster frequency | p-value |
| GO:0071346 | cellular response to interferon-gamma | 3 / 14, 21.43% | 0.003432831 |
| GO:0046700 | heterocycle catabolic process | 17 / 175, 9.71% | 1.25695E-07 |
| GO:0044248 | cellular catabolic process | 21 / 397, 5.29% | 7.91517E-05 |
| GO:0043933 | macromolecular complex subunit organization | 23 / 492, 4.67% | 0.000221191 |
| GO:0008635 | activation of cysteine-type endopeptidase activity involved in apoptotic process by cytochrome c | 2 / 4, 50.00% | 0.002982921 |
| GO:0043029 | T cell homeostasis | 2 / 3, 66.67% | 0.001513658 |
| GO:0010942 | positive regulation of cell death | 6 / 69, 8.70% | 0.004259354 |
| GO:0070661 | leukocyte proliferation | 3 / 17, 17.65% | 0.006108796 |
| GO:0016482 | cytoplasmic transport | 14 / 283, 4.95% | 0.0034387 |
| GO:0042102 | positive regulation of T cell proliferation | 2 / 5, 40.00% | 0.004898741 |
| GO:0044033 | multi-organism metabolic process | 10 / 102, 9.80% | 7.14479E-05 |
| GO:0016337 | single organismal cell-cell adhesion | 5 / 56, 8.93% | 0.008139222 |
| GO:0042098 | T cell proliferation | 3 / 11, 27.27% | 0.001633777 |
| GO:0006413 | translational initiation | 13 / 113, 11.50% | 7.51029E-07 |
| GO:0070665 | positive regulation of leukocyte proliferation | 2 / 7, 28.57% | 0.009989013 |
| GO:0071887 | leukocyte apoptotic process | 2 / 6, 33.33% | 0.007240691 |
| GO:0045947 | negative regulation of translational initiation | 2 / 5, 40.00% | 0.004898741 |
| GO:0034613 | cellular protein localization | 14 / 306, 4.58% | 0.007030351 |
| GO:0061024 | membrane organization | 15 / 217, 6.91% | 5.97184E-05 |
| GO:0044802 | single-organism membrane organization | 15 / 211, 7.11% | 4.27834E-05 |
| GO:0000184 | nuclear-transcribed mRNA catabolic process, nonsense-mediated decay | 13 / 96, 13.54% | 1.05312E-07 |
| GO:0072599 | establishment of protein localization to endoplasmic reticulum | 10 / 95, 10.53% | 3.83346E-05 |
| GO:0071822 | protein complex subunit organization | 20 / 380, 5.26% | 0.000137281 |
| GO:0050671 | positive regulation of lymphocyte proliferation | 2 / 7, 28.57% | 0.009989013 |
| GO:0070227 | lymphocyte apoptotic process | 2 / 4, 50.00% | 0.002982921 |
| GO:0006919 | activation of cysteine-type endopeptidase activity involved in apoptotic process | 3 / 13, 23.08% | 0.002741345 |
| GO:0006402 | mRNA catabolic process | 13 / 125, 10.40% | 2.44222E-06 |
| GO:0070972 | protein localization to endoplasmic reticulum | 10 / 98, 10.20% | 5.04028E-05 |
| GO:0022411 | cellular component disassembly | 15 / 156, 9.62% | 9.63273E-07 |
| GO:0097202 | activation of cysteine-type endopeptidase activity | 3 / 14, 21.43% | 0.003432831 |
| GO:0072594 | establishment of protein localization to organelle | 11 / 186, 5.91% | 0.002558152 |
| GO:1902580 | single-organism cellular localization | 16 / 262, 6.11% | 0.000145532 |
| GO:0043241 | protein complex disassembly | 12 / 109, 11.01% | 3.48695E-06 |
| GO:0010623 | developmental programmed cell death | 2 / 4, 50.00% | 0.002982921 |
| GO:0046651 | lymphocyte proliferation | 3 / 16, 18.75% | 0.005112855 |
| GO:1902742 | apoptotic process involved in development | 2 / 3, 66.67% | 0.001513658 |
| GO:2001056 | positive regulation of cysteine-type endopeptidase activity | 3 / 20, 15.00% | 0.009756679 |
| GO:0010952 | positive regulation of peptidase activity | 3 / 20, 15.00% | 0.009756679 |
| GO:0033365 | protein localization to organelle | 12 / 202, 5.94% | 0.001521485 |
| GO:0002260 | lymphocyte homeostasis | 2 / 5, 40.00% | 0.004898741 |
| GO:0006308 | DNA catabolic process | 3 / 13, 23.08% | 0.002741345 |
| GO:0006412 | translation | 14 / 249, 5.62% | 0.000990431 |
| GO:0044270 | cellular nitrogen compound catabolic process | 17 / 177, 9.60% | 1.49044E-07 |
| GO:0001974 | blood vessel remodeling | 2 / 4, 50.00% | 0.002982921 |
| GO:0043624 | cellular protein complex disassembly | 12 / 101, 11.88% | 1.52217E-06 |
| GO:1902578 | single-organism localization | 25 / 584, 4.28% | 0.00042668 |
| GO:0032984 | macromolecular complex disassembly | 12 / 113, 10.62% | 5.13269E-06 |
| GO:0006415 | translational termination | 10 / 81, 12.35% | 9.11099E-06 |
| GO:0019083 | viral transcription | 10 / 99, 10.10% | 5.50886E-05 |
| GO:0019058 | viral life cycle | 10 / 132, 7.58% | 0.000616516 |
| GO:0043280 | positive regulation of cysteine-type endopeptidase activity involved in apoptotic process | 3 / 19, 15.79% | 0.008428132 |
| GO:0009056 | catabolic process | 21 / 449, 4.68% | 0.000484223 |
| GO:0000956 | nuclear-transcribed mRNA catabolic process | 13 / 121, 10.74% | 1.67546E-06 |
| GO:0006613 | cotranslational protein targeting to membrane | 10 / 92, 10.87% | 2.88354E-05 |
| GO:0019439 | aromatic compound catabolic process | 17 / 177, 9.60% | 1.49044E-07 |
| GO:0006612 | protein targeting to membrane | 10 / 104, 9.62% | 8.45202E-05 |
| GO:0090150 | establishment of protein localization to membrane | 12 / 133, 9.02% | 2.81997E-05 |
| GO:0006401 | RNA catabolic process | 13 / 132, 9.85% | 4.55976E-06 |
| GO:0032946 | positive regulation of mononuclear cell proliferation | 2 / 7, 28.57% | 0.009989013 |
| GO:0044765 | single-organism transport | 22 / 555, 3.96% | 0.00340079 |
| GO:0009057 | macromolecule catabolic process | 18 / 296, 6.08% | 5.06996E-05 |
| GO:0010950 | positive regulation of endopeptidase activity | 3 / 20, 15.00% | 0.009756679 |
| GO:0034655 | nucleobase-containing compound catabolic process | 16 / 164, 9.76% | 3.08538E-07 |
| GO:0032943 | mononuclear cell proliferation | 3 / 16, 18.75% | 0.005112855 |
| GO:0031638 | zymogen activation | 3 / 15, 20.00% | 0.004222033 |
| GO:0006414 | translational elongation | 10 / 87, 11.49% | 1.7467E-05 |
| GO:0001776 | leukocyte homeostasis | 2 / 7, 28.57% | 0.009989013 |
| GO:0045047 | protein targeting to ER | 10 / 94, 10.64% | 3.49075E-05 |
| GO:0006614 | SRP-dependent cotranslational protein targeting to membrane | 10 / 91, 10.99% | 2.61565E-05 |
| GO:0034105 | positive regulation of tissue remodeling | 2 / 5, 40.00% | 0.004898741 |
| GO:0044265 | cellular macromolecule catabolic process | 18 / 263, 6.84% | 9.6715E-06 |
| GO:0060561 | apoptotic process involved in morphogenesis | 2 / 3, 66.67% | 0.001513658 |
| GO:1901575 | organic substance catabolic process | 20 / 427, 4.68% | 0.00069438 |
| GO:0022603 | regulation of anatomical structure morphogenesis | 6 / 82, 7.32% | 0.009900757 |
| GO:1901361 | organic cyclic compound catabolic process | 17 / 185, 9.19% | 2.87788E-07 |
| GO:0034341 | response to interferon-gamma | 3 / 17, 17.65% | 0.006108796 |
| GO:0019080 | viral gene expression | 10 / 101, 9.90% | 6.55872E-05 |
| GO:0051129 | negative regulation of cellular component organization | 6 / 81, 7.41% | 0.009338822 |
| GO:0070727 | cellular macromolecule localization | 14 / 308, 4.55% | 0.007450061 |
| GO:0072657 | protein localization to membrane | 13 / 143, 9.09% | 1.12252E-05 |

| **Table S11. Significance enrichment analysis of differentially expressed proteins involved in biological processes in 200 μg/ml WSPM10 group.** | | | |
| --- | --- | --- | --- |
| Term | Description | Cluster frequency | p-value |
| GO:0070727 | cellular macromolecule localization | 25 / 308，8.12% | 3.23031E-08 |
| GO:0000956 | nuclear-transcribed mRNA catabolic process | 19 / 121，15.70% | 3.61234E-11 |
| GO:0002260 | lymphocyte homeostasis | 2 / 5，40.00% | 0.006176684 |
| GO:0034654 | nucleobase-containing compound biosynthetic process | 25 / 499，5.01% | 0.00028687 |
| GO:1901361 | organic cyclic compound catabolic process | 23 / 185，12.43% | 2.67463E-11 |
| GO:0044248 | cellular catabolic process | 27 / 397，6.80% | 3.24428E-07 |
| GO:0019083 | viral transcription | 18 / 99，18.18% | 1.00582E-11 |
| GO:0019058 | viral life cycle | 18 / 132，13.64% | 1.50361E-09 |
| GO:0019538 | protein metabolic process | 39 / 805，4.84% | 1.53681E-06 |
| GO:0032946 | positive regulation of mononuclear cell proliferation | 3 / 7，42.86% | 0.000525303 |
| GO:0046907 | intracellular transport | 23 / 421，5.46% | 0.000152109 |
| GO:0050865 | regulation of cell activation | 4 / 30，13.33% | 0.006561486 |
| GO:0016482 | cytoplasmic transport | 22 / 283，7.77% | 6.75874E-07 |
| GO:0033036 | macromolecule localization | 29 / 506，5.73% | 3.94761E-06 |
| GO:1902580 | single-organism cellular localization | 23 / 262，8.78% | 3.29226E-08 |
| GO:0019080 | viral gene expression | 18 / 101，17.82% | 1.43938E-11 |
| GO:0016485 | protein processing | 4 / 33，12.12% | 0.009249692 |
| GO:0009059 | macromolecule biosynthetic process | 27 / 668，4.04% | 0.004886072 |
| GO:0045745 | positive regulation of G-protein coupled receptor protein signaling pathway | 2 / 4，50.00% | 0.003768307 |
| GO:0000002 | mitochondrial genome maintenance | 2 / 6，33.33% | 0.009112139 |
| GO:0016043 | cellular component organization | 36 / 912，3.95% | 0.000871174 |
| GO:0032774 | RNA biosynthetic process | 23 / 417，5.52% | 0.000130709 |
| GO:0071702 | organic substance transport | 27 / 459，5.88% | 6.46648E-06 |
| GO:0044419 | interspecies interaction between organisms | 21 / 276，7.61% | 1.93292E-06 |
| GO:0051179 | localization | 33 / 776，4.25% | 0.000427718 |
| GO:0044271 | cellular nitrogen compound biosynthetic process | 25 / 539，4.64% | 0.000988021 |
| GO:0006264 | mitochondrial DNA replication | 2 / 4，50.00% | 0.003768307 |
| GO:0019438 | aromatic compound biosynthetic process | 26 / 514，5.06% | 0.000168878 |
| GO:0006401 | RNA catabolic process | 19 / 132，14.39% | 1.76448E-10 |
| GO:0015031 | protein transport | 23 / 376，6.12% | 2.39272E-05 |
| GO:0006614 | SRP-dependent cotranslational protein targeting to membrane | 18 / 91，19.78% | 2.18468E-12 |
| GO:0061045 | negative regulation of wound healing | 2 / 5，40.00% | 0.006176684 |
| GO:0051234 | establishment of localization | 31 / 667，4.65% | 0.000133929 |
| GO:0070972 | protein localization to endoplasmic reticulum | 18 / 98，18.37% | 8.38023E-12 |
| GO:0045184 | establishment of protein localization | 24 / 386，6.22% | 1.0794E-05 |
| GO:0051704 | multi-organism process | 24 / 396，6.06% | 1.69905E-05 |
| GO:0072657 | protein localization to membrane | 20 / 143，13.99% | 8.79822E-11 |
| GO:0042989 | sequestering of actin monomers | 2 / 4，50.00% | 0.003768307 |
| GO:0000184 | nuclear-transcribed mRNA catabolic process, nonsense-mediated decay | 19 / 96，19.79% | 4.58174E-13 |
| GO:0070227 | lymphocyte apoptotic process | 2 / 4，50.00% | 0.003768307 |
| GO:0072594 | establishment of protein localization to organelle | 19 / 186，10.22% | 6.72281E-08 |
| GO:0044403 | symbiosis, encompassing mutualism through parasitism | 21 / 276，7.61% | 1.93292E-06 |
| GO:0044764 | multi-organism cellular process | 21 / 271，7.75% | 1.42113E-06 |
| GO:0006413 | translational initiation | 18 / 113，15.93% | 1.04594E-10 |
| GO:0032943 | mononuclear cell proliferation | 3 / 16，18.75% | 0.007122408 |
| GO:1903317 | regulation of protein maturation | 2 / 4，50.00% | 0.003768307 |
| GO:0044802 | single-organism membrane organization | 22 / 211，10.43% | 2.81182E-09 |
| GO:0032944 | regulation of mononuclear cell proliferation | 3 / 11，27.27% | 0.002300328 |
| GO:0070665 | positive regulation of leukocyte proliferation | 3 / 7，42.86% | 0.000525303 |
| GO:0070663 | regulation of leukocyte proliferation | 3 / 12，25.00% | 0.003011197 |
| GO:0006886 | intracellular protein transport | 21 / 257，8.17% | 5.75389E-07 |
| GO:0043170 | macromolecule metabolic process | 43 / 1273，3.38% | 0.005690658 |
| GO:0032042 | mitochondrial DNA metabolic process | 2 / 5，40.00% | 0.006176684 |
| GO:0044033 | multi-organism metabolic process | 18 / 102，17.65% | 1.71634E-11 |
| GO:0090150 | establishment of protein localization to membrane | 19 / 133，14.29% | 2.0218E-10 |
| GO:0044265 | cellular macromolecule catabolic process | 22 / 263，8.37% | 1.80385E-07 |
| GO:0046651 | lymphocyte proliferation | 3 / 16，18.75% | 0.007122408 |
| GO:1901360 | organic cyclic compound metabolic process | 36 / 1013，3.55% | 0.007376077 |
| GO:0006612 | protein targeting to membrane | 18 / 104，17.31% | 2.42529E-11 |
| GO:0032984 | macromolecular complex disassembly | 18 / 113，15.93% | 1.04594E-10 |
| GO:0030195 | negative regulation of blood coagulation | 2 / 4，50.00% | 0.003768307 |
| GO:0010467 | gene expression | 31 / 800，3.88% | 0.004120592 |
| GO:0044270 | cellular nitrogen compound catabolic process | 22 / 177，12.43% | 8.45968E-11 |
| GO:0030162 | regulation of proteolysis | 8 / 110，7.27% | 0.006244026 |
| GO:0050819 | negative regulation of coagulation | 2 / 6，33.33% | 0.009112139 |
| GO:0050671 | positive regulation of lymphocyte proliferation | 3 / 7，42.86% | 0.000525303 |
| GO:0042102 | positive regulation of T cell proliferation | 2 / 5，40.00% | 0.006176684 |
| GO:1900047 | negative regulation of hemostasis | 2 / 4，50.00% | 0.003768307 |
| GO:0034655 | nucleobase-containing compound catabolic process | 21 / 164，12.80% | 1.46656E-10 |
| GO:0051649 | establishment of localization in cell | 27 / 494，5.47% | 2.7161E-05 |
| GO:0072376 | protein activation cascade | 2 / 5，40.00% | 0.006176684 |
| GO:0071887 | leukocyte apoptotic process | 2 / 6，33.33% | 0.009112139 |
| GO:0050670 | regulation of lymphocyte proliferation | 3 / 11，27.27% | 0.002300328 |
| GO:0051641 | cellular localization | 30 / 534，5.62% | 3.63982E-06 |
| GO:0009057 | macromolecule catabolic process | 23 / 296，7.77% | 3.37738E-07 |
| GO:0034613 | cellular protein localization | 25 / 306，8.17% | 2.82025E-08 |
| GO:0031638 | zymogen activation | 3 / 15，20.00% | 0.005893964 |
| GO:0044237 | cellular metabolic process | 50 / 1598，3.13% | 0.009671227 |
| GO:0022411 | cellular component disassembly | 20 / 156，12.82% | 4.49295E-10 |
| GO:0008104 | protein localization | 27 / 436，6.19% | 2.29211E-06 |
| GO:0071840 | cellular component organization or biogenesis | 37 / 964，3.84% | 0.001231634 |
| GO:0006613 | cotranslational protein targeting to membrane | 18 / 92，19.57% | 2.66734E-12 |
| GO:0009056 | catabolic process | 28 / 449，6.24% | 1.12054E-06 |
| GO:1901362 | organic cyclic compound biosynthetic process | 26 / 542，4.80% | 0.000417996 |
| GO:1902582 | single-organism intracellular transport | 23 / 392，5.87% | 4.79568E-05 |
| GO:0033365 | protein localization to organelle | 19 / 202，9.41% | 2.59614E-07 |
| GO:0072599 | establishment of protein localization to endoplasmic reticulum | 18 / 95，18.95% | 4.78008E-12 |
| GO:0016032 | viral process | 20 / 270，7.41% | 5.70305E-06 |
| GO:0019439 | aromatic compound catabolic process | 22 / 177，12.43% | 8.45968E-11 |
| GO:0006412 | translation | 21 / 249，8.43% | 3.32923E-07 |
| GO:0071822 | protein complex subunit organization | 25 / 380，6.58% | 2.17398E-06 |
| GO:0043933 | macromolecular complex subunit organization | 26 / 492，5.28% | 7.79816E-05 |
| GO:0018130 | heterocycle biosynthetic process | 25 / 519，4.82% | 0.000543658 |
| GO:0006414 | translational elongation | 18 / 87，20.69% | 9.56484E-13 |
| GO:0043241 | protein complex disassembly | 18 / 109，16.51% | 5.56274E-11 |
| GO:0070613 | regulation of protein processing | 2 / 4，50.00% | 0.003768307 |
| GO:0044260 | cellular macromolecule metabolic process | 42 / 1207，3.48% | 0.003483628 |
| GO:0046700 | heterocycle catabolic process | 22 / 175，12.57% | 6.70925E-11 |
| GO:0006402 | mRNA catabolic process | 19 / 125，15.20% | 6.55865E-11 |
| GO:0045861 | negative regulation of proteolysis | 4 / 30，13.33% | 0.006561486 |
| GO:1901575 | organic substance catabolic process | 27 / 427，6.32% | 1.49376E-06 |
| GO:0044765 | single-organism transport | 30 / 555，5.41% | 8.45688E-06 |
| GO:0008284 | positive regulation of cell proliferation | 7 / 80，8.75% | 0.003816573 |
| GO:0016071 | mRNA metabolic process | 21 / 331，6.34% | 3.63857E-05 |
| GO:0006810 | transport | 31 / 658，4.71% | 0.000101363 |
| GO:0034645 | cellular macromolecule biosynthetic process | 26 / 659，3.95% | 0.008485891 |
| GO:0006415 | translational termination | 18 / 81，22.22% | 2.53053E-13 |
| GO:0006605 | protein targeting | 19 / 193，9.84% | 1.23595E-07 |
| GO:0045047 | protein targeting to ER | 18 / 94，19.15% | 3.94526E-12 |
| GO:1902578 | single-organism localization | 32 / 584，5.48% | 2.29414E-06 |
| GO:0043624 | cellular protein complex disassembly | 18 / 101，7.82% | 1.43938E-11 |
| GO:0044267 | cellular protein metabolic process | 38 / 748，5.08% | 6.74184E-07 |
| GO:0061024 | membrane organization | 22 / 217，10.14% | 4.84711E-09 |
| GO:0090303 | positive regulation of wound healing | 2 / 4，50.00% | 0.003768307 |
| GO:0070661 | leukocyte proliferation | 25 / 308，8.12% | 0.008491779 |

| **Table S12. Significance enrichment analysis of differentially expressed proteins involved in biological processes in 12.5 μg/ml WSPM2.5 group.** | | | |
| --- | --- | --- | --- |
| Term | Description | Cluster frequency | p-value |
| GO:0007584 | response to nutrient | 3 / 29，10.34% | 0.002870207 |
| GO:0009617 | response to bacterium | 3 / 30，10.00% | 0.00316772 |
| GO:0071103 | DNA conformation change | 4 / 51，7.84% | 0.001519499 |
| GO:0014074 | response to purine-containing compound | 2 / 14，14.29% | 0.008420875 |
| GO:0098542 | defense response to other organism | 3 / 28，10.71% | 0.002590675 |
| GO:0006629 | lipid metabolic process | 6 / 187，3.21% | 0.009418536 |
| GO:0032787 | monocarboxylic acid metabolic process | 5 / 102，4.90% | 0.003080819 |
| GO:0010035 | response to inorganic substance | 4 / 66，6.06% | 0.003951658 |
| GO:0071824 | protein-DNA complex subunit organization | 4 / 35，11.43% | 0.000355497 |
| GO:0061448 | connective tissue development | 2 / 10，20.00% | 0.004269522 |
| GO:0044710 | single-organism metabolic process | 15 / 838，1.79% | 0.006988966 |
| GO:0051276 | chromosome organization | 6 / 141，4.26% | 0.002313169 |
| GO:0008202 | steroid metabolic process | 4 / 46，8.70% | 0.001027036 |
| GO:0031497 | chromatin assembly | 4 / 26，15.38% | 0.000107998 |
| GO:0034728 | nucleosome organization | 4 / 30，13.33% | 0.000192589 |
| GO:0019369 | arachidonic acid metabolic process | 2 / 14，14.29% | 0.008420875 |
| GO:0006325 | chromatin organization | 6 / 81，7.41% | 0.000115364 |
| GO:0042742 | defense response to bacterium | 2 / 3，66.67% | 0.000297392 |
| GO:0009636 | response to toxic substance | 3 / 22，13.64% | 0.001268077 |
| GO:0015718 | monocarboxylic acid transport | 2 / 14，14.29% | 0.008420875 |
| GO:0006323 | DNA packaging | 4 / 39，10.26% | 0.000543256 |
| GO:0006333 | chromatin assembly or disassembly | 4 / 32，12.50% | 0.000249251 |
| GO:0065004 | protein-DNA complex assembly | 4 / 28，14.29% | 0.000145885 |
| GO:0006334 | nucleosome assembly | 4 / 23，17.39% | 6.53037E-05 |
| GO:0006950 | response to stress | 12 / 513，2.34% | 0.00218753 |
| GO:1990267 | response to transition metal nanoparticle | 4 / 19，21.05% | 2.93833E-05 |

| **Table S13. Significance enrichment analysis of differentially expressed proteins involved in biological processes in 50 μg/ml WSPM2.5 group.** | | | |
| --- | --- | --- | --- |
| Term | Description | Cluster frequency | p-value |
| GO:1903707 | negative regulation of hemopoiesis | 2 / 18，11.11% | 0.008975042 |
| GO:0030099 | myeloid cell differentiation | 2 / 7，28.57% | 0.005575582 |
| GO:0006401 | RNA catabolic process | 4 / 34，11.76% | 7.89787E-05 |
| GO:0032984 | macromolecular complex disassembly | 11 / 132，8.33% | 0.000105637 |
| GO:0070727 | cellular macromolecule localization | 10 / 113，8.85% | 0.004386485 |
| GO:0045638 | negative regulation of myeloid cell differentiation | 14 / 308，4.55% | 0.008975042 |
| GO:0001676 | long-chain fatty acid metabolic process | 2 / 7，28.57% | 0.009623312 |
| GO:0072599 | establishment of protein localization to endoplasmic reticulum | 3 / 21，14.29% | 2.31243E-05 |
| GO:0071822 | protein complex subunit organization | 10 / 95，10.53% | 0.000604102 |
| GO:0072594 | establishment of protein localization to organelle | 18 / 380，4.74% | 0.00042551 |
| GO:0019083 | viral transcription | 12 / 186，6.45% | 3.33822E-05 |
| GO:0019080 | viral gene expression | 10 / 99，10.10% | 3.98346E-05 |
| GO:1902580 | single-organism cellular localization | 10 / 101，9.90% | 0.002879463 |
| GO:0006413 | translational initiation | 13 / 262，4.96% | 0.000105637 |
| GO:0000956 | nuclear-transcribed mRNA catabolic process | 10 / 113，8.85% | 3.48768E-05 |
| GO:0019058 | viral life cycle | 11 / 121，9.09% | 0.000387865 |
| GO:0006614 | SRP-dependent cotranslational protein targeting to membrane | 10 / 132，7.58% | 1.57065E-05 |
| GO:0034613 | cellular protein localization | 10 / 91，10.99% | 0.00412937 |
| GO:0045047 | protein targeting to ER | 14 / 306，4.58% | 2.1033E-05 |
| GO:0043624 | cellular protein complex disassembly | 10 / 94，10.64% | 3.98346E-05 |
| GO:0016071 | mRNA metabolic process | 10 / 101，9.90% | 0.008411773 |
| GO:0044270 | cellular nitrogen compound catabolic process | 14 / 331，4.23% | 0.000266777 |
| GO:0006414 | translational elongation | 12 / 177，6.78% | 1.0441E-05 |
| GO:0042989 | sequestering of actin monomers | 10 / 87，11.49% | 0.002673548 |
| GO:0006415 | translational termination | 2 / 4，50.00% | 5.40906E-06 |
| GO:0006886 | intracellular protein transport | 10 / 81，12.35% | 0.007070777 |
| GO:0044033 | multi-organism metabolic process | 12 / 257，4.67% | 4.34435E-05 |
| GO:0070972 | protein localization to endoplasmic reticulum | 10 / 102，9.80% | 3.0508E-05 |
| GO:0006613 | cotranslational protein targeting to membrane | 10 / 98，10.20% | 1.73349E-05 |
| GO:0043933 | macromolecular complex subunit organization | 10 / 92，10.87% | 0.005029162 |
| GO:0033559 | unsaturated fatty acid metabolic process | 19 / 492，3.86% | 0.001756116 |
| GO:0033365 | protein localization to organelle | 4 / 25，16.00% | 0.000907307 |
| GO:0044248 | cellular catabolic process | 12 / 202，5.94% | 0.002875572 |
| GO:0033036 | macromolecule localization | 17 / 397，4.28% | 0.006928558 |
| GO:0001503 | ossification | 19 / 506，3.75% | 0.001065496 |
| GO:0030097 | hemopoiesis | 4 / 22，18.18% | 0.009245735 |
| GO:0022411 | cellular component disassembly | 5 / 61，8.20% | 0.000357877 |
| GO:0046700 | heterocycle catabolic process | 11 / 156，7.05% | 0.000239426 |
| GO:0006612 | protein targeting to membrane | 12 / 175，6.86% | 5.15091E-05 |
| GO:0006402 | mRNA catabolic process | 10 / 104，9.62% | 4.74485E-05 |
| GO:0072657 | protein localization to membrane | 11 / 125，8.80% | 0.000739748 |
| GO:0034655 | nucleobase-containing compound catabolic process | 10 / 143，6.99% | 0.000553636 |
| GO:0000184 | nuclear-transcribed mRNA catabolic process, nonsense-mediated decay | 11 / 164，6.71% | 2.53923E-05 |
| GO:0006605 | protein targeting | 10 / 96，10.42% | 0.000599069 |
| GO:0043241 | protein complex disassembly | 12 / 193，6.22% | 7.74836E-05 |
| GO:0019439 | aromatic compound catabolic process | 10 / 109，9.17% | 0.001060721 |
| GO:0001649 | osteoblast differentiation | 11 / 177，6.21% | 0.003607769 |
| GO:0016482 | cytoplasmic transport | 3 / 15，20.00% | 0.005678037 |
| GO:1901361 | organic cyclic compound catabolic process | 13 / 283，4.59% | 0.000404625 |
| GO:0090150 | establishment of protein localization to membrane | 12 / 185，6.49% | 0.000412512 |
| GO:0050878 | regulation of body fluid levels | 10 / 133，7.52% | 0.007613623 |
| GO:0044265 | cellular macromolecule catabolic process | 7 / 108，6.48% | 0.008492439 |

| **Table S14. Significance enrichment analysis of differentially expressed proteins involved in biological processes in 200 μg/ml WSPM2.5 group.** | | | |
| --- | --- | --- | --- |
| Term | Description | Cluster frequency | p-value |
| GO:0032984 | macromolecular complex disassembly | 16 / 113，14.16% | 1.52777E-07 |
| GO:0045047 | protein targeting to ER | 15 / 94，15.96% | 7.7221E-08 |
| GO:0043933 | macromolecular complex subunit organization | 27 / 492，5.49% | 0.001029636 |
| GO:0061024 | membrane organization | 18 / 217，8.29% | 6.53873E-05 |
| GO:0042730 | fibrinolysis | 2 / 3，66.67% | 0.002785784 |
| GO:0000184 | nuclear-transcribed mRNA catabolic process, nonsense-mediated decay | 15 / 96，15.63% | 1.0353E-07 |
| GO:0030195 | negative regulation of blood coagulation | 2 / 4，50.00% | 0.005459679 |
| GO:0019439 | aromatic compound catabolic process | 15 / 177，8.47% | 0.000236212 |
| GO:0044033 | multi-organism metabolic process | 15 / 102，14.71% | 2.38608E-07 |
| GO:0046700 | heterocycle catabolic process | 16 / 175，9.14% | 5.55872E-05 |
| GO:0071822 | protein complex subunit organization | 25 / 380，6.58% | 9.66711E-05 |
| GO:0006415 | translational termination | 15 / 81，18.52% | 9.27097E-09 |
| GO:1901362 | organic cyclic compound biosynthetic process | 27 / 542，4.98% | 0.004584493 |
| GO:0016032 | viral process | 16 / 270，5.93% | 0.006988313 |
| GO:1900047 | negative regulation of hemostasis | 2 / 4，50.00% | 0.005459679 |
| GO:0006613 | cotranslational protein targeting to membrane | 15 / 92，16.30% | 5.71377E-08 |
| GO:0034655 | nucleobase-containing compound catabolic process | 15 / 164，9.15% | 9.87194E-05 |
| GO:0006412 | translation | 17 / 249，6.83% | 0.001145628 |
| GO:0006402 | mRNA catabolic process | 15 / 125，12.00% | 3.53782E-06 |
| GO:0044403 | symbiosis, encompassing mutualism through parasitism | 17 / 276，6.16% | 0.003565215 |
| GO:0061045 | negative regulation of wound healing | 2 / 5，40.00% | 0.008917145 |
| GO:0033365 | protein localization to organelle | 15 / 202，7.43% | 0.000994917 |
| GO:0006612 | protein targeting to membrane | 15 / 104，14.42% | 3.10849E-07 |
| GO:0072657 | protein localization to membrane | 17 / 143，11.89% | 7.96258E-07 |
| GO:0043241 | protein complex disassembly | 16 / 109，14.68% | 9.0538E-08 |
| GO:1902580 | single-organism cellular localization | 18 / 262，6.87% | 0.000734896 |
| GO:0045745 | positive regulation of G-protein coupled receptor protein signaling pathway | 2 / 4，50.00% | 0.005459679 |
| GO:0019080 | viral gene expression | 15 / 101，14.85% | 2.08529E-07 |
| GO:0072376 | protein activation cascade | 2 / 5，40.00% | 0.008917145 |
| GO:0070972 | protein localization to endoplasmic reticulum | 15 / 98，15.31% | 1.37745E-07 |
| GO:0006614 | SRP-dependent cotranslational protein targeting to membrane | 15 / 91，16.48% | 4.89958E-08 |
| GO:0044419 | interspecies interaction between organisms | 17 / 276，6.16% | 0.003565215 |
| GO:0061138 | morphogenesis of a branching epithelium | 3 / 15，20.00% | 0.009944468 |
| GO:0072594 | establishment of protein localization to organelle | 15 / 186，8.06% | 0.000409835 |
| GO:0007200 | phospholipase C-activating G-protein coupled receptor signaling pathway | 2 / 5，40.00% | 0.008917145 |
| GO:0045598 | regulation of fat cell differentiation | 2 / 3，66.67% | 0.002785784 |
| GO:0007528 | neuromuscular junction development | 2 / 5，40.00% | 0.008917145 |
| GO:0000956 | nuclear-transcribed mRNA catabolic process | 15 / 121，12.40% | 2.3241E-06 |
| GO:1901361 | organic cyclic compound catabolic process | 17 / 185，9.19% | 2.89416E-05 |
| GO:0044270 | cellular nitrogen compound catabolic process | 16 / 177，9.04% | 6.40022E-05 |
| GO:0044265 | cellular macromolecule catabolic process | 16 / 263，6.08% | 0.005403516 |
| GO:0008202 | steroid metabolic process | 6 / 46，13.04% | 0.00253287 |
| GO:0044764 | multi-organism cellular process | 17 / 271，6.27% | 0.002931356 |
| GO:0006401 | RNA catabolic process | 15 / 132，11.36% | 7.08228E-06 |
| GO:0019058 | viral life cycle | 15 / 132，11.36% | 7.08228E-06 |
| GO:0044802 | single-organism membrane organization | 18 / 211，8.53% | 4.47024E-05 |
| GO:0043624 | cellular protein complex disassembly | 16 / 101，15.84% | 2.94249E-08 |
| GO:0006414 | translational elongation | 15 / 87，17.24% | 2.59142E-08 |
| GO:0022411 | cellular component disassembly | 19 / 156，12.18% | 1.02909E-07 |
| GO:0090150 | establishment of protein localization to membrane | 15 / 133，11.28% | 7.78924E-06 |
| GO:0006605 | protein targeting | 15 / 193，7.77% | 0.000612493 |
| GO:0019083 | viral transcription | 15 / 99，15.15% | 1.58445E-07 |
| GO:0072599 | establishment of protein localization to endoplasmic reticulum | 15 / 95，15.79% | 8.95008E-08 |
| GO:0006413 | translational initiation | 15 / 113，13.27% | 9.47063E-07 |

| **Table S15. Significant pathway enrichment of differentially expressed proteins in the 12.5 μg/ml WSPM10 group.** | | |
| --- | --- | --- |
| Pathway_No. | Pathway_Name | p-value |
| hsa04614 | Renin-angiotensin system | 0.001143495 |
| hsa03460 | Fanconi anemia pathway | 0.03267184 |
| hsa00100 | Steroid biosynthesis | 0.04333219 |

| **Table S16. Significant pathway enrichment of differentially expressed proteins in the 50** **μg/ml WSPM10 group.** | | |
| --- | --- | --- |
| Pathway_No. | Pathway_Name | p-value |
| hsa03010 | Ribosome | 7.86821E-06 |
| hsa05144 | Malaria | 0.000512074 |
| hsa05143 | African trypanosomiasis | 0.002982921 |
| hsa05152 | Tuberculosis | 0.008428132 |
| hsa05150 | Staphylococcus aureus infection | 0.02282919 |
| hsa05203 | Viral carcinogenesis | 0.02409857 |
| hsa04115 | P53 signaling pathway | 0.02467332 |
| hsa05014 | Amyotrophic lateral sclerosis (ALS) | 0.02467332 |
| hsa05210 | Colorectal cancer | 0.02467332 |
| hsa04210 | Apoptosis | 0.03398624 |
| hsa03013 | RNA transport | 0.04425597 |
| hsa04012 | ErbB signaling pathway | 0.044441 |

| **Table S17. Significant pathway enrichment of differentially expressed proteins in the 200 μg/ml WSPM10 group.** | | |
| --- | --- | --- |
| Pathway_No. | Pathway_Name | p-value |
| hsa03010 | Ribosome | 5.09428E-14 |
| hsa04610 | Complement and coagulation cascades | 0.001915884 |

| **Table S18. Significant pathway enrichment of differentially expressed proteins in the 12.5 μg/ml WSPM2.5 group.** | | |
| --- | --- | --- |
| Pathway_No. | Pathway_Name | p-value |
| hsa05322 | Systemic lupus erythematosus | 1.82926E-05 |
| hsa05034 | Alcoholism | 9.20143E-05 |
| hsa05203 | Viral carcinogenesis | 0.000101664 |
| hsa04913 | Ovarian steroidogenesis | 0.001459253 |
| hsa05202 | Transcriptional misregulation in cancer | 0.005185787 |
| hsa04370 | VEGF signaling pathway | 0.009655968 |
| hsa00591 | Linoleic acid metabolism | 0.0101916 |
| hsa04062 | Chemokine signaling pathway | 0.02791129 |
| hsa00592 | alpha-Linolenic acid metabolism | 0.03027648 |
| hsa04014 | Ras signaling pathway | 0.0342255 |
| hsa00100 | Steroid biosynthesis | 0.04017162 |
| hsa05321 | Inflammatory bowel disease (IBD) | 0.04017162 |
| hsa04010 | MAPK signaling pathway | 0.04833198 |
| hsa05166 | HTLV-I infection | 0.04833198 |
| hsa00240 | Pyrimidine metabolism | 0.04833198 |
| hsa05030 | Cocaine addiction | 0.04996978 |
| hsa00140 | Steroid hormone biosynthesis | 0.04996978 |

| **Table S19. Significant pathway enrichment of differentially expressed proteins in the 50 μg/ml WSPM2.5 group.** | | |
| --- | --- | --- |
| Pathway_No. | Pathway_Name | p-value |
| hsa03010 | Ribosome | 4.4518E-06 |
| hsa00592 | alpha-Linolenic acid metabolism | 0.001355551 |
| hsa04913 | Ovarian steroidogenesis | 0.006500388 |
| hsa00591 | Linoleic acid metabolism | 0.0216062 |
| hsa04151 | PI3K-Akt signaling pathway | 0.02603656 |
| hsa04370 | VEGF signaling pathway | 0.04018736 |

| **Table S20. Significant pathway enrichment of differentially expressed proteins in the** **200** **μg/ml WSPM2.5 group.** | | |
| --- | --- | --- |
| Pathway_No. | Pathway_Name | p-value |
| hsa03010 | Ribosome | 1.87435E-08 |
| hsa00100 | Steroid biosynthesis | 0.005459679 |
| hsa05322 | Systemic lupus erythematosus | 0.01421545 |
| hsa04146 | Peroxisome | 0.028821 |
| hsa05150 | Staphylococcus aureus infection | 0.03098247 |
| hsa00533 | Glycosaminoglycan biosynthesis - keratan sulfate | 0.03098247 |
| hsa00601 | Glycosphingolipid biosynthesis -   lacto and neolacto series | 0.03098247 |

| **Table S21. Functional classification of verified differentially expressed proteins.** | | |
| --- | --- | --- |
| Functional classification | DEPs names | |
|  | WSPM10 | WSPM2.5 |
| 1 Basic metabolism-related enzymes | Hemoglobin subunit alpha;  ATPase ASNA1;  Sterol-4-alpha-carboxylate 3-dehydrogenase, decarboxylating;  Acyl-CoA desaturase;  rRNA 2'-O-methyltransferase fibrillarin | Hemoglobin subunit alpha;  Cytochrome P450 1B1;  Protein-glutamine gamma-glutamyltransferase 2;  Succinate dehydrogenase [ubiquinone] iron-sulfur subunit, mitochondrial |
| 2 Signal transduction-related proteins | Macrophage migration inhibitory factor;  Keratin, type I cytoskeletal 10;  COP9 signalosome complex subunit 6 | COP9 signalosome complex subunit 6;  Keratin, type II cuticular Hb1;  TIP41-like protein;  Cell division cycle protein 27 homolog;  Focal adhesion kinase 1; |
| - 3 Proteins associated with detoxification and transcriptional translation | Metallothionein-2;  Cleavage stimulation factor subunit 2;  Eukaryotic translation initiation factor 3 subunit I;  Proteasome inhibitor PI31 subunit;  Eukaryotic translation initiation factor 6;  Regulation of nuclear pre-mRNA domain-containing protein 1A | Metallothionein-2;  Small nuclear ribonucleoprotein Sm D1;  ADP-ribosylation factor-like protein 6-interacting protein 4;  Cleavage stimulation factor subunit 2;  Proteasome subunit beta type-6;  Regulation of nuclear pre-mRNA domain-containing protein 1A |
| 4 Ribosome associated proteins | 60S ribosomal protein L18a;  60S ribosomal protein L14;  28S ribosomal protein S7,mitochondrial;  60S ribosomal protein L38;  60S ribosomal protein L18a;  60S ribosomal protein L27;  60S ribosomal protein L6;  60S ribosomal protein L27a;  40S ribosomal protein S13;  40S ribosomal protein S2;  40S ribosomal protein S25;  60S ribosomal protein L8;  60S ribosomal protein L19;  60S ribosomal protein L36;  60S ribosomal protein L22-like 1;  60S ribosomal protein L34;  60S ribosomal protein L24 | 60S ribosomal protein L38;  60S ribosomal protein L27;  60S ribosomal protein L14;  60S ribosomal protein L7a;  40S ribosomal protein S18;  60S ribosomal protein L13;  60S ribosomal protein L6;  60S ribosomal protein L18a |
| 5 Calcium binding protein | Calponin-3;  Tetranectin | Protein S100-A11 |
| 6 Cellular structure-related proteins |  | Golgi resident protein GCP60;  Radixin;  Proteasome adapter and scaffold protein ECM29;  Paxillin;  Histone H4;  Mitochondrial import inner membrane translocase subunit Tim13;  Endoplasmic reticulum resident protein 44 |

| **Table S22. Expression of Several Cell cycle regulators in A549 Cells Exposed to the WSPM, NaF, CeCl_3_ or LaCl*_3_.*** | | | | | | | | | | | | | | |
| --- | --- | --- | --- | --- | --- | --- | --- | --- | --- | --- | --- | --- | --- | --- |
| Protein name | WSPM10  （μg/ml） | |  | WSPM2.5  （μg/ml） | |  | NaF  （mM） | |  | CeCl_3_  （mM） | |  | LaCl*_3_*  （mM） | |
|  | 100 | 200 |  | 100 | 200 |  | 2 | 5 |  | 1 | 2 |  | 1 | 2 |
| ATM | 3.80 | 4.98 |  | 4.10 | 3.36 |  | 4.24 | 3.71 |  | 6.87 | 1.62 |  | 1.33 | 0.63 |
| CDK2 | 1.47 | 1.59 |  | 0.98 | 1.18 |  | 0.67 | 0.46 |  | 1.49 | 0.48 |  | 0.93 | 0.32 |
| CDK4 | 1.38 | 1.64 |  | 1.24 | 1.13 |  | 1.02 | 0.10 |  | 1.29 | 0.37 |  | 0.93 | 0.44 |
| E2F1 | 4.41 | 5.34 |  | 2.87 | 2.92 |  | 4.39 | 3.77 |  | 5.37 | 4.99 |  | 3.68 | 2.34 |
| Rb1 | 1.35 | 1.52 |  | 1.16 | 1.35 |  | 1.58 | 0.96 |  | 2.15 | 1.18 |  | 1.64 | 1.22 |
| MDM2 | 1.01 | 1.08 |  | 0.64 | 0.97 |  | 0.69 | 0.60 |  | 1.03 | 0.88 |  | 0.93 | 0.84 |
| P53 | 1.06 | 1.47 |  | 1.08 | 1.07 |  | 1.05 | 0.82 |  | 1.50 | 1.19 |  | 1.57 | 1.46 |
| P21 | 1.81 | 1.33 |  | 1.14 | 1.76 |  | 1.20 | 1.09 |  | 3.98 | 2.06 |  | 7.10 | 4.03 |
